# Segmental Chiropractic Spinal Manipulation Does not Reduce Pain Amplification and the Associated Pain-Related Brain Activity in a Capsaicin-Heat Pain Model

**DOI:** 10.3389/fpain.2021.733727

**Published:** 2021-11-01

**Authors:** Benjamin Provencher, Stéphane Northon, Mathieu Piché

**Affiliations:** ^1^Pain Neurophysiology Lab, Department of Anatomy, Université du Québec à Trois-Rivières, Trois-Rivières, QC, Canada; ^2^CogNAC Research Group, Université du Québec à Trois-Rivières, Trois-Rivières, QC, Canada

**Keywords:** SMT, pain modulation, spine, pain, manual therapy

## Abstract

Musculoskeletal injuries lead to sensitization of nociceptors and primary hyperalgesia (hypersensitivity to painful stimuli). This occurs with back injuries, which are associated with acute pain and increased pain sensitivity at the site of injury. In some cases, back pain persists and leads to central sensitization and chronic pain. Thus, reducing primary hyperalgesia to prevent central sensitization may limit the transition from acute to chronic back pain. It has been shown that spinal manipulation (SM) reduces experimental and clinical pain, but the effect of SM on primary hyperalgesia and hypersensitivity to painful stimuli remains unclear. The goal of the present study was to investigate the effect of SM on pain hypersensitivity using a capsaicin-heat pain model. Laser stimulation was used to evoke heat pain and the associated brain activity, which were measured to assess their modulation by SM. Eighty healthy participants were recruited and randomly assigned to one of the four experimental groups: inert cream and no intervention; capsaicin cream and no intervention; capsaicin cream and SM at T7; capsaicin cream and placebo. Inert or capsaicin cream (1%) was applied to the T9 area. SM or placebo were performed 25 min after cream application. A series of laser stimuli were delivered on the area of cream application (1) before cream application, (2) after cream application but before SM or placebo, and (3) after SM or placebo. Capsaicin cream induced a significant increase in laser pain (*p* < 0.001) and laser-evoked potential amplitude (*p* < 0.001). However, SM did not decrease the amplification of laser pain or laser-evoked potentials by capsaicin. These results indicate that segmental SM does not reduce pain hypersensitivity and the associated pain-related brain activity in a capsaicin-heat pain model.

## Introduction

Musculoskeletal injuries generally lead to an inflammatory response, peripheral sensitization and primary hyperalgesia (1). This occurs with low back injuries, which are associated with acute pain and increased pain sensitivity at the site of injury, and in some cases, this leads to chronic primary low back pain (2–4).

Primary hyperalgesia mostly results from peripheral mechanisms and is caused by nociceptor sensitization (5). The release of pro-inflammatory mediators, including serotonin, bradykinin, prostaglandins, arachidonic acid and substance P was shown to cause nociceptor sensitization in the injured tissues (5, 6). Thus, primary hyperalgesia is limited to the site of injury and is characterized by a decrease in pain threshold, an increased response to suprathreshold stimuli and spontaneous pain in the absence of external stimulation (5–7). When primary hyperalgesia affects the hairy skin, both Aδ and C nociceptors are sensitized to heat stimuli (5, 6, 8). If primary hyperalgesia persists, sustained nociceptive inputs from C-nociceptors to spinal cord neurons lead to central sensitization, which can be evidenced by the presence of secondary hyperalgesia (6, 9, 10). Secondary hyperalgesia is usually described as an increased sensitivity to mechanical, but not heat stimuli, in uninjured tissues outside the area of tissue injury (6, 7, 11). Recent studies have shown that this relies on a class of A-nociceptors that project to sensitized spinal neurons in laminae I and IV/V (10, 12, 13). Central sensitization is common in chronic pain conditions (14). Features of central sensitization such as increased temporal summation and decreased pressure pain threshold in remote body parts (secondary hyperalgesia) are also frequently observed in chronic low back pain (15). Thus, reducing the intensity or the duration of primary hyperalgesia to prevent central sensitization and secondary hyperalgesia may limit the transition from acute to chronic pain.

Spinal manipulation (SM) is a form of manual therapy provided by different healthcare professionals, including chiropractors (16). SM has been shown to reduce both experimental and clinical pain (17, 18) and recent clinical practice guidelines recommend the use of SM for the management of back pain (19–22). Although several studies suggest that SM may decrease pain via segmental mechanisms involving the processing of C-nociceptor inputs in the spinal cord (23–26), these mechanisms are not fully understood, and the effect of SM on primary hyperalgesia remains unclear.

Two systematic reviews reported that SM decreases experimental pain in healthy volunteers (17, 18). Accordingly, several experimental studies demonstrated that SM could decrease experimental cutaneous pain in healthy volunteers (23, 24, 27–30) and in patients with low back pain (25, 26), when the nociceptive activity is amplified by centrally mediated mechanisms like temporal summation and central sensitization. In addition, a recent meta-analysis reported that physical therapy (including manual therapy such as SM) improves nociceptive processing influenced by or related to central sensitization in patients with chronic musculoskeletal pain (31). Although it has never been demonstrated using neurophysiological measures, these findings suggest that SM affects the transmission of nociceptive activity or decreases nociceptive transmission in the dorsal horns of the spinal cord, regardless of the origin of the inputs (cutaneous or myofascial). Besides, a few studies suggest that the hypoalgesic effects of SM are partly due to decreased rigidity of the spine (32, 33). In these studies, however, SM were not performed specifically at one segment. Moreover, a recent study suggests that spinal stiffness is not the most important factor to produce SM-induced hypoalgesia (34). Furthermore, no clinically relevant association was observed between spine stiffness and mechanical pain thresholds (35), where increased stiffness was not associated with increased mechanical pain sensitivity, but rather with increased pain thresholds (lower pain sensitivity). Thus, although mechanical effects cannot be excluded, the hypoalgesic effects of SM may be independent of the improvement in mechanical function and may be produced when applied to joints without restriction, therefore supporting the observed effects in healthy volunteers.

Capsaicin pain models are widely used in pain research for the selective activation of nociceptors (36–38). Capsaicin is a chemical that is naturally present in chili peppers that binds to transient receptor potential vanilloid 1 (TRPV1), which is highly expressed in C-nociceptors (39). Low concentrations ( ≤1%) of capsaicin applied topically initially excite C-nociceptors, resulting in a transient inflammatory response, pain, as well as primary and secondary hyperalgesia (6, 7, 11, 39). To our knowledge, two studies investigated the hypoalgesic mechanisms of SM on capsaicin pain, one reporting that a single SM session reduced capsaicin pain and the other showing no effect (30, 40). In addition to these conflicting findings that remain to be clarified, the underlying neurophysiological mechanisms of SM on heat pain amplification by capsaicin remain unknown.

The aim of the present study was to investigate the effects of SM on pain amplification using a capsaicin-heat pain model, in which laser pulses are applied on capsaicin-treated skin. We hypothesized that segmental SM would reduce the capsaicin-induced amplification of laser heat pain, by reducing the associated increase in spinal nociceptive transmission. We also hypothesized that this reduction would result in reduced laser-evoked potentials amplitude, partly reflecting the segmental inhibition of spinal nociceptive activity.

## Materials and Methods

### Participants

**Figure 1:**
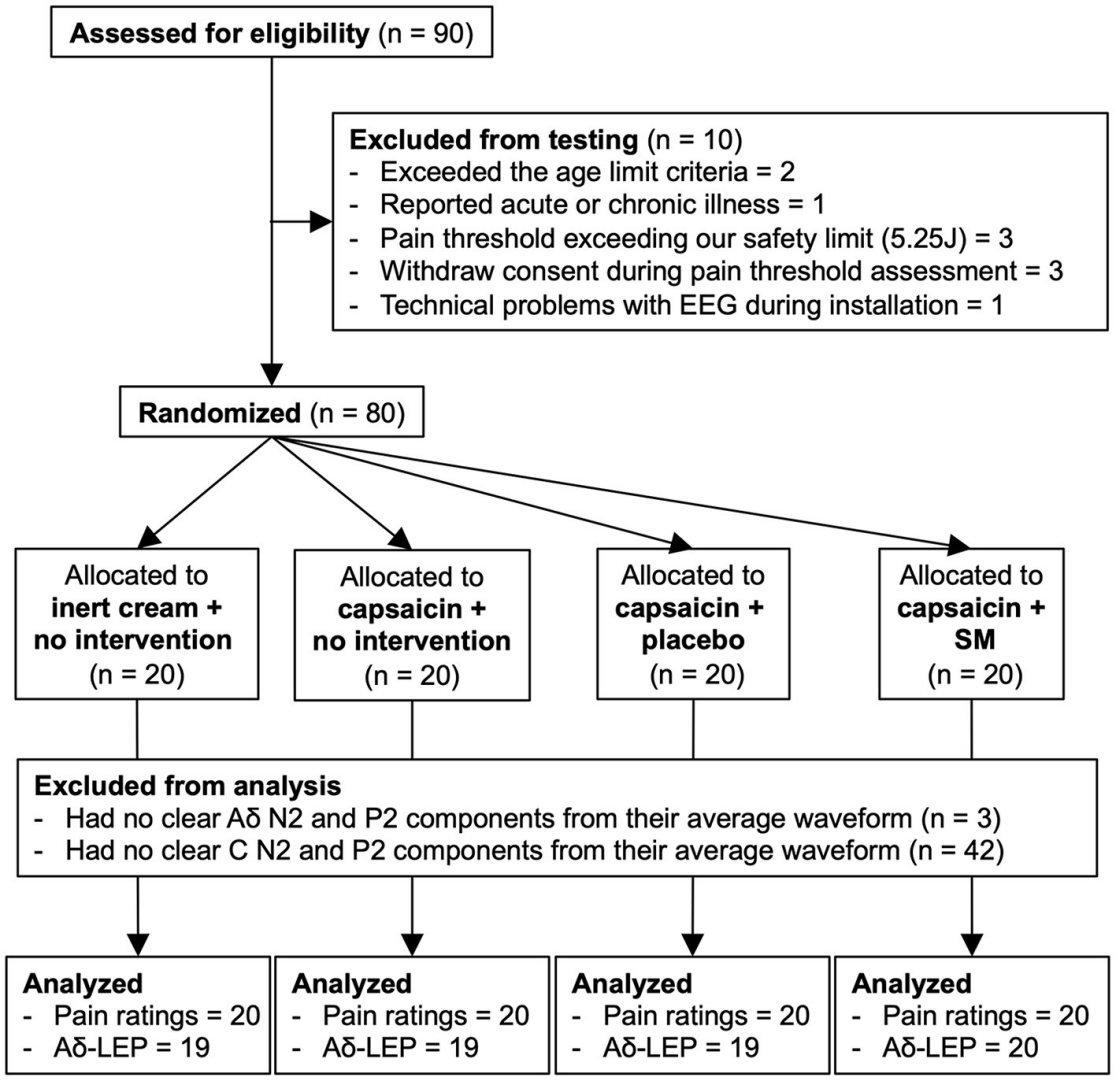
Flow diagram of enrollment, allocation, and analyses. SM, spinal manipulation; LEP, laser-evoked potential.

Ninety participants were recruited by advertisement on the campus of Université du Québec à Trois-Rivières and on social media. Participants were included if they were between 18 and 55 years old. They were excluded if they reported acute or chronic pain, acute or chronic illness, psychiatric disorders, if they underwent spinal surgery, had a significant injury to the spine in the 3 months preceding the experiment, or took any medication or recreational drug during the 2 weeks prior to experimentation. They were also excluded if they presented a skin of type I on the Fitzpatrick scale, reported having an allergy/intolerance to chili peppers, or if their pain threshold exceeded the safety limit for laser stimulation (see section Painful Laser Stimulation). Eighty healthy volunteers [40 women and 40 men; aged 27.1 ± 6.8 years (mean ± SD)] were included and completed the study. A flow diagram of participants inclusion is presented in Figure 1. All experimental procedures conformed to the standards set by the latest revision of the Declaration of Helsinki and were approved by the Research Ethics Board of Université du Québec à Trois-Rivières. All participants gave written informed consent, acknowledging their right to withdraw from the experiment without prejudice, and received a compensation of $25 for their travel expenses, time and commitment.

### Experimental Design and Protocol

This study relied on a mixed design to compare changes in laser-evoked pain and brain activity between groups. Participants were randomly assigned to one of the four experimental groups using a random-number generator: inert cream with no intervention (*n* = 20); capsaicin cream and no intervention (*n* = 20); capsaicin cream and SM at T7 (*n* = 20); capsaicin cream and placebo intervention (*n* = 20).

During the experiment, room temperature was kept constant at 24°C. All participants were seated on a regular desk chair with a low backrest (under T9 vertebra) during the first 25 min of the experiment, after which they were instructed to lay prone comfortably on a chiropractic table for the remaining 10 min. This allowed the intervention to be delivered. Their head was slightly elevated by a thick towel placed under their chin to avoid pressure on the electrooculography (EOG) and frontal electroencephalography (EEG) electrodes. The participant and experimenter wore safety glasses designed for a 1,340 nm wavelength laser at all time. In both positions, participants were instructed to keep their eyes open, look at a fixation cross to minimize eye movement, and refrain movement as much as possible during stimulation.

**Figure 2:**
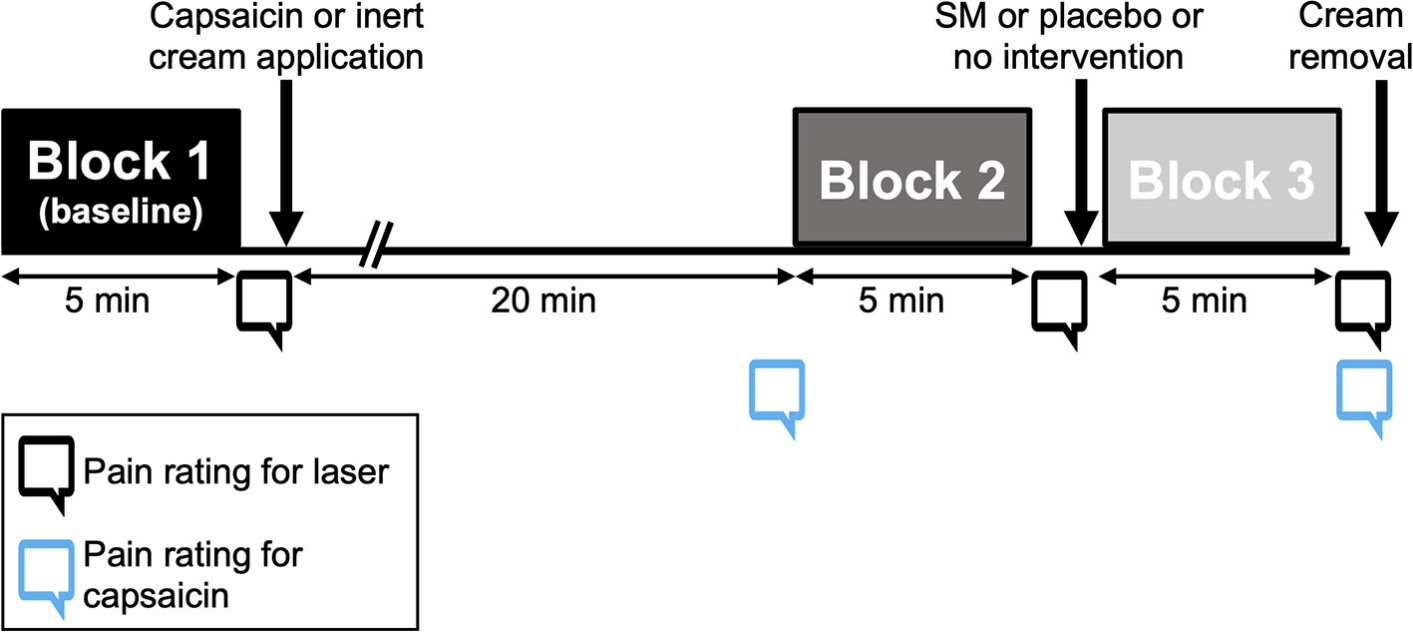
Experimental protocol. Three blocks of 30 laser stimuli were applied to the back, for a total of 90 stimuli. After each block, participants were instructed to rate pain verbally. Inert or capsaicin cream was applied to the stimulation area after block 1 and removed at the end of the experiment. Between blocks 2 and 3, participants received either no intervention, a placebo intervention or a spinal manipulation at T7.

The experimental protocol is illustrated in Figure 2. The experiment comprised three blocks of 30 laser stimuli delivered to the T9 area. After the first block, inert or capsaicin cream (1%) was applied to the stimulation area and participants waited 20 min before the next block of laser stimuli. After this second block (25 min after cream application), either no intervention, SM or the placebo intervention (sham SM) were performed. For the groups with no intervention, participants simply waited for 60 s before the third block of laser stimuli was delivered. After each stimulation block, participants provided their pain ratings, and they could blink and stretch as needed. The timing of the intervention (25 min after cream application) was based on previous findings, which characterized the time course of tonic pain produced by capsaicin applied to the back (30). After 20 min, capsaicin pain remains relatively stable, which allows to compare laser pain with baseline and pain amplification by capsaicin. This is more clinically relevant to investigate the hypoalgesic mechanisms of SM, since ongoing tonic pain is closer to what patients report with acute back pain. The laser stimulus allows confirming the amplification of pain by capsaicin and the measure of laser evoked potentials and their amplification by capsaicin.

### Painful Laser Stimulation

Painful stimuli were produced by laser heat pulses generated by an infrared neodymium-doped yttrium aluminum perovskite laser (Nd:YAP, DEKA 1,340, Electronical Engineering, Florence, Italy). These stimuli have been shown to activate heat sensitive Aδ and C fibers selectively, while avoiding the activation of Aβ fibers (41, 42). The laser beam was transmitted through a 10 m optic fiber cable and was adjusted to 7 mm (≈38.5 mm^2^ area), with a pulse duration of 5 ms. Based on our prior experience with this type of laser and safety recommendations for repeated laser stimuli (43), a maximum fluence limit was set to 14 J/cm^2^ (5.25 J intensity limit for a 7 mm laser beam diameter). The laser device was triggered using a stimulus presentation software (Spike2; Cambridge Electronic Design Limited, Cambridge, UK). To avoid stimulation of the same area twice, 25 tiny ink marks were drawn on the skin with a regular Hi-Tecpoint 0.5 mm—Black Pilot pen to guide the experimenter for the area to be stimulated, in a 5 × 5 cm grid centered around T9 spinous process (T9-T10 dermatome). The laser was moved to the next point of the grid after each stimulus. No adverse event related to laser stimulation was observed by the experimenter during the experiment or reported by participants after the experiment.

The individual pain threshold was determined using a staircase procedure. Before pain threshold assessment, participants were instructed to focus on the warm/burning sensation in their back and to report pain intensity verbally after each stimulus using a numerical rating scale ranging from 0 to 100, 0 indicating “no pain” and 100 “the worse pain imaginable.” Stimuli were delivered at an initial intensity of 0.5 J and stimulus intensity increased sequentially by 0.5 J increments until pain was reported (rating of 1/100 or higher), or until the 5.25 J safety limit was reached. If no pain was reported (rating of 0/100) at the highest energy laser stimulus within our safety limits (5.25 J), the participant was excluded from the study. This was necessary for the purpose of the study, in which we examined pain inhibition and not only LEPs. Three participants were excluded for this reason. Otherwise, the energy was increased sequentially again until a pain rating of at least 30/100 was reported or until the 5.25 J limit was reached. Participants were then familiarized with the selected intensity using five consecutive stimuli with an interstimulus interval varying between 5 and 10 s. If the intensity was deemed acceptable for the participant, the experiment was continued. If the participant judged that the stimulus intensity produced pain that could not be tolerated for the duration of the experiment, stimulus intensity was decreased by 0.5 J and the familiarization procedure resumed until an acceptable stimulus intensity was reached. Once approved by the participant, the stimulus intensity was kept constant throughout the experiment.

### Capsaicin and Inert Creams

The 1% capsaicin cream used for this experiment was prepared by a pharmaceutical laboratory (Gentès & Bolduc pharmacists, St-Hyacinthe, QC, Canada). This concentration was used to produce tonic pain and hyperalgesia in prior studies (40, 44–46). The inert cream consisted of the same petroleum jelly-based cream without the active ingredient. After the first laser stimulation block, ≈1 g of cream was applied uniformly to the stimulated area in a layer <1 mm thick and covered with transparent food-grade plastic film secured in place by adhesive tape. This ensured that the cream could not be wiped or spread out. After the experiment, the plastic film was discarded, the remaining cream removed, and the participants back carefully washed with water and soap to eliminate any trace of the cream. The burning sensation induced by capsaicin vanished within 30–120 min after the cream removal.

Instructions given to the participants were scripted for standardization and to minimize the influence of expectations. Participants were told which cream would be applied to their back (inert or capsaicin). However, they were not aware that other participants may receive a different cream. Moreover, none of the participants had used capsaicin cream before. Therefore, they were not aware that it could affect laser-evoked pain. Prior to the experiment, the information given to each participant was as follows: “*After the first series of laser stimuli, we will apply 1 g of inert cream in your back. This will not do anything*” OR “*After the first series of laser stimuli, we will apply 1 g of capsaicin cream in your back. You may feel nothing at all, or you may notice a warm or burning sensation. These sensations are completely normal. We are interested to know how you will react to this cream*.” Later, when it was time to rate the spontaneous pain induced by capsaicin, the experimenter asked the following question: 1- Do you feel something in your back right now? If the participant said yes: 2- Do you consider this painful? If the participant said yes: 3- How painful is it from 0 to 100?

### Spinal Manipulation and Placebo Intervention

SM were performed by only one licensed chiropractor and consisted of a preload force followed by a short-duration, high-velocity, low amplitude, posterior to anterior thrust applied with both hands. SM was applied over the transverse processes of T7 vertebra, just outside the area where cream was applied, to generate audible release (cavitation). The contact point at T7-T8 for SM was selected to avoid painful SM due to the application of this mechanical force on the acutely inflamed and sensitized T9 area. SM directly applied at T9 may produce excitatory activity that could counter hypoalgesic effects. Moreover, since a single thrust affects several adjacent segments (47), SM applied as close as possible to the painful area while being outside the region of hypersensitivity would produce the most favorable outcomes. This type of manipulation lasts <200 ms and involves a force of ~500 N (48–51). The placebo intervention consisted of a sham spinal manipulation performed with the participant in the same position, but the chiropractor contacted the medial border of both scapulae to apply a preload force by moving the scapulae laterally. A high-velocity, low amplitude thrust was then applied in the scapula-thoracic plane. This type of sham manipulation has been used in previous studies and reported successful in blinding most of the participants (52, 53). No adverse event was reported by participants following SM or placebo.

### Pain Ratings

After each series of 30 stimuli, participants were instructed to rate laser-induced pain verbally using a numerical rating scale ranging from 0 to 100, 0 indicating “no pain” and 100 “worse pain imaginable.” They were instructed to report the average pain induced by the 30 stimuli. Immediately before the second stimulation block (20 min after cream application), and immediately after the third stimulation block (30 min after cream application), participants with the capsaicin cream were also instructed to rate capsaicin-induced pain using the same pain rating scale.

### Expectations of Pain Modulation and Blinding

To limit the effects of expectations, participants were not informed about the specific objective of the study or about the different groups receiving different interventions (SM or sham SM). They were informed that their back would be mechanically stimulated and that we were interested in measuring how this mechanical stimulus may influence pain-related brain activity. Expectations of pain modulation were measured using a visual analog scale (54). Before the second block of laser stimuli, participants were presented a form with the following question: “On the scale below, indicate the change in laser-pain intensity that you expect following the intervention in your back.” The scale was a horizontal line ranging from −100 to 100 with the following anchors: −100 = “maximum decrease,” 0 = “no change” and 100 = “maximum increase.” Although participants were not aware that they belonged to different groups, we examined “blinding” by asking the following question to the two intervention groups: “Do you believe that the intervention you received was effective?”

### Electroencephalographic Recordings

Electroencephalography (EEG) was recorded using a 64-channel BrainVision system with active Ag-AgCl electrodes mounted on an actiCAP, according to the International 10–20 system (Brain Products, Gilching, Germany). Electrodes were nose-referenced, and the ground was set at FPz. Signals were sampled at 1,000 Hz and filtered using a 0.01–100 Hz band-pass filter. Eye movements and blinks were recorded using right eye EOG with electrodes placed at the suborbital ridge and just lateral to the external ocular canthus. Electrode impedance was kept below 20 kΩ.

### Laser-Evoked Potentials Analyses

EEG signals were analyzed offline using EEGLAB v2020.0 (55). After applying a 0.5–30 Hz finite impulse response band-pass filter (56–58), data were segmented into epochs extending from −100 ms to +1,500 ms relative to stimulus onset (56, 57, 59, 60). Epochs were baseline corrected using the −100 to 0 ms window (56, 58, 60) and then visually inspected to reject epochs with artifacts (amplitude value exceeding ± 100 μV) (56–58, 61). On average 3.2 ± 2.4 out of 90 epochs (3.5%) were rejected. Data were then re-referenced to the common average and, an Infomax independent component analysis (ICA) was applied using the in-built EEGLAB function Runica to identify and remove components associated with noise (eye movements, eye blinks, cardiac and muscle artifacts) (55).

After data pre-processing, average waveforms were computed for each participant and stimulation block, and LEP components of interest were analyzed, including the Aδ and C fibers N2 and P2 (60–62). The Aδ-N2 was defined as the first major negative deflection occurring between 150 and 400 ms with a maximum amplitude at the vertex (Cz), and the Aδ-P2 was defined as the first major positive deflection occurring between 250 and 500 ms with a maximum amplitude at the vertex (Cz). The C-N2 was defined as the first major negative deflection following the Aδ-P2 and occurring between 350 and 1,500 ms with a maximum amplitude at the vertex (Cz). The C-P2 was defined as the first major positive deflection following the C-N2 and occurring between 400 and 1,500 ms with a maximum amplitude at the vertex (Cz). An independent assessment performed by two of the experimenters (BP and MP) revealed that from the 80 participants tested, 3 (≈4%) did not have clear Aδ-N2 and Aδ-P2 peaks from their average waveforms and a majority of participants did not have clear C-N2 and C-P2 peaks. Thus, the N2 and P2 calculations were performed on data from the remaining 77 participants for Aδ fibers and C-LEP analyses are not presented. However, all data are shown in Supplementary Figure 1. For the LEP amplitude analysis, the N2-P2 peak-to-peak amplitude was preferred for quantification to limit inter-individual amplitude variability; in some participants, the N2 was of normal amplitude and latency, normal topographic distribution, but was shifted from baseline, leading to incorrect amplitude measurements. The peak-to-peak analysis counter this issue although the potential effects on specific components cannot be examined. This was a fair compromise since modulation of LEPs in this study is presumed to occur due to spinal modulation of nociceptive transmission and thus to mostly reflect the resulting ascending information.

### Statistical Analysis

Statistical analyses were conducted using Statistica v13.5 (Kivuto Solutions Inc., Ottawa, ON, Canada). All results are expressed as mean ± SD. SD values were corrected to remove between-subject variability (63). Statistical threshold was set at *p* < 0.05. Data distribution was assessed for normality with the Kolmogorov-Smirnov test and homogeneity of variance was assessed using Levene's test. To assess if the amplification of pain and nociceptive brain responses was induced by capsaicin, the data from block 2 relative to block 1 (difference) were compared between the four groups using one-way ANOVAs. To examine the effects of SM on capsaicin pain, and the amplification of pain and nociceptive brain responses, mixed ANOVAs were performed. These ANOVAs comprised 2 factors: groups (the 3 groups with capsaicin cream) and time (block 2 and 3). Bonferroni-corrected planned contrasts were used to decompose significant effects. Effect sizes are reported based on partial eta-squared ($\eta_{p}^{2}$).

## Results

### Laser Stimulation Intensity and Capsaicin Pain

The mean laser stimulation intensity for each group was 4.6 ± 1.0 J for inert cream with no intervention, 4.9 ± 0.5 J for capsaicin cream and no intervention, 4.6 ± 1.0 J for capsaicin cream and SM, and 4.8 ± 0.7 J for capsaicin cream and placebo intervention. A one-way ANOVA revealed no significant difference between groups (F_3,76_ = 0.81, *p* = 0.49; $\eta_{p}^{2}$ = 0.03).

**Figure 3:**
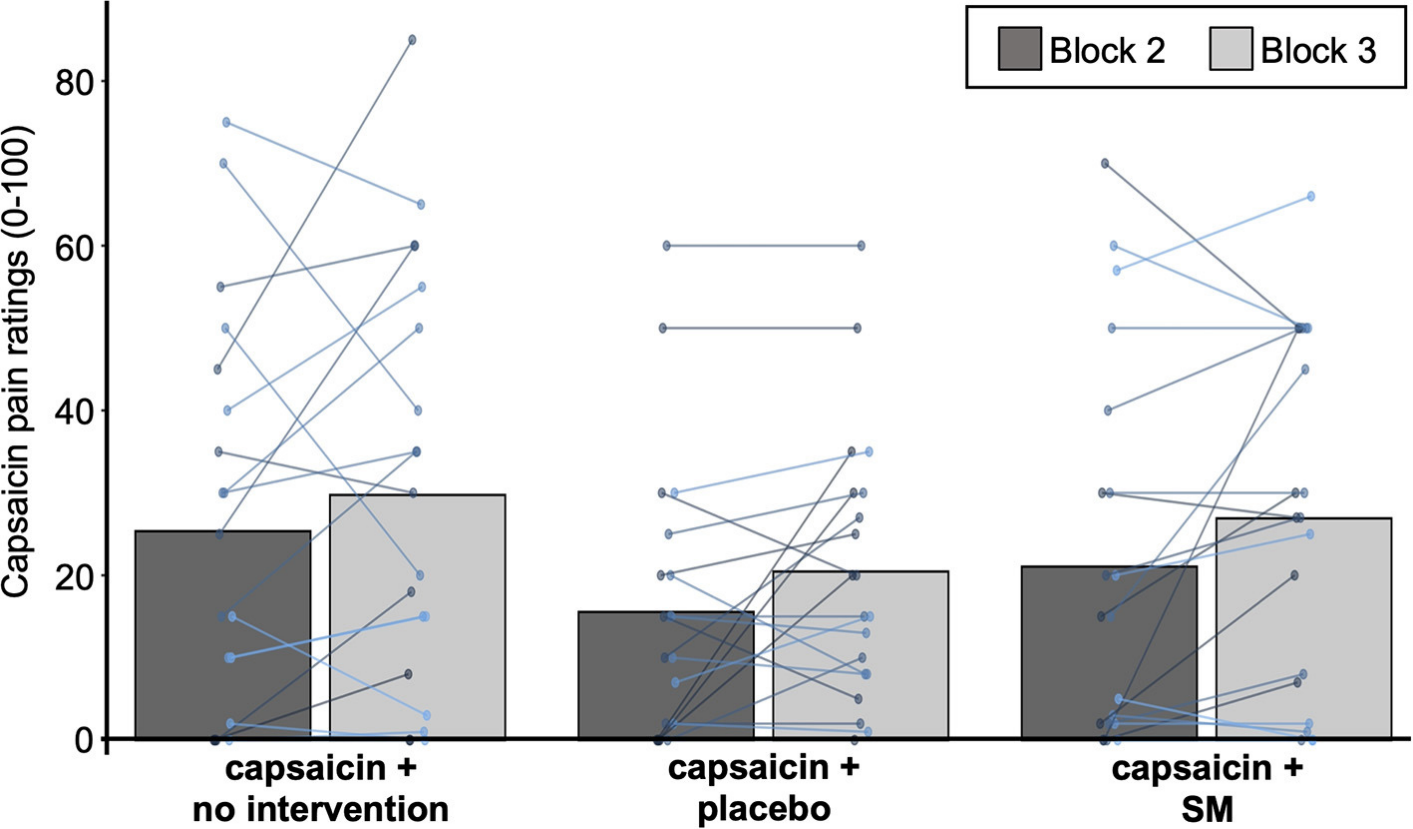
Capsaicin pain ratings. Mean pain ratings for capsaicin pain for the three groups with capsaicin cream. Data from each participant are represented by linked colored points and the mean of these data points for each block is represented by gray bars.

Capsaicin pain ratings are reported in Table 1 and presented in Figure 3. Capsaicin pain was not significantly different between groups (main effect: F_2,50_ = 2.3, *p* = 0.11, $\eta_{p}^{2}$ = 0.09) or between groups over time (interaction: F_2,50_ = 0.6, *p* = 0.58, $\eta_{p}^{2}$ = 0.02). This indicates that SM did not reduce capsaicin pain.

**Table 1:** Pain ratings for the four experimental groups (mean ± SD).

		**Inert cream**	**Capsaicin cream**		
		**No intervention**	**No intervention**	**Placebo**	**SM**
Laser pain	Block 1	22.5 ± 4.8	21.8 ± 12.3	15.9 ± 8.2	24.5 ± 14.3
	Block 2	15.2 ± 2.1	47.3 ± 6.5	32.0 ± 5.0	45.3 ± 8.3
	Block 3	11.8 ± 4.0	51.1 ± 7.9	32.0 ± 5.0	48.0 ± 7.6
Capsaicin pain	Block 2		25.4 ± 8.9	15.6 ± 6.2	21.1 ± 7.4
	Block 3		29.8 ± 8.9	20.5 ± 6.2	26.9 ± 7.4

### Heat Pain Amplification Induced by Capsaicin

Pain ratings for laser stimulation are reported in Table 1. Out of the 60 participants on which capsaicin cream was applied, 54 (90%) reported increased sensitivity to laser stimuli during the second block. This number increased to 57 participants (95%) during the third stimulation block. Thus, 3/60 participants (2 in SM and 1 in placebo intervention group) did not show heat pain amplification during the experiment.

**Figure 4:**
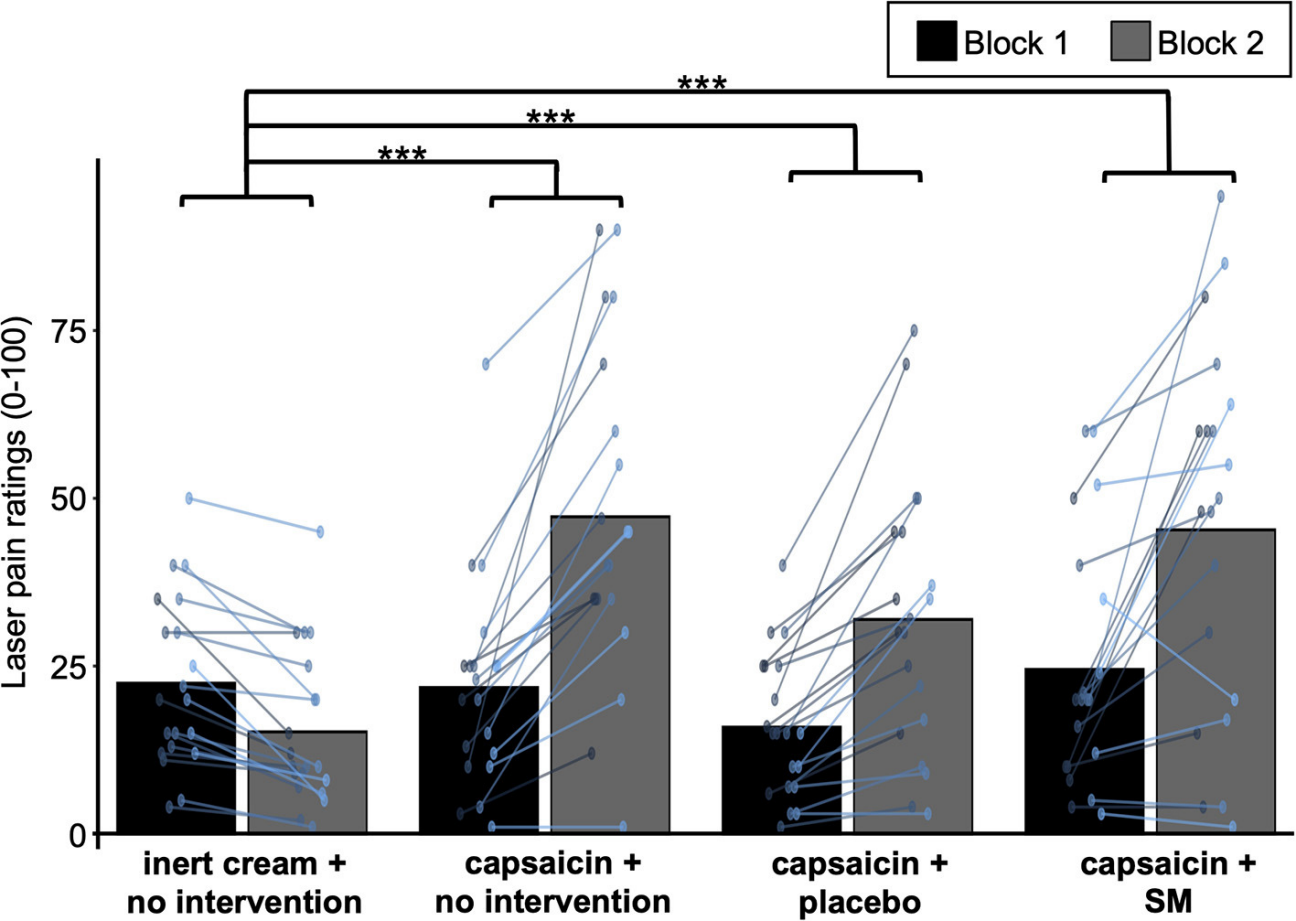
Heat pain amplification induced by capsaicin. Mean laser pain ratings during block 1 and block 2 (before and after cream application, but before the intervention) for the four experimental groups. Data from each participant are represented by colored points and the mean of these data points for each block is represented by gray bars. ***P < 0.001.

To confirm that capsaicin induced heat pain amplification, changes in laser pain after the cream application but before any intervention were compared between groups using a one-way ANOVA (see Figure 4). The change in laser pain was significantly different between groups (F_3,76_ = 16.7, *p* < 0.0001, $\eta_{p}^{2}$ = 0.40). Bonferroni-corrected planned contrasts revealed that the change in laser pain was greater in the three groups with capsaicin cream compared with the group with inert cream (all *p* < 0.001).

### Effects of SM on Heat Pain Amplification

**Figure 5:**
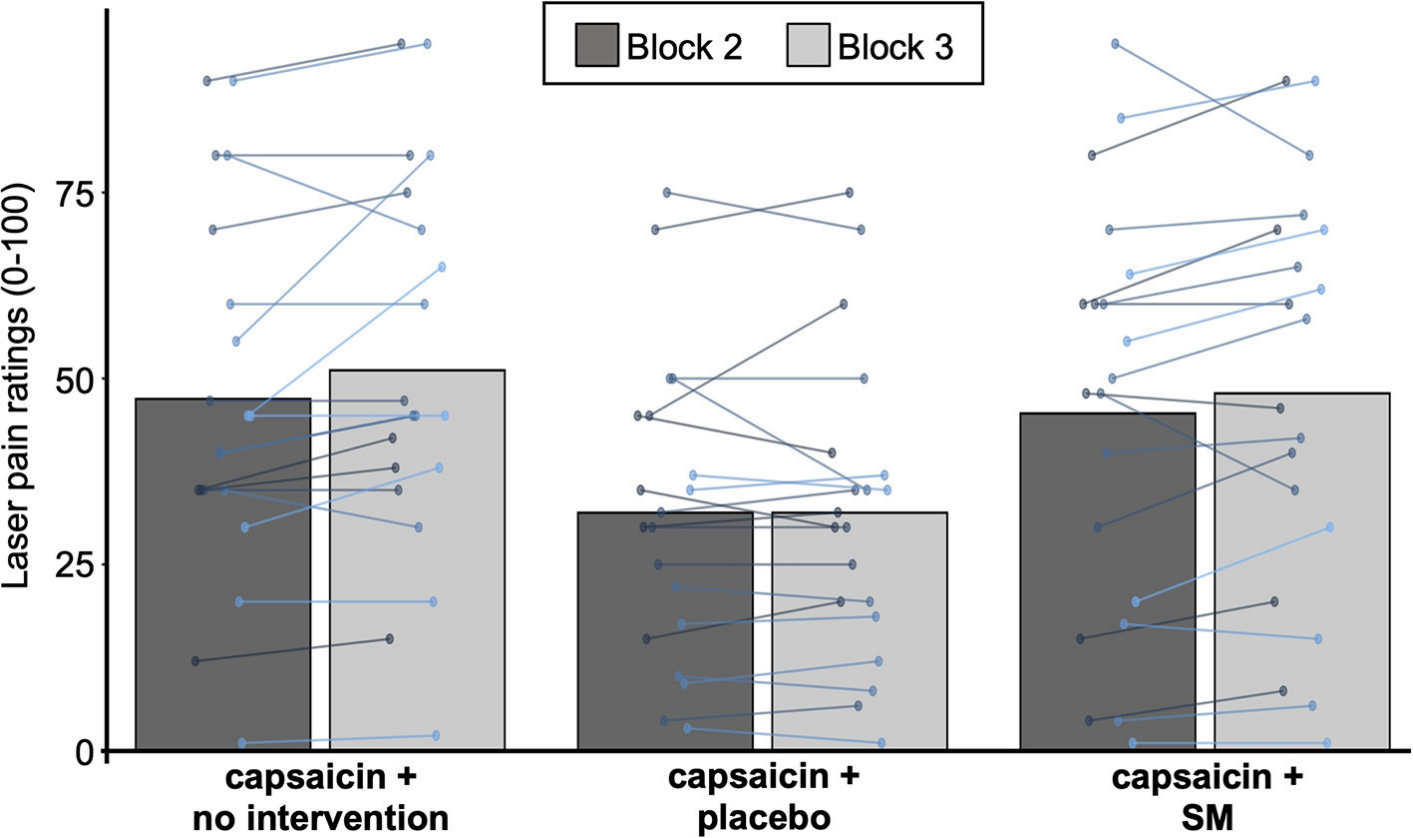
Effect of SM on heat pain amplification. Mean laser pain ratings for the three groups with capsaicin cream during block 2 and block 3 (before and after the intervention). Data from each participant are represented by linked colored points and the mean of these data points for each block is represented by gray bars. Note that the inert cream group did not report heat pain amplification and received no intervention, so it is not included in this analysis.

To examine the effects of interventions on heat pain amplification, changes in laser-pain relative to baseline were compared between the three groups with capsaicin cream (see Figure 5). Changes in laser-pain intensity significantly increased between blocks 2 and 3 (main effect: F_1,57_ = 6.17, *p* = 0.02, $\eta_{p}^{2}$ = 0.10). However, this effect was not significantly different between groups (interaction: F_2,57_ = 1.69, *p* = 0.19, $\eta_{p}^{2}$ = 0.06), indicating that heat pain amplification was not reduced by SM.

### Amplification of Aδ Laser-Evoked Potentials by Capsaicin

**Figure 6:**
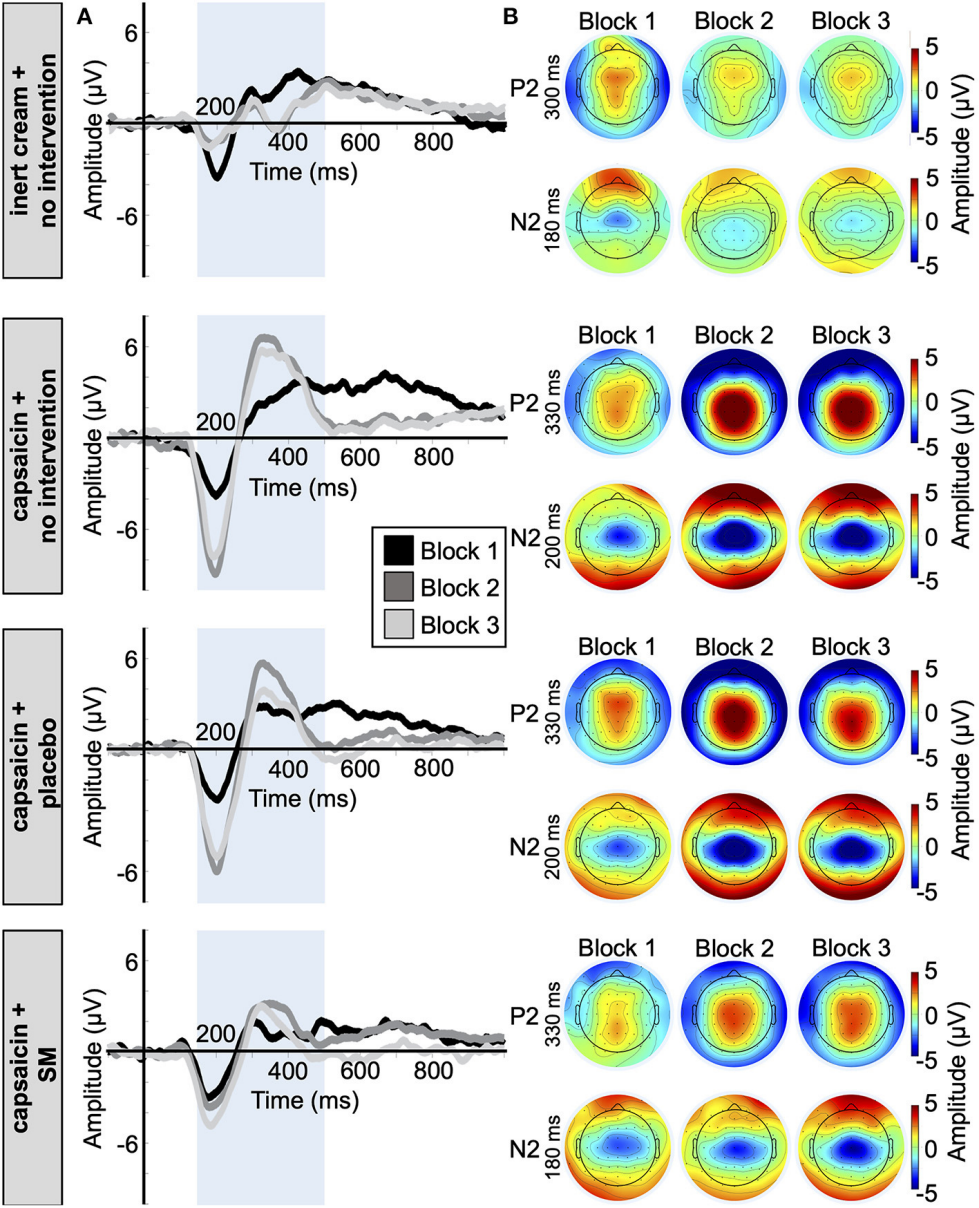
Aδ fiber laser-evoked potentials. **(A)** Average waveforms for the N2 and P2 at Cz with a nose reference of the 77 participants included in the Aδ fiber LEP analysis, time-locked to the onset of laser stimulation. **(B)** Average topographic maps for the Aδ-N2 and Aδ-P2 in the four groups.

Average waveforms and topographic maps for Aδ N2 and P2 components for all groups and conditions are presented in Figure 6. As expected, both components showed a central scalp distribution and were maximal at the vertex.

**Figure 7:**
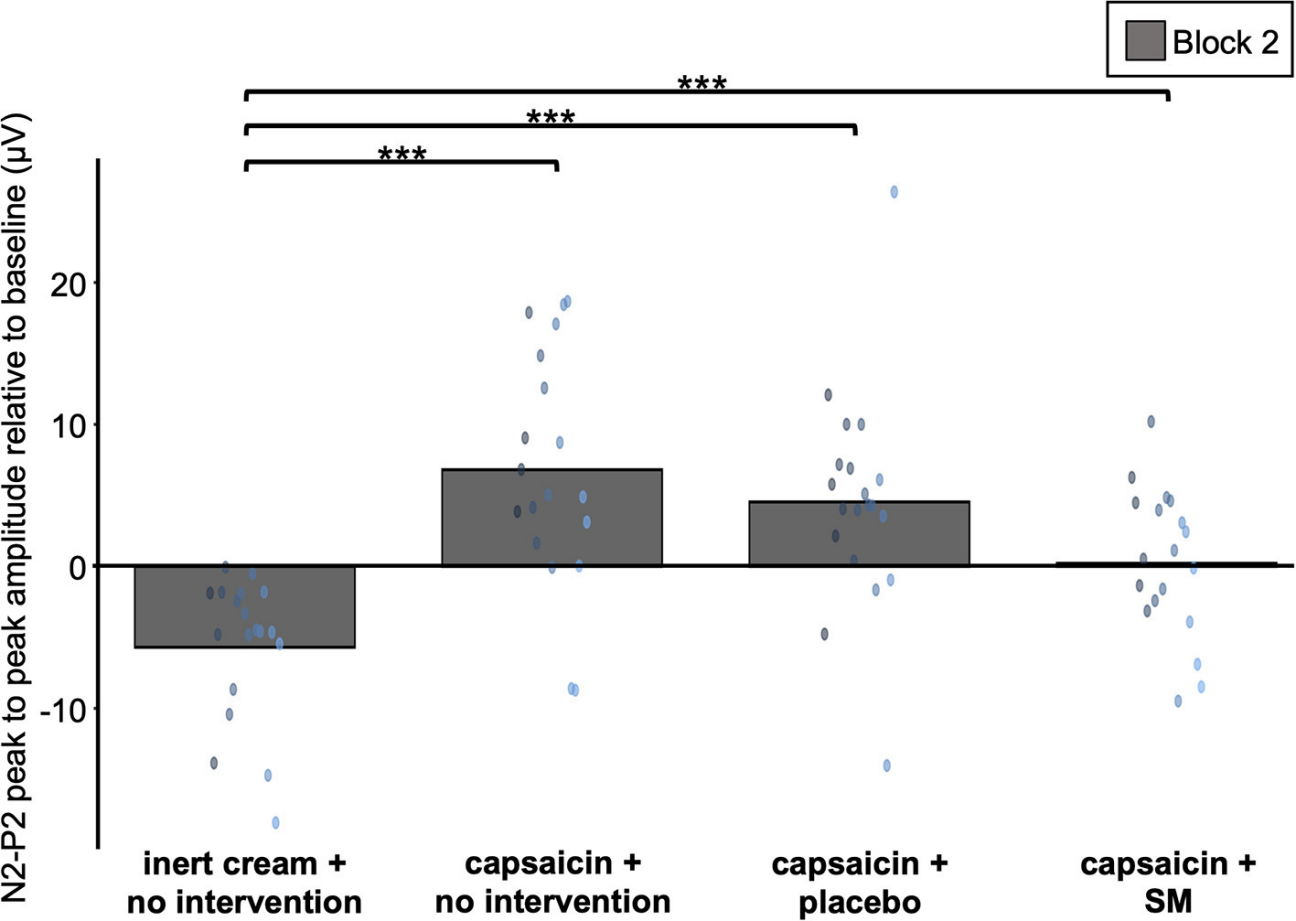
Amplification of Aδ fiber LEP by capsaicin. Average Aδ fiber N2-P2 peak to peak amplitude values during block 2 for the four experimental groups. These values are relative to baseline (block 2 minus block 1). Data from each participant are represented by colored points and the mean of these data points for each block is represented by gray bars. ***P < 0.001.

Aδ N2-P2 amplitudes and latencies are reported in Tables 2, 3. The effect of capsaicin on Aδ N2-P2 amplitude is presented in Figure 7. For the change from block 1 to block 2, Aδ N2-P2 amplitude was significantly different between groups (main effect: F_3,73_ = 12.7, *p* < 0.001, $\eta_{p}^{2}$ = 0.34). Bonferroni-corrected planned contrasts revealed that compared with the inert cream group, Aδ N2-P2 amplitude was significantly increased in the three groups with the capsaicin cream (all *p* < 0.001). Moreover, Aδ N2 peak latency was significantly different between groups from block 1 to block 2 (interaction: F_3,73_ = 6.3, *p* < 0.001, $\eta_{p}^{2}$ = 0.21). Bonferroni-corrected planned contrasts revealed that compared with the inert cream group, the Aδ N2 peak latency was shorter for block 2 compared with block 1 in the capsaicin with no intervention group (*p* < 0.001), in the placebo intervention groups (*p* < 0.001), but not in the SM group (*p* = 0.21). Similarly, Aδ P2 peak latency was significantly different between groups from block 1 to block 2 (interaction: F_3,73_ = 6.2, *p* < 0.001, $\eta_{p}^{2}$ = 0.20). Bonferroni-corrected planned contrasts revealed that the Aδ P2 peak latency was shorter at block 2 in the capsaicin with no intervention group (*p* = 0.006), in the placebo intervention group (*p* < 0.001), but not in the SM group (*p* = 0.78) when compared with the inert cream group.

**Table 2:** Aδ N2-P2 peak to peak amplitude (μV) for the four experimental groups (mean ± SD).

		**Inert cream**	**Capsaicin cream**		
		**No intervention**	**No intervention**	**Placebo**	**SM**
Aδ	Block 1	12.6 ± 3.5	13.2 ± 5.3	11.7 ± 4.9	10.9 ± 4.1
	Block 2	6.9 ± 1.7	20.0 ± 3.4	16.3 ± 3.4	11.2 ± 1.9
	Block 3	5.8 ± 2.2	17.5 ± 3.1	13.4 ± 3.3	11.5 ± 3.6

**Table 3:** N2 and P2 peak latency (ms) for the four experimental groups (mean ± SD).

			**Inert cream**	**Capsaicin cream**		
			**No intervention**	**No intervention**	**Placebo**	**SM**
Aδ	N2	Block 1	249.1 ± 42.3	252.4 ± 49.3	267.3 ± 53.4	227.7 ± 40.4
		Block 2	274.5 ± 31.3	204.4 ± 22.2	204.7 ± 27.9	212.1 ± 20.3
		Block 3	272.1 ± 46.5	203.3 ± 29.0	205.2 ± 26.8	211.5 ± 23.9
	P2	Block 1	405.4 ± 39.1	408.9 ± 52.2	421.6 ± 55.6	382.3 ± 51.2
		Block 2	419.2 ± 36.6	344.0 ± 31.4	347.3 ± 30.3	369.2 ± 23.9
		Block 3	380.8 ± 51.0	354.5 ± 26.2	352.7 ± 30.0	352.4 ± 40.5

Altogether, these results indicate that heat pain amplification by capsaicin was associated with increased Aδ N2-P2 amplitude and decreased Aδ N2 and P2 peak latencies.

### Effects of SM on the Amplification of Aδ Laser-Evoked Potentials by Capsaicin

**Figure 8:**
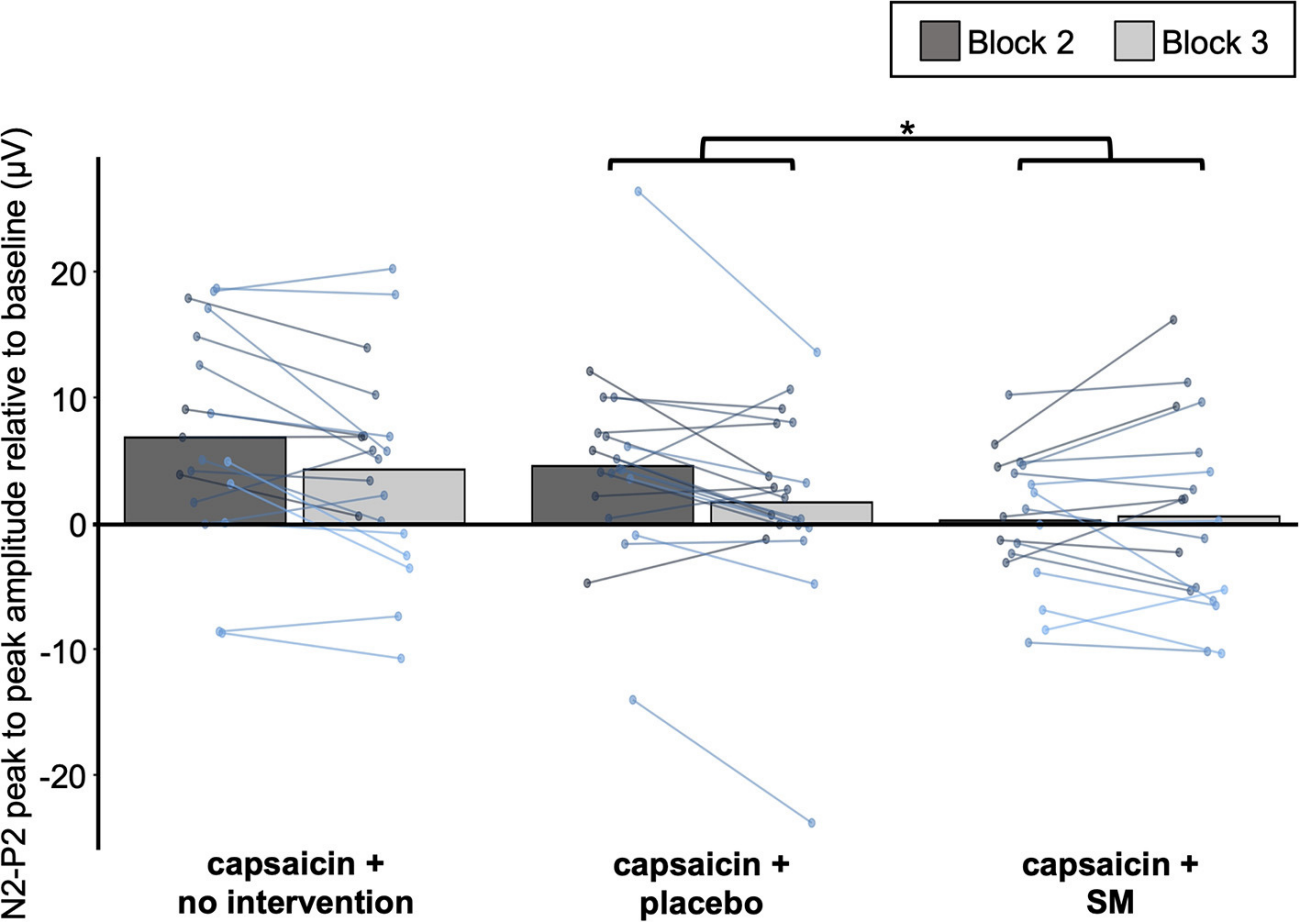
Effect of SM on Aδ fiber LEP. Average Aδ fiber N2-P2 peak to peak amplitude values for the three groups with capsaicin cream before (block 2) and after (block 3) the intervention. Data from each participant are represented by linked colored points and the mean of these data points for each block is represented by gray bars. *P < 0.05.

The effects of interventions on the amplification of Aδ-nociceptor activity by capsaicin were examined with the three capsaicin groups (see Figure 8). Aδ N2-P2 amplitude was significantly different between groups over time (interaction: F_2,55_ = 3.3, *p* = 0.043, $\eta_{p}^{2}$ = 0.11). Bonferroni-corrected planned contrasts revealed that the change between block 2 and block 3 was significantly different between the SM and placebo intervention groups (*p* = 0.043), where the SM group did not show a decrease in Aδ N2-P2 amplitude. However, no significant difference was observed between the SM and no intervention groups (*p* = 0.08).

Besides, Aδ N2 peak latency was not significantly different between groups over time (from block 2 to block 3) (interaction: F_2,55_ = 0.05, *p* = 0.95, $\eta_{p}^{2}$ = 0.002), but the effect was significant for the P2 latency (interaction: F_2,55_ = 4.1, *p* = 0.02, $\eta_{p}^{2}$ = 0.13). However, Bonferroni-corrected planned contrasts revealed that P2 peak latency in block 3 was not significantly different between the SM group compared with the placebo group (*p* = 0.06), although it was significantly different compared with no intervention group (*p* = 0.02).

### Expectations and Blinding

Expectations scores were 1.2 ± 32.4 for the SM group and −5.7 ± 16.6 for the placebo group. An independent *t*-test revealed no significant difference between groups [*t*_(38)_ = 0.83, *p* = 0.41; $\eta_{p}^{2}$ = 0.02]. In addition, expectations did not predict changes in capsaicin pain, laser pain or Aδ N2-P2 amplitude, induced by SM (all R^2^ < 0.08, all *p* > 0.2), or by the placebo (all R^2^ < 0.17, all *p* > 0.07). Regarding blinding, the number of participants reporting that they received an effective intervention was not significantly different between groups [SM: 9/20 (45%); placebo intervention: 7/20 (35%); $\chi_{\left(1\right)}^{2}$ = 0.36, *p* = 0.55]. Besides, expectations of pain relief were not significantly different between participants reporting that they received an effective vs. ineffective intervention for the SM group (Mann-Whitney *U* = 42.5, *p* = 0.62), for the placebo group (Mann-Whitney *U* = 32.0, *p* = 0.30) or both groups combined (Mann-Whitney *U* = 182.0, *p* = 0.79).

## Discussion

The aim of the present study was to investigate the effects of segmental chiropractic SM on heat pain amplification produced by capsaicin. The application of topical capsaicin in the back induced heat pain amplification and increased nociceptive brain activity associated with the activation of A-δ fibers. However, contrary to our hypothesis, SM did not reduce heat pain amplification and the associated amplification of nociceptive brain activity.

### Heat Pain Amplification Induced by Capsaicin

The tonic pain and heat pain amplification induced by topical capsaicin reported in the present study are consistent with the effects reported previously (37, 39, 64). Moreover, the increase in Aδ LEP amplitude is consistent with the effects of nociceptive laser stimuli on capsaicin treated skin (44, 65–67). By contrast, a decrease in Aδ LEP amplitude on capsaicin treated skin was reported in one study (68). However, this discrepancy is likely due to the lower concentration of capsaicin [0.1% instead of 0.6% (66) or 1% in the present study and in (44, 65, 67)]. It should be noted that the amplitude of A-δ LEPs in the SM group of the present study was less affected by capsaicin and this may have affected the results. However, capsaicin-evoked pain, laser-evoked pain, laser stimulation intensity, and expectation scores were not significantly different between groups. Therefore, these factors are unlikely to explain the smaller effect of capsaicin on A-δ LEPs in the SM group, which may reflect variability.

### Effects of Spinal Manipulation on the Amplification of Heat Pain and Nociceptive Brain Activity by Capsaicin

In the present study, the capsaicin-heat pain model was used for its ability mimic some aspects of pathological pain such as spontaneous pain and increased pain sensitivity (38), which are commonly associated with low back pain (69, 70). Indeed, topical capsaicin has been used in previous studies to induce experimental low back pain (71) and neck pain (72).

The present findings show that SM has no effect on this type of experimental back pain or on the increased sensitivity to painful laser stimuli in the present experimental conditions. This is consistent with results from a recent study in which segmental SM reduced secondary mechanical hyperalgesia induced by capsaicin but not capsaicin pain (30). However, this contrasts with results from a previous study in which capsaicin pain was reduced by SM (40). Nonetheless, several factors may explain these discrepancies.

Firstly, the application of a mechanical force such as SM on an acutely inflamed and sensitized area activates nociceptive afferents, which excitatory activity may counter centrally-mediated hypoalgesic effects. Accordingly, in the study that reported a reduction in capsaicin pain, topical capsaicin was applied to the forearm and SMs were not performed on sensitized tissues. This suggests that SM applied close to, but not directly on the acutely inflamed area may produce more favorable outcomes, but this remains to be investigated with a clinical sample. It should be emphasized that topical capsaicin and acute musculoskeletal injury do not sensitize the same tissues, and SM may produce different effects on capsaicin and clinical pain. In line with this idea, it has been shown in patients with chronic back pain that brain activity related to spontaneous clinical pain differs from the brain activity related to experimental pain evoked in their back (73). However, it should be noted that findings from several studies suggest that SM affects the transmission of spinal nociceptive activity, regardless of the origin of the inputs (cutaneous or myofascial) (23–30), although this remains to be confirmed with neurophysiological measures of spinal cord activity. Therefore, the lack of effect in the present study suggests that SM does not influence spinal nociceptive transmission when it is amplified by peripheral sensitization. Future studies should confirm these findings with other experimental pain models, including muscle pain induced by exercise or intramuscular hypertonic saline.

Secondly, although previous studies have demonstrated that a single SM can decrease experimental pain (23, 74), it is possible that a single SM is not sufficient to reduce peripheral sensitization and pain hypersensitivity. This may explain the discrepancy between the present results and a previous study in which capsaicin pain was reduced by multiple SMs during one session (40). Although the clinical relevance of the present results is limited, it is worth noting that in clinical studies, the number of SM is associated with outcomes. For example, a randomized controlled trial on the dose-response effects of SM for chronic low back pain in which participants received either 0, 6, 12 or 18 sessions of multiple SMs reported that 12 sessions yielded the most favorable improvements in pain level and disability (75). In addition, another study from the same group investigating the effect of the same doses of SM in participants with chronic cervicogenic headache reported a linear dose-response relationship between the number of SM sessions and the number of days per month with cervicogenic headache, the most effective dose being 18 sessions (76).

Altogether, the findings from the present and previous studies suggest that centrally mediated neurophysiological mechanisms independent of primary hyperalgesia may produce the immediate pain reduction by SM, which may add to anti-inflammatory and other effects later on, in line with the current literature (23–26, 77–80). However, this should be confirmed with clinical populations since the effects of SM may be different on primary hyperalgesia associated with clinical pain. In addition, future studies may lead to different results if the modulation of primary hyperalgesia by SM is examined with mechanical stimuli instead of heat stimuli. It should also be emphasized that SM was applied only once and it remains to be determined whether several SM applied on different segments surrounding the hyperalgesic area may be more effective.

Although previous studies have examined brain activity following SM, no study investigated the effects of brain activity associated with capsaicin, which was used in the present study as an experimental model of tonic pain associated with pain hypersensitivity. The present results show that SM did not modulate LEP amplification by capsaicin, in line with the behavioral results. In two previous EEG studies in healthy volunteers, laser pain (81) and secondary hyperalgesia induced by capsaicin (30) were decreased by SM. However, these studies did not examine if primary hyperalgesia was modulated by SM. Reducing primary hyperalgesia may prevent or reduce the development of secondary hyperalgesia, but the present results indicate that the reduction of secondary hyperalgesia induced by capsaicin (30) cannot be explained by this mechanism. In another study in patients with subclinical pain, pain intensity and cerebral oscillations (1–32 Hz) induced by 80 s of tonic pain (hand cold-pressor test) were not modulated by SM applied on different regions of the spine (82). In functional magnetic resonance imaging (fMRI) studies, a decrease of pain-related activity was observed following a single SM, in healthy individuals (83, 84) and patients with neck pain (85). Other fMRI studies also suggest that SM may reduce chronic low back pain by modulating the saliency network activity or connectivity (86, 87). Thus, far, these conflicting findings highlight the need for further research to examine brain activity with protocols in which SM produces clear pain inhibition. Moreover, it should be emphasized that the present study and our previous reports (30, 81) were designed to examine brain activity evoked by the selective activation of A-δ and C fibers, and its modulation by segmental SM, with the hypothesis that segmental inhibition of nociceptive activity in the spinal cord would be reflected in decreased nociceptive activity in the brain. This does not exclude that cerebral processes non-specific to SM may also modulate nociceptive activity. Accordingly, future studies should include all appropriate controls to examine the effects of SM on pain perception and pain-related processes with well-supported hypothesis-driven experimental designs and a mechanistic approach, as proposed previously (80, 88).

### Expectations and Placebo Effects

Expectations did not predict any inter-individual differences in the variables of interest. Although some participants expected a large pain relief by the intervention, this did not lead to better outcomes, either in the SM group or the placebo group. Consistent with these results, less than half of participants reported the intervention as effective, regardless of the intervention group. The fact that participants were not aware of different group allocations and interventions may contribute to making expectations and placebo effects more comparable between groups. This may be examined and considered in future studies.

It has been shown that the nocebo effect increases pain ratings and LEPs (89). Capsaicin application may have induced such nocebo effects, competing with and hiding the potential inhibition of Aδ LEP and pain by SM. A measure of expectations relative to capsaicin in addition to a measure of expectations related to the effects of SM and placebo could clarify this issue in a future study.

### Significance

There are several important steps between basic research and clinical practice guidelines, and the objective of this basic study is to provide information on the underlying mechanisms of SM-induced inhibition of pain amplification due to peripheral sensitization. This is essential to feed applied and clinical research, and eventually, to provide a mechanistic rationale to support clinical practice guidelines. Accordingly, experimental pain models are validated in preclinical research on biomarkers, which may be measured in clinical studies, thereby narrowing the gap between basic and clinical findings (38). Recently, three reviews on the mechanisms of SM (80, 90) or manual therapy (91) stated the need for more well-designed mechanistic studies. The present study adds to previous basic studies to guide investigators on which measures should be used in mechanistic clinical trials and which mechanisms may underlie the clinical effects of SM in these clinical trials. Nevertheless, clinical recommendations should be considered cautiously when inferred from experimental pain models that may not completely reflect pathological mechanisms.

## Conclusion

In summary, the present results indicate that SM applied segmentally does not reduce the capsaicin-induced amplification of heat pain and the associated nociceptive brain activity. Future studies are needed to examine the modulation of primary heat and mechanical hyperalgesia in patients with chronic spine pain, in whom pathological changes in the dorsal horn of the spinal cord may lead to different results.

## Data Availability Statement

The raw data supporting the conclusions of this article will be made available by the authors, without undue reservation.

## Ethics Statement

The studies involving human participants were reviewed and approved by Comité d'éthique de la recherche chez l'être humain (UQTR). The patients/participants provided their written informed consent to participate in this study.

## Author Contributions

BP contributed to the study design, data collection, analysis and interpretation, and wrote the preliminary version of the manuscript. SN contributed to data collection and analyses. MP contributed to the study design, data collection, analyses, interpretation, wrote the final version of the manuscript, and obtained funding for the study. Each author has contributed significantly to this work and has read and approved the final version of the manuscript.

## Funding

This project was funded by the Fondation Chiropratique du Québec, the Natural Science and Engineering Research Council of Canada (#06659), and the Canadian Foundation for Innovation (#33731). The contribution of Benjamin Provencher was supported by the Fonds de Recherche du Québec – Santé (FRQS) and the Fondation Chiropratique du Québec. The contribution of Stéphane Northon was supported by the Fonds de Recherche du Québec – Nature et Technologie (FRQNT). The contribution of Mathieu Piché was supported by the Fonds de Recherche du Québec – Santé (FRQS).

## Conflict of Interest

The authors declare that the research was conducted in the absence of any commercial or financial relationships that could be construed as a potential conflict of interest.

## Publisher's Note

All claims expressed in this article are solely those of the authors and do not necessarily represent those of their affiliated organizations, or those of the publisher, the editors and the reviewers. Any product that may be evaluated in this article, or claim that may be made by its manufacturer, is not guaranteed or endorsed by the publisher.
