# Supplementary material for: Segmental Chiropractic Spinal Manipulation Does not Reduce Pain Amplification and the Associated Pain-Related Brain Activity in a Capsaicin-Heat Pain Model
Source: Front Pain Res (Lausanne). 2021 Nov 1;2:733727. doi: 10.3389/fpain.2021.733727 (PMC8915690; doi:10.3389/fpain.2021.733727)
Supplement: Supplementary file 1 [file Data_Sheet_1.PDF]

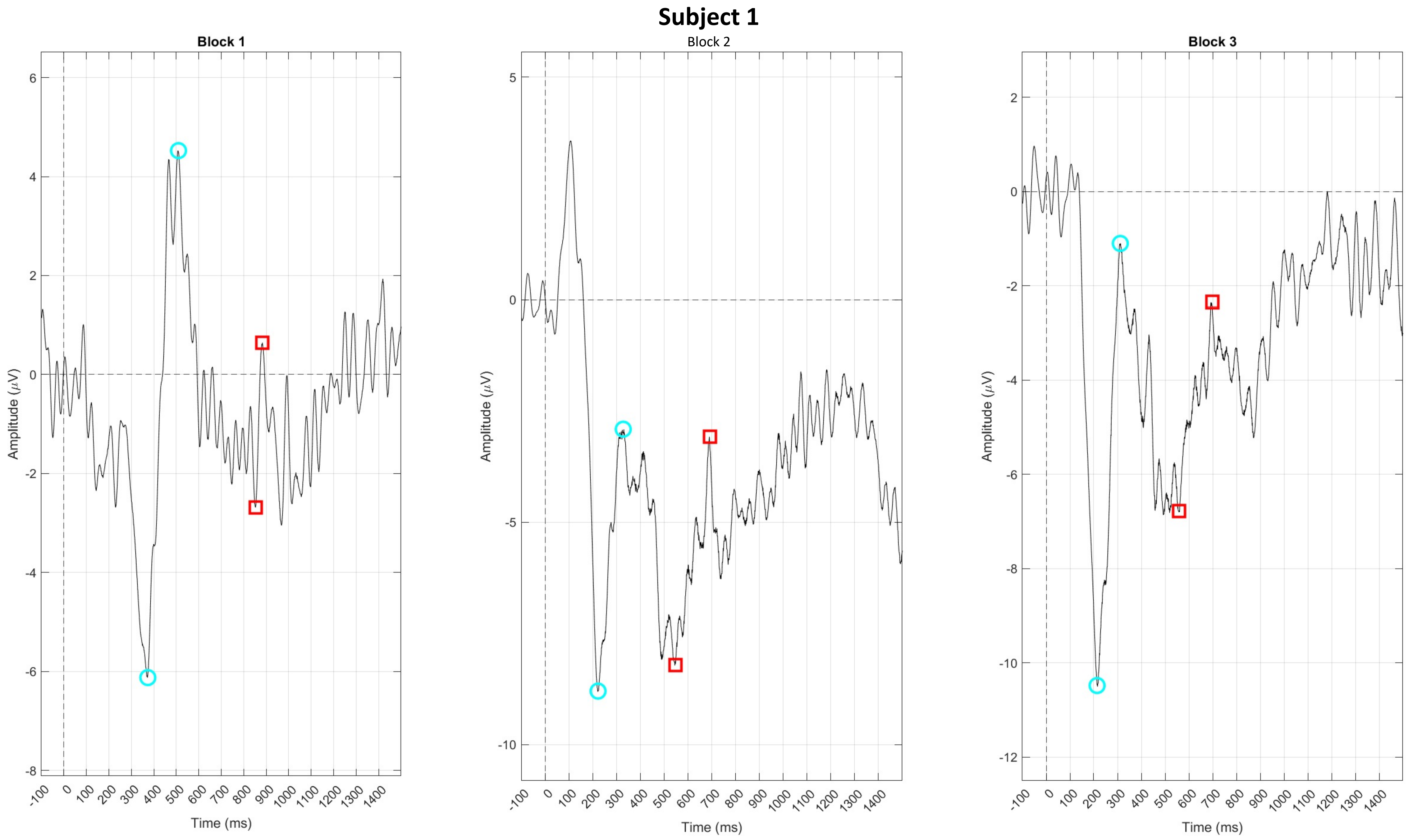

Subject 1
Block 1
Block 2
Block 3
Amplitude (μV)
Time (ms)
-100
0
100
200
300
400
500
600
700
800
900
1000
1100
1200
1300
1400
6
4
2
0
-2
-4
-6
-8
5
-5
-10
-12

Subject 2

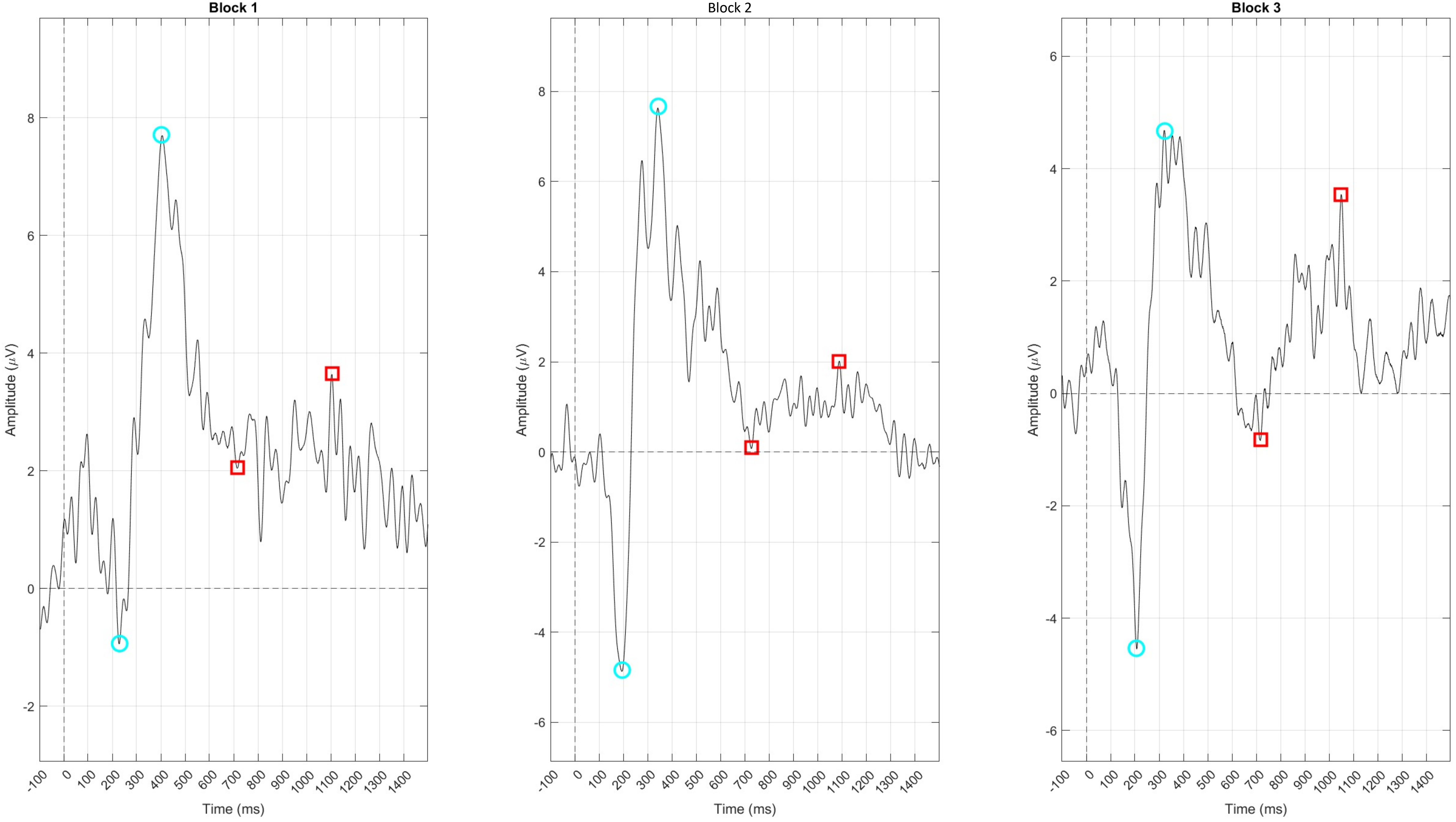

Block 1
Block 2
Block 3
Amplitude (μV)
Time (ms)
8
6
4
2
0
-2
-4
-6
-100
0
100
200
300
400
500
600
700
800
900
1000
1100
1200
1300
1400

Subject 3

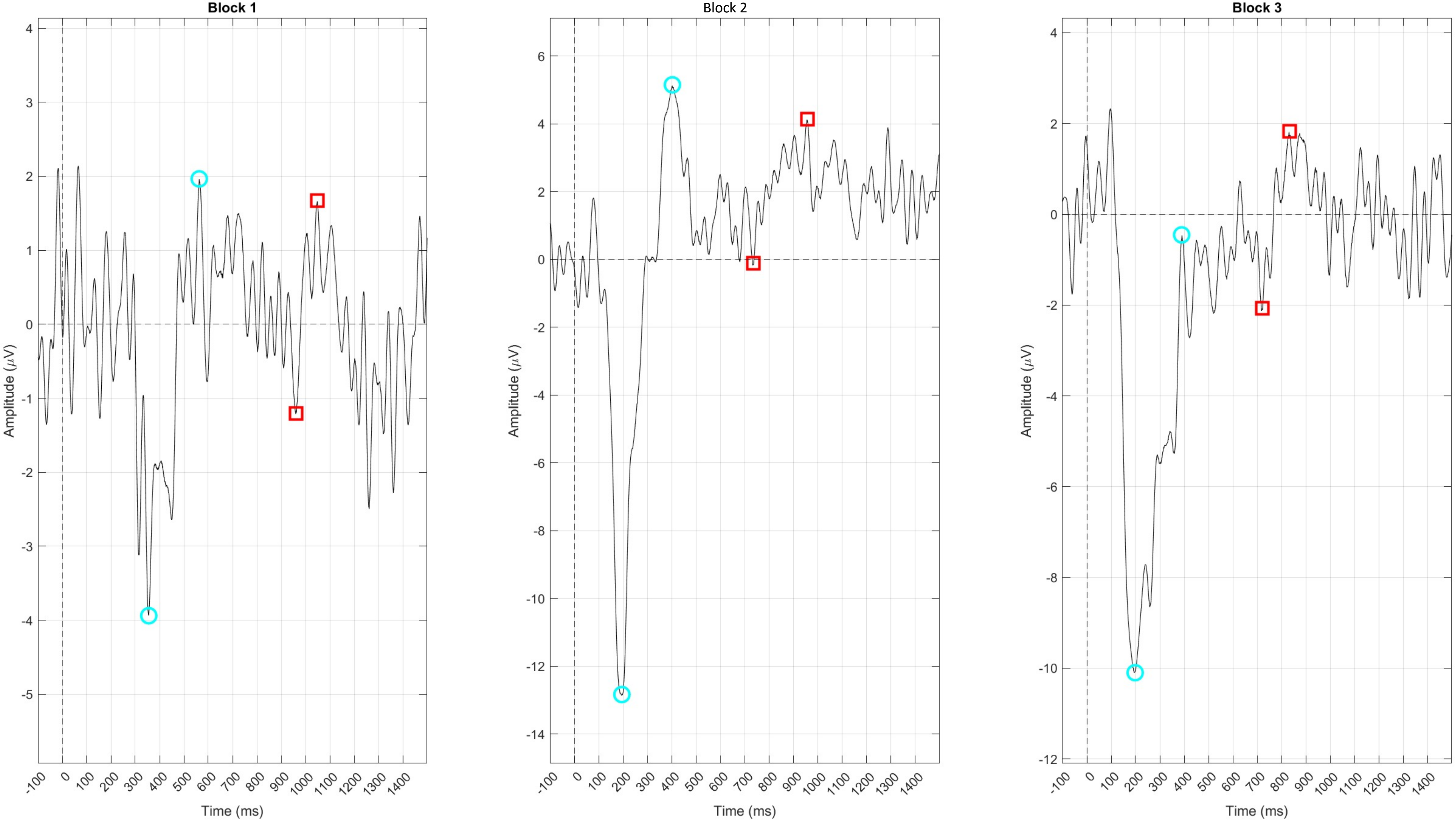

Subject 4

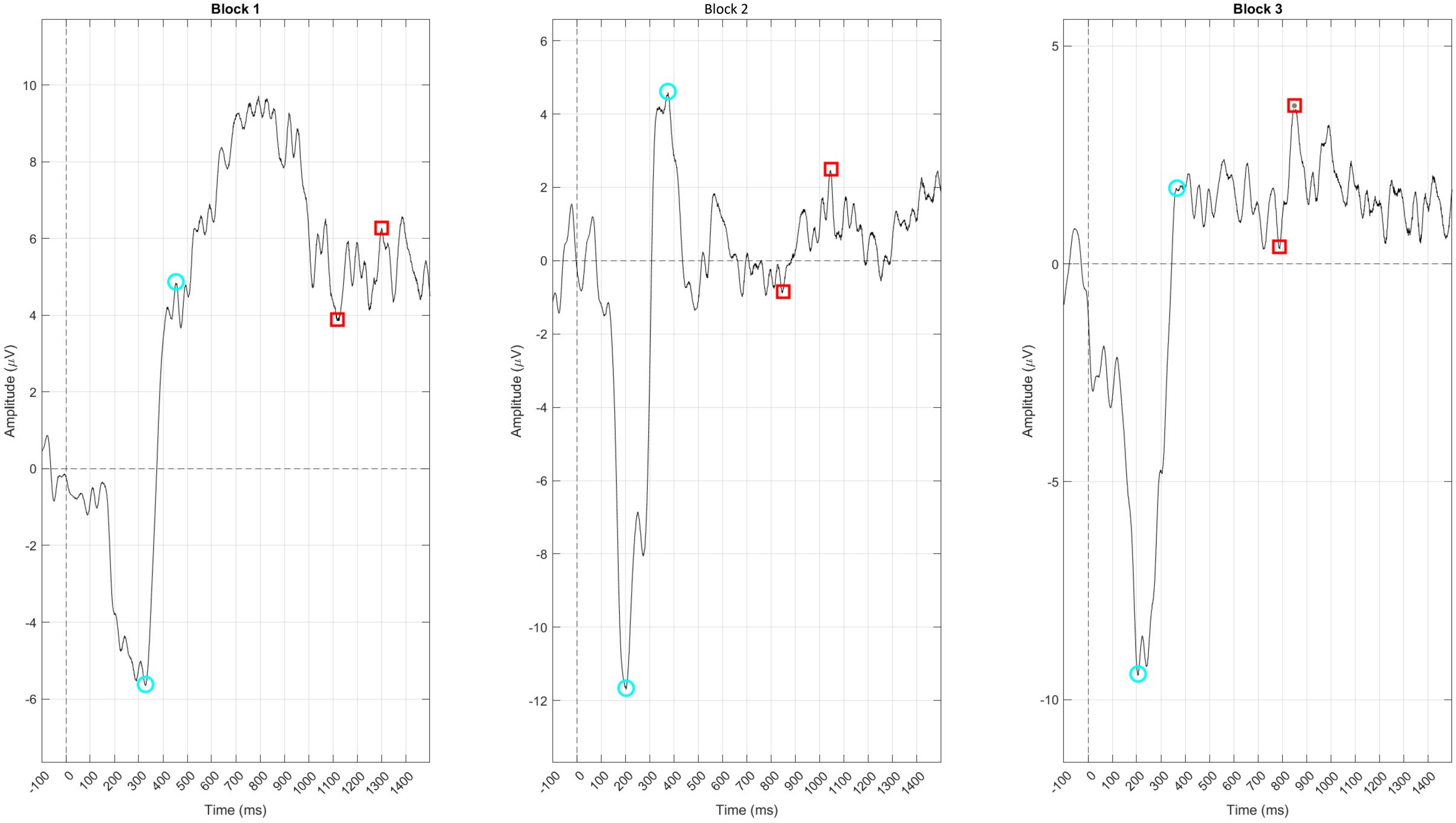
Block 1
Block 2
Block 3
Amplitude (μV)
Time (ms)
-100
0
100
200
300
400
500
600
700
800
900
1000
1100
1200
1300
1400

Subject 5

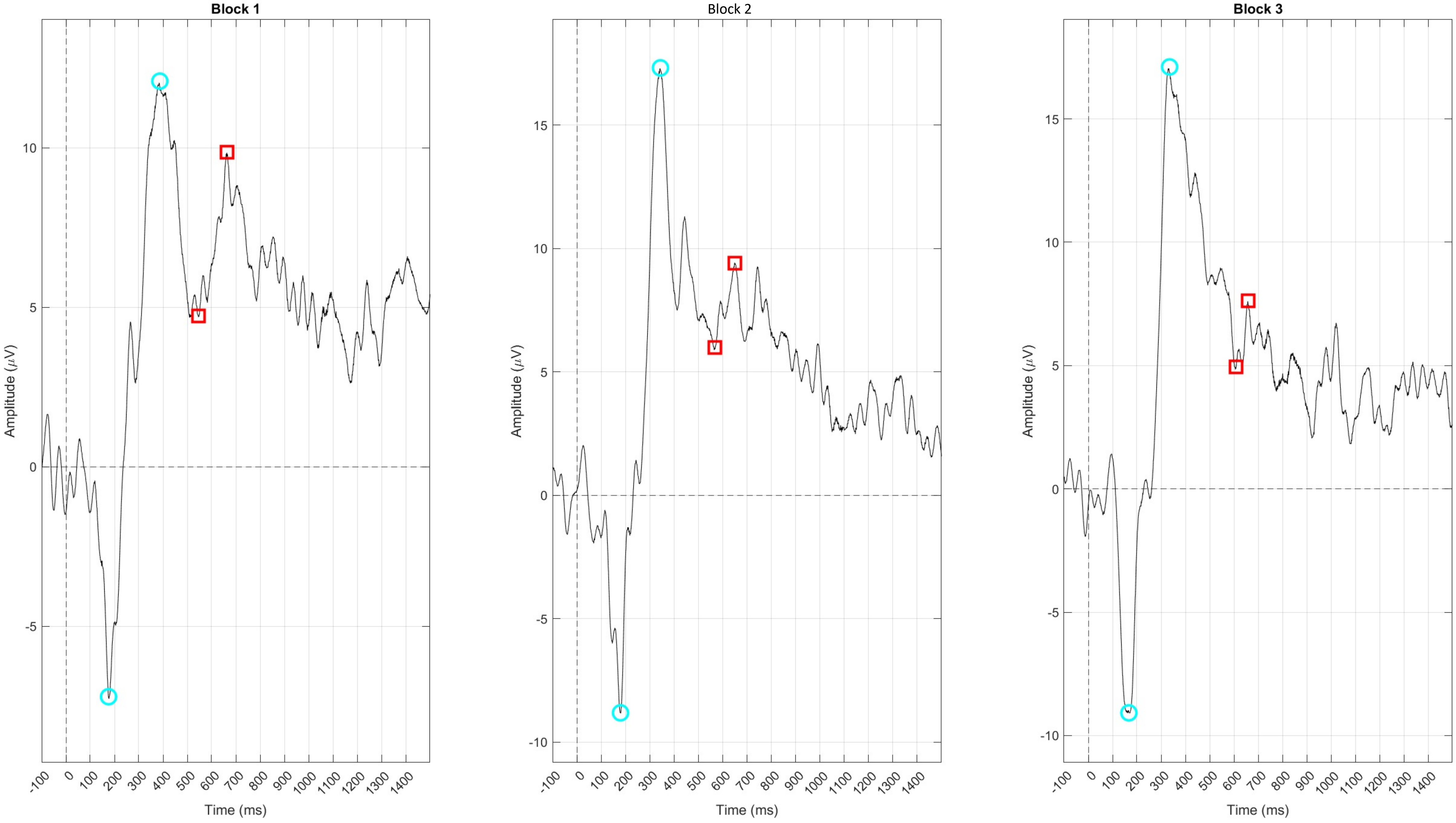

Block 1
Block 2
Block 3
Amplitude (μV)
Time (ms)
-100
0
100
200
300
400
500
600
700
800
900
1000
1100
1200
1300
1400

Subject 6

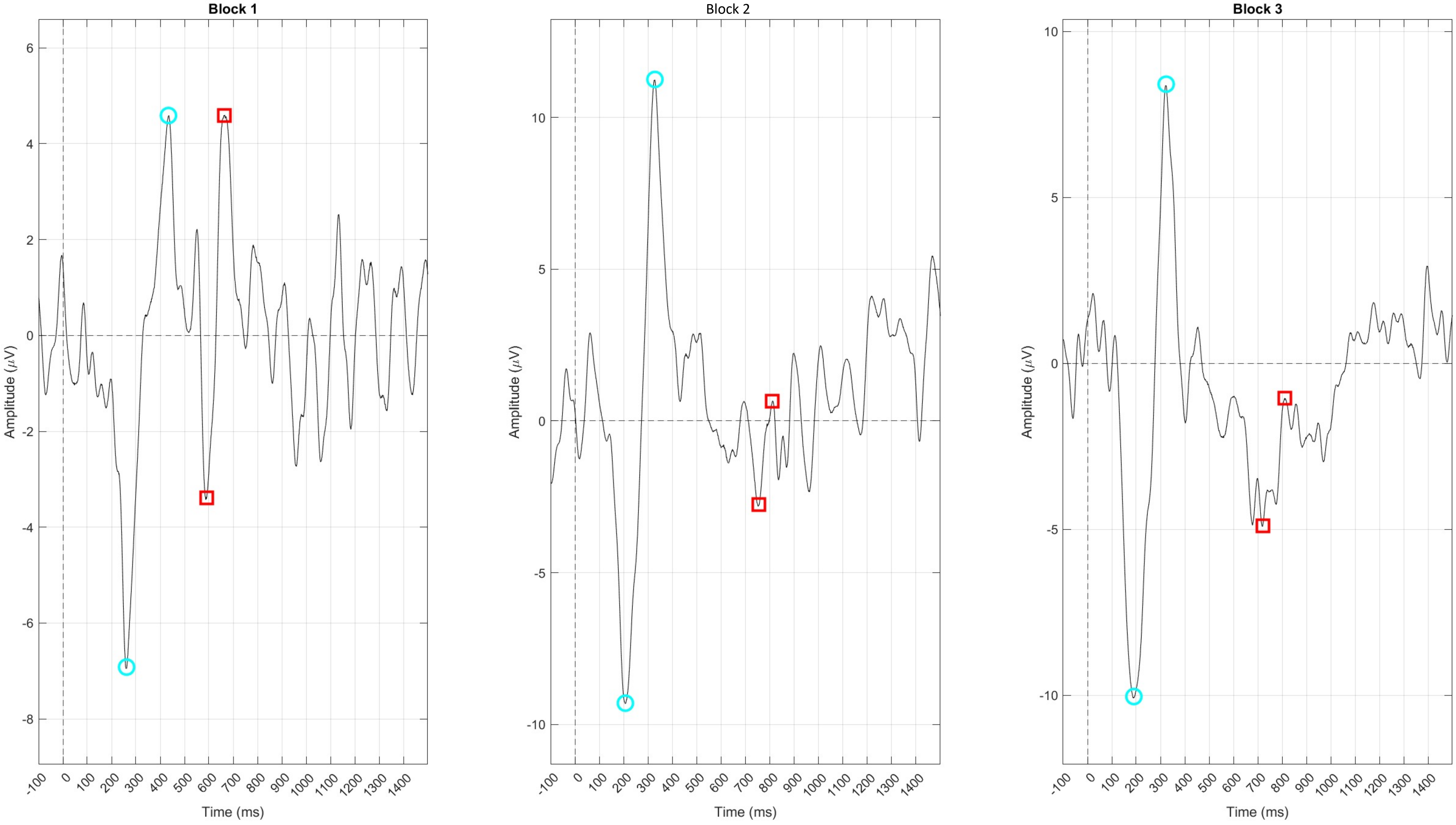
Block 1
Block 2
Block 3
Amplitude (μV)
Time (ms)
-100
0
100
200
300
400
500
600
700
800
900
1000
1100
1200
1300
1400
6
4
2
0
-2
-4
-6
-8
10
5
-5
-10

Subject 7

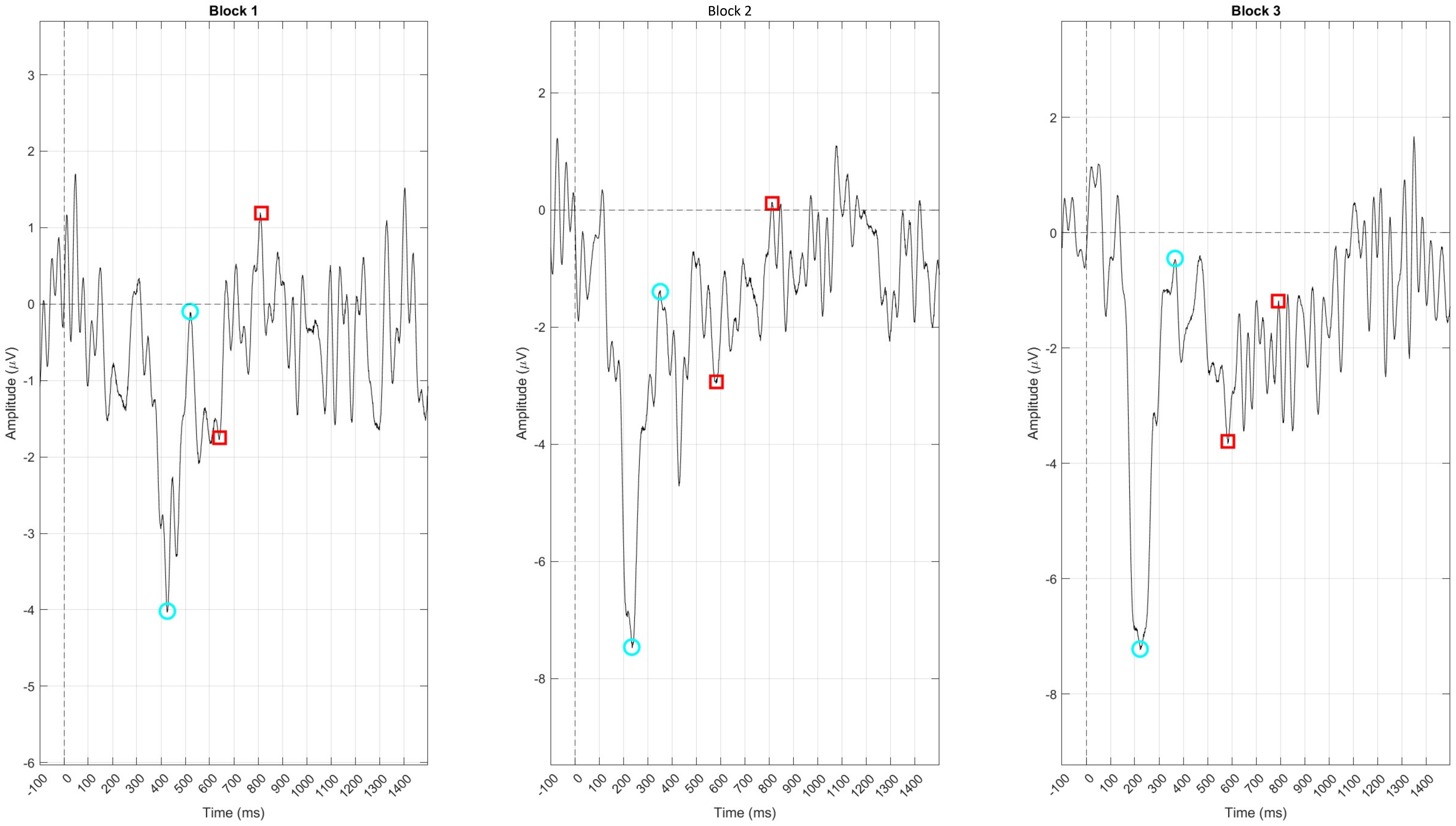
Block 1
Block 2
Block 3
Amplitude (μV)
Time (ms)
-100
0
100
200
300
400
500
600
700
800
900
1000
1100
1200
1300
1400

Subject 8

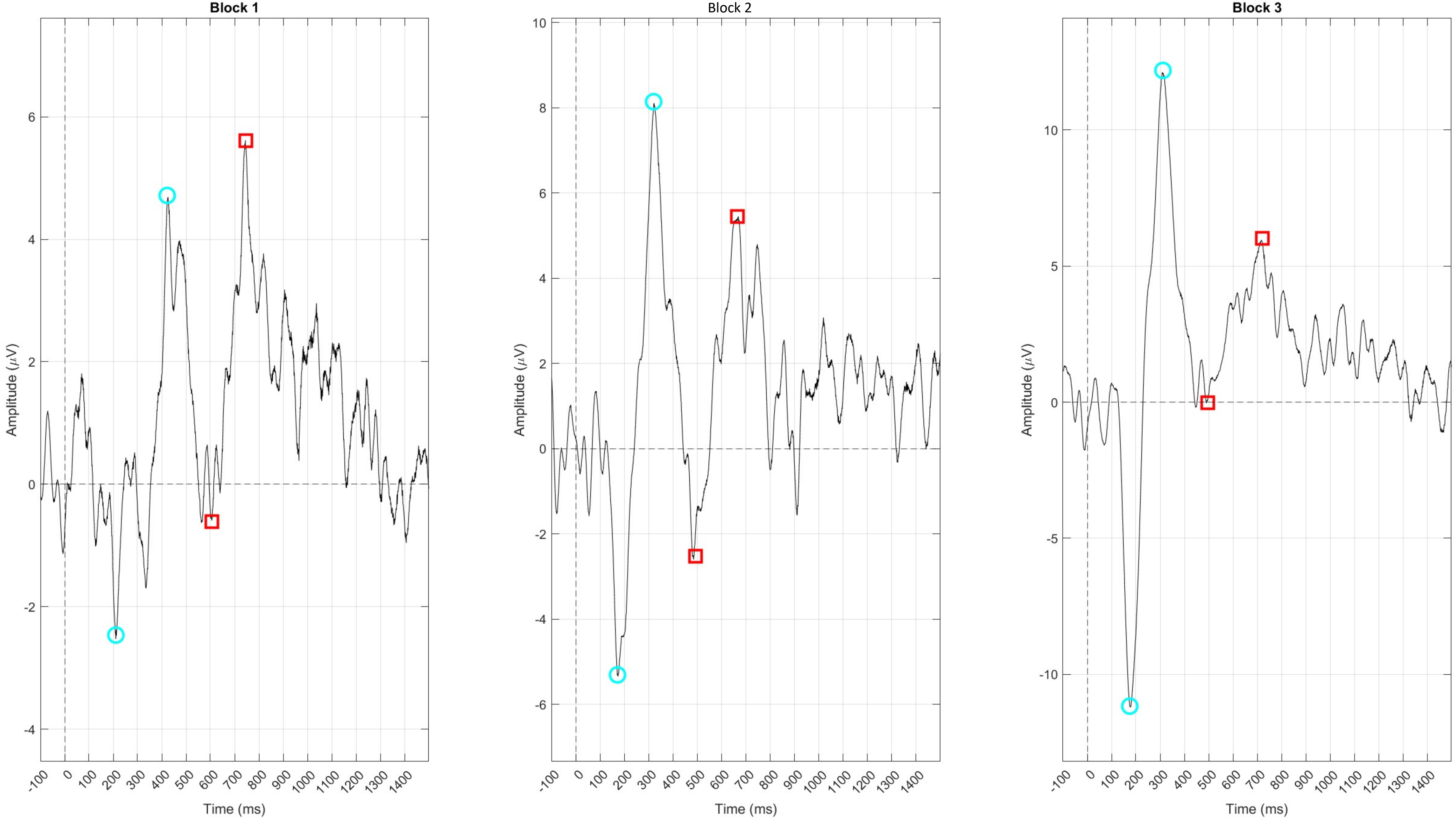

Block 1
Block 2
Block 3
Amplitude (μV)
Time (ms)
-100
0
100
200
300
400
500
600
700
800
900
1000
1100
1200
1300
1400
6
4
2
0
-2
-4
10
8
-6
5
-5
-10

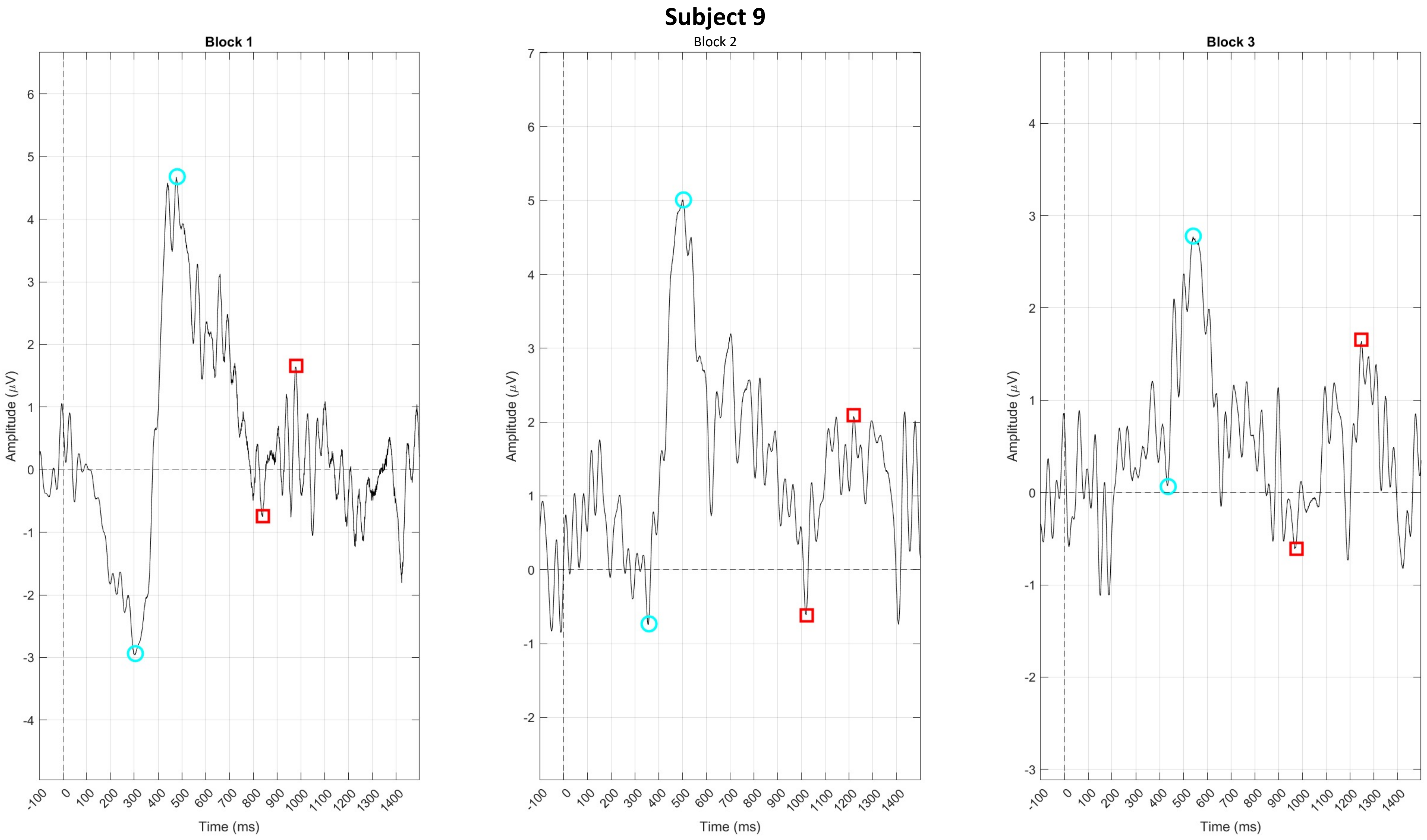
Subject 9
Block 1
Block 2
Block 3
Amplitude (μV)
Time (ms)

Subject 10

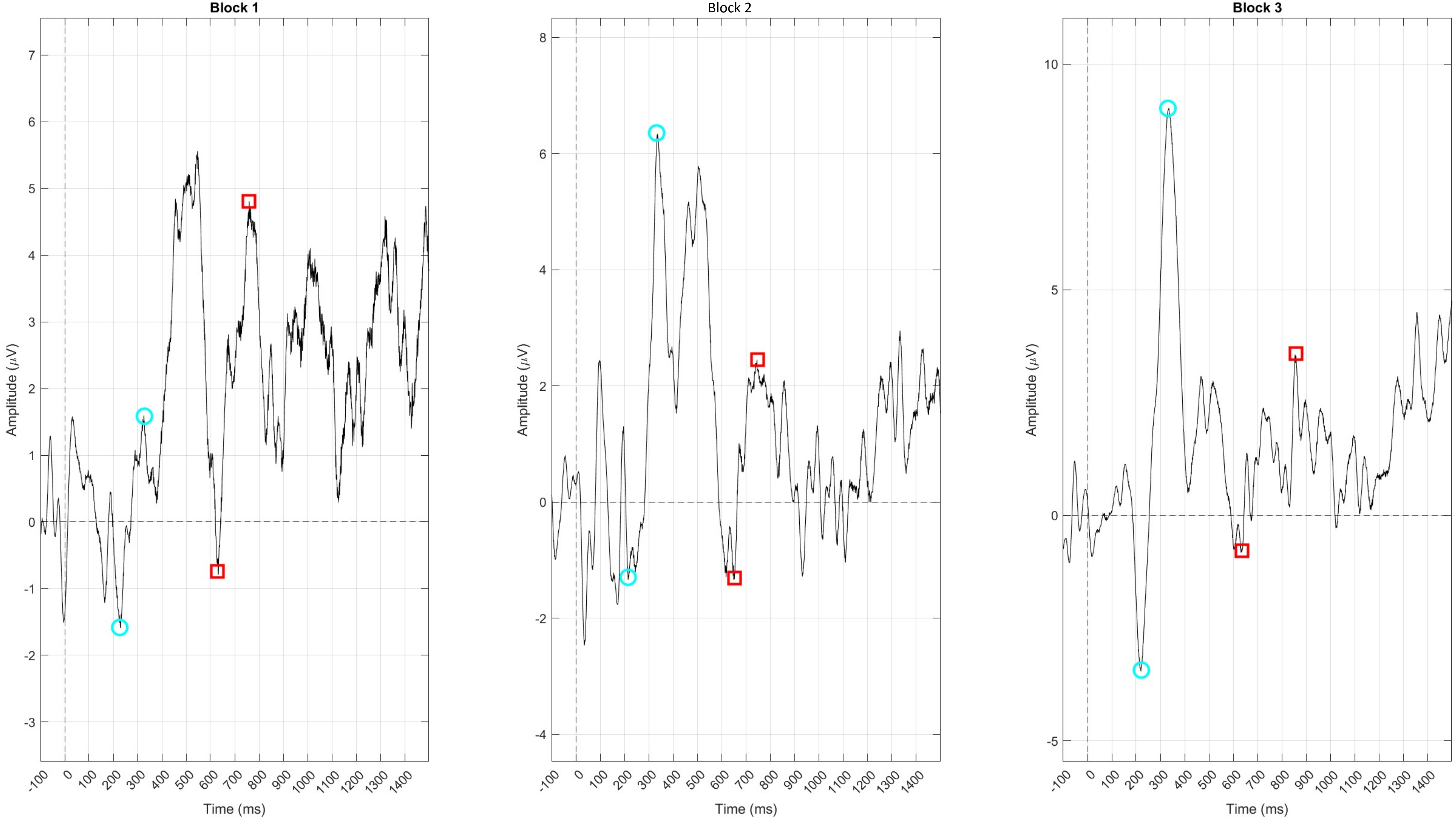
Block 1
Block 2
Block 3
Amplitude (μV)
Time (ms)
-100
0
100
200
300
400
500
600
700
800
900
1000
1100
1200
1300
1400

Subject 11 was excluded from analysis

Subject 12

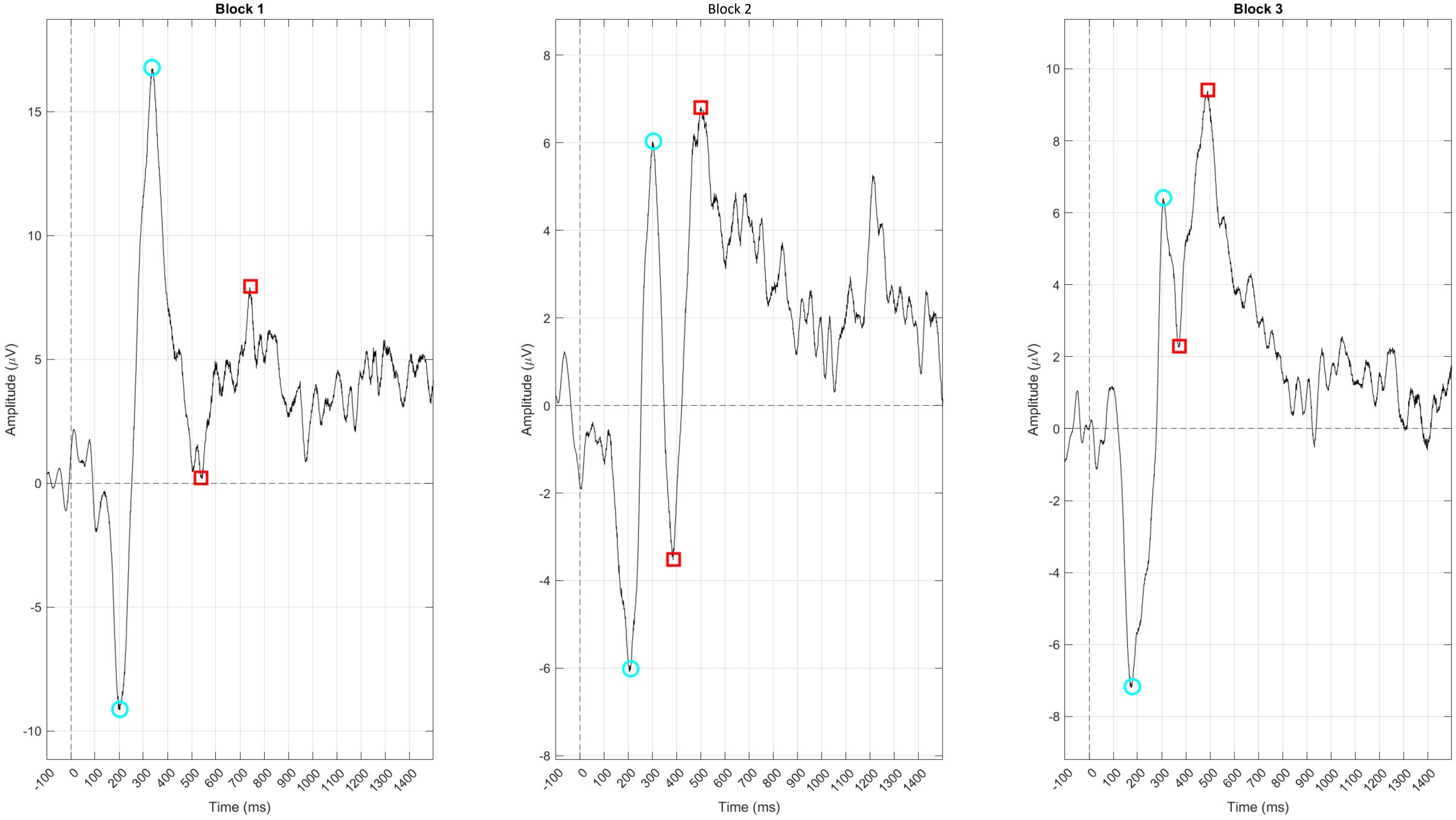
Block 1
Block 2
Block 3
Amplitude (μV)
Time (ms)
-100
0
100
200
300
400
500
600
700
800
900
1000
1100
1200
1300
1400
15
10
5
0
-5
-10
8
6
4
2
-2
-4
-6
-8

Subject 13

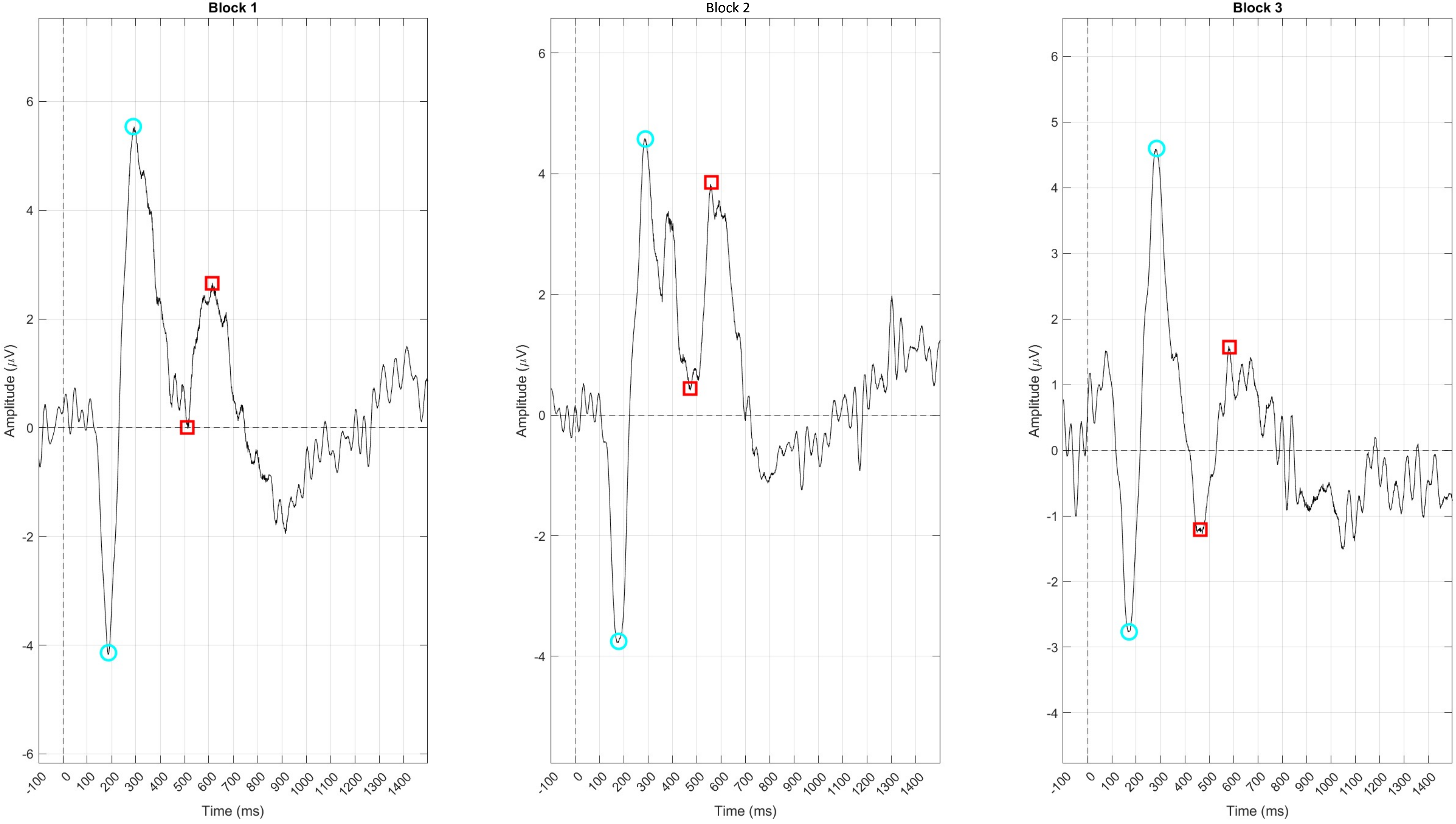

Subject 14

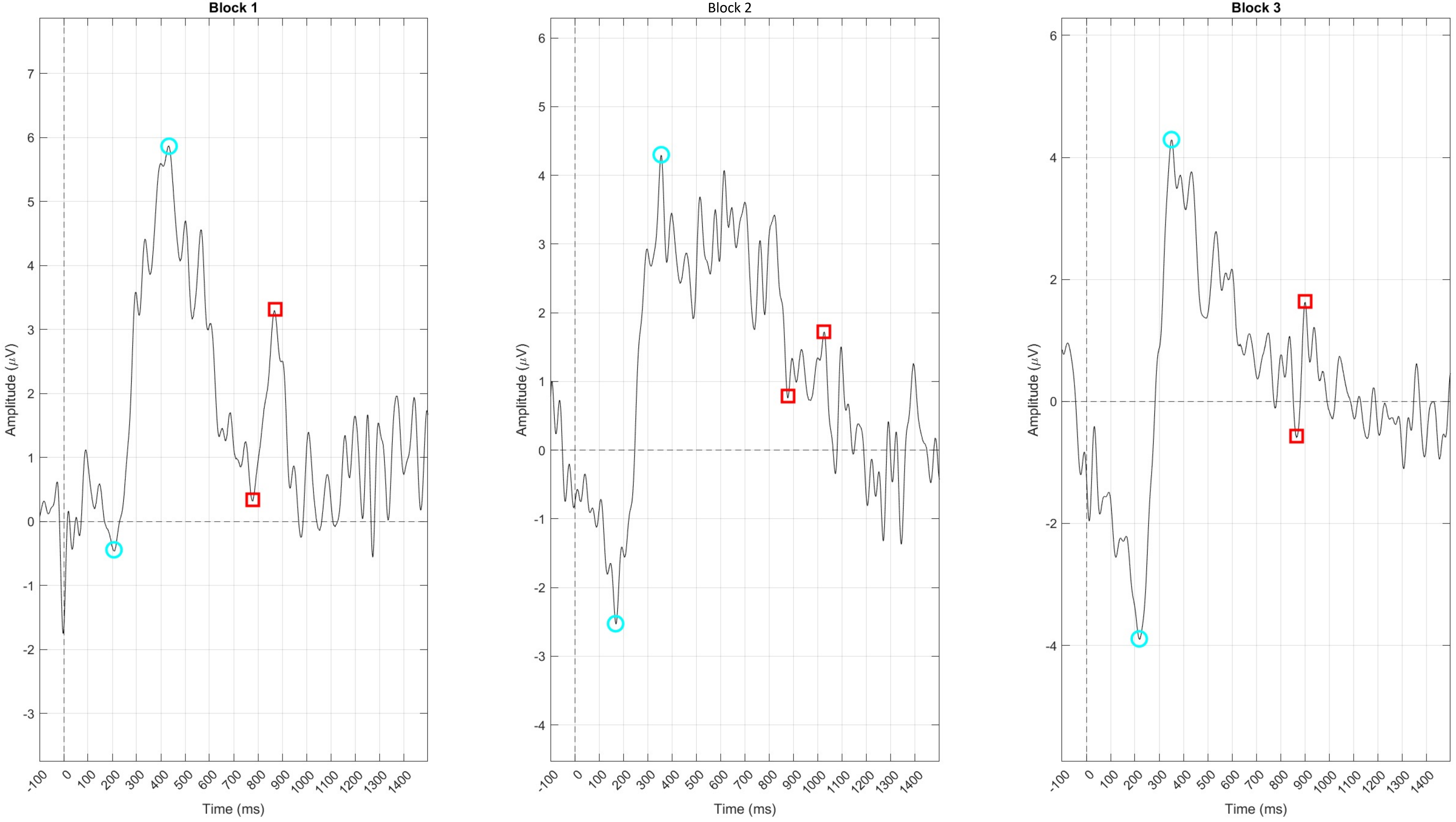

Block 1
Block 2
Block 3
Amplitude (μV)
Time (ms)
-100
0
100
200
300
400
500
600
700
800
900
1000
1100
1200
1300
1400

Subject 15

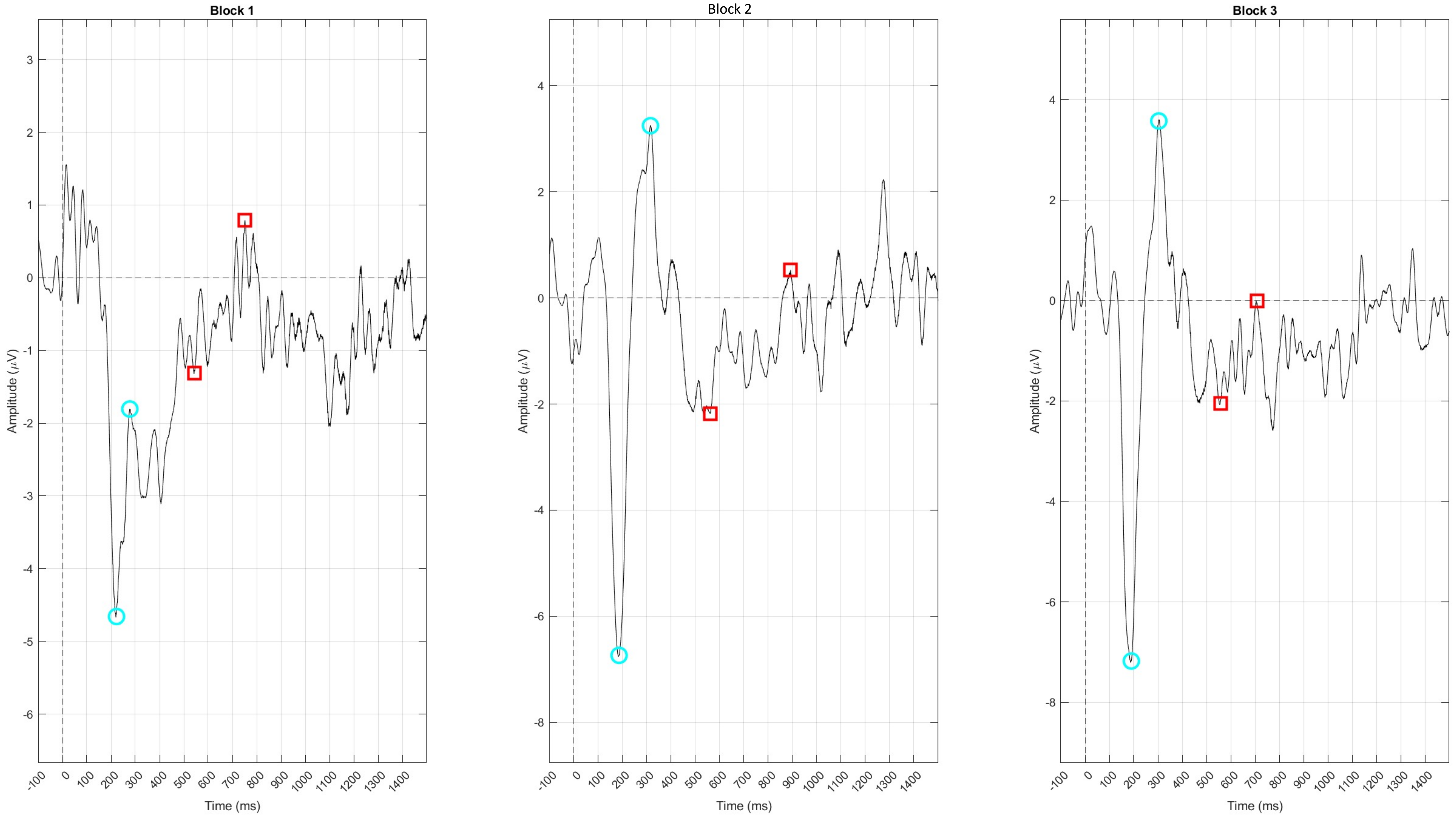
Block 1
Block 2
Block 3
Amplitude (μV)
Time (ms)
-100
0
100
200
300
400
500
600
700
800
900
1000
1100
1200
1300
1400
3
2
1
0
-1
-2
-3
-4
-5
-6
4
-8

Subject 16

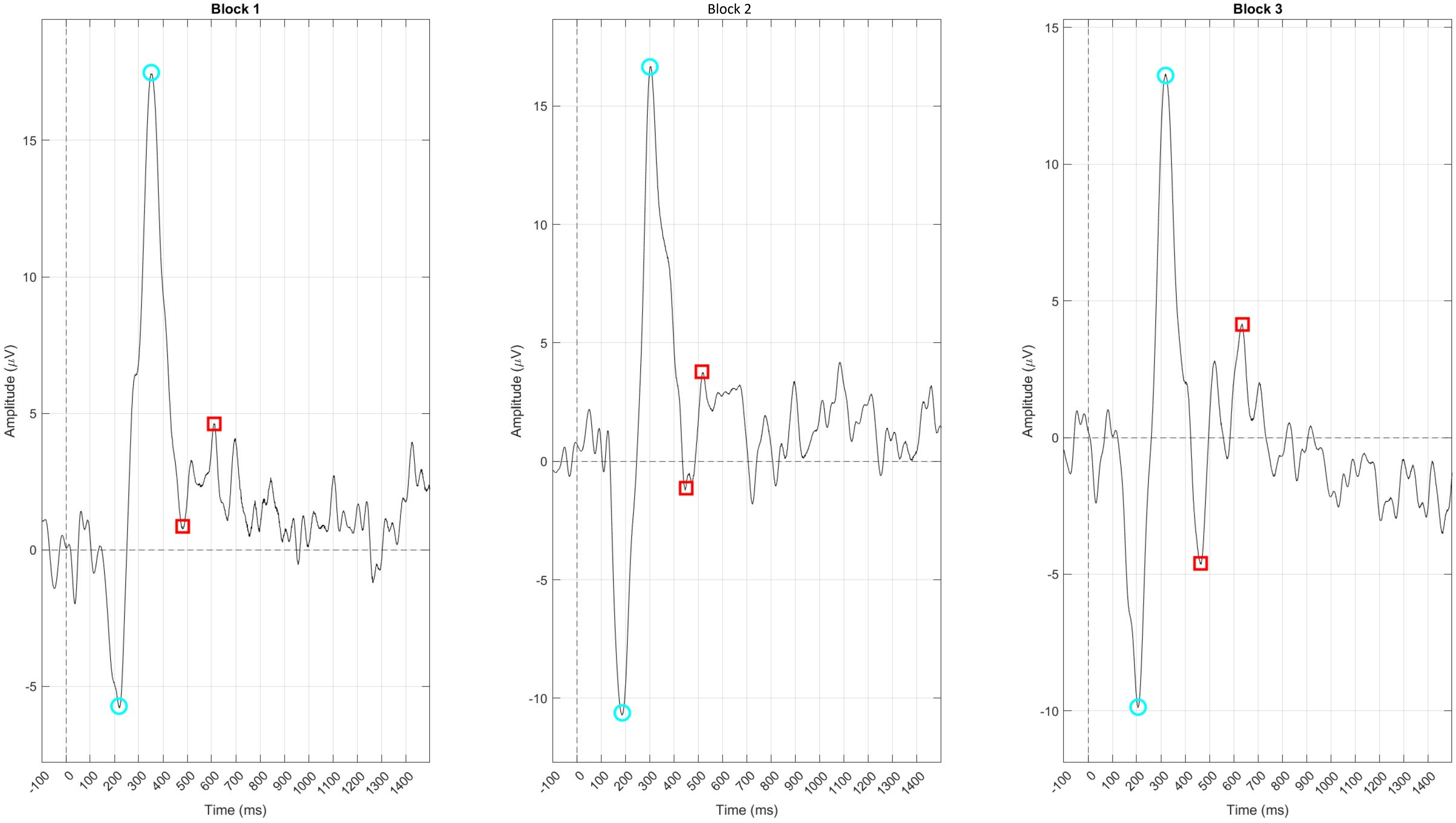

Block 1
Block 2
Block 3
Amplitude (μV)
Time (ms)
-100
0
100
200
300
400
500
600
700
800
900
1000
1100
1200
1300
1400
15
10
5
0
-5
-10

Subject 17

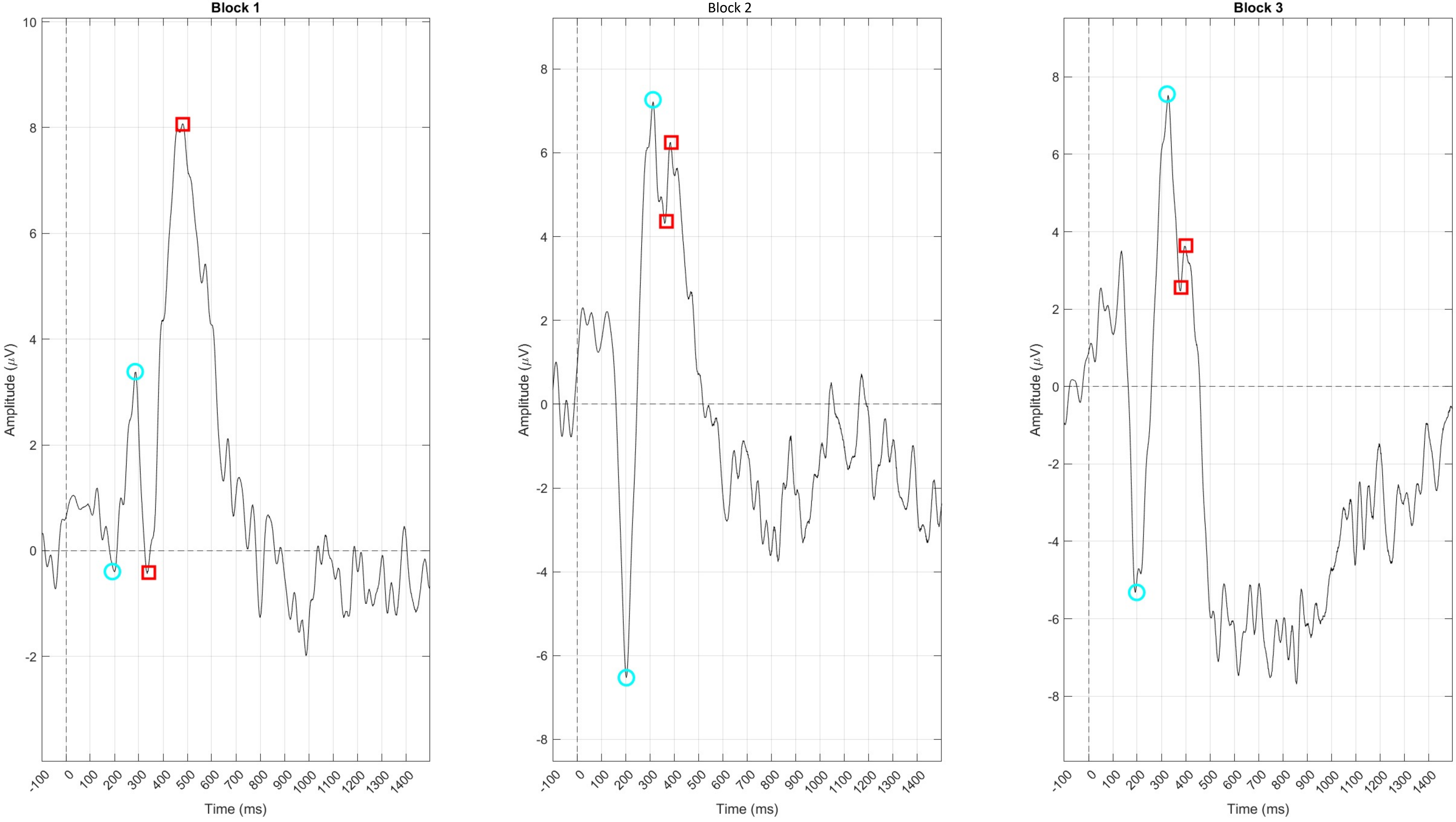
Block 1
Block 2
Block 3
Amplitude (μV)
Time (ms)
-100
0
100
200
300
400
500
600
700
800
900
1000
1100
1200
1300
1400
10
8
6
4
2
0
-2
-4
-6
-8

Subject 18

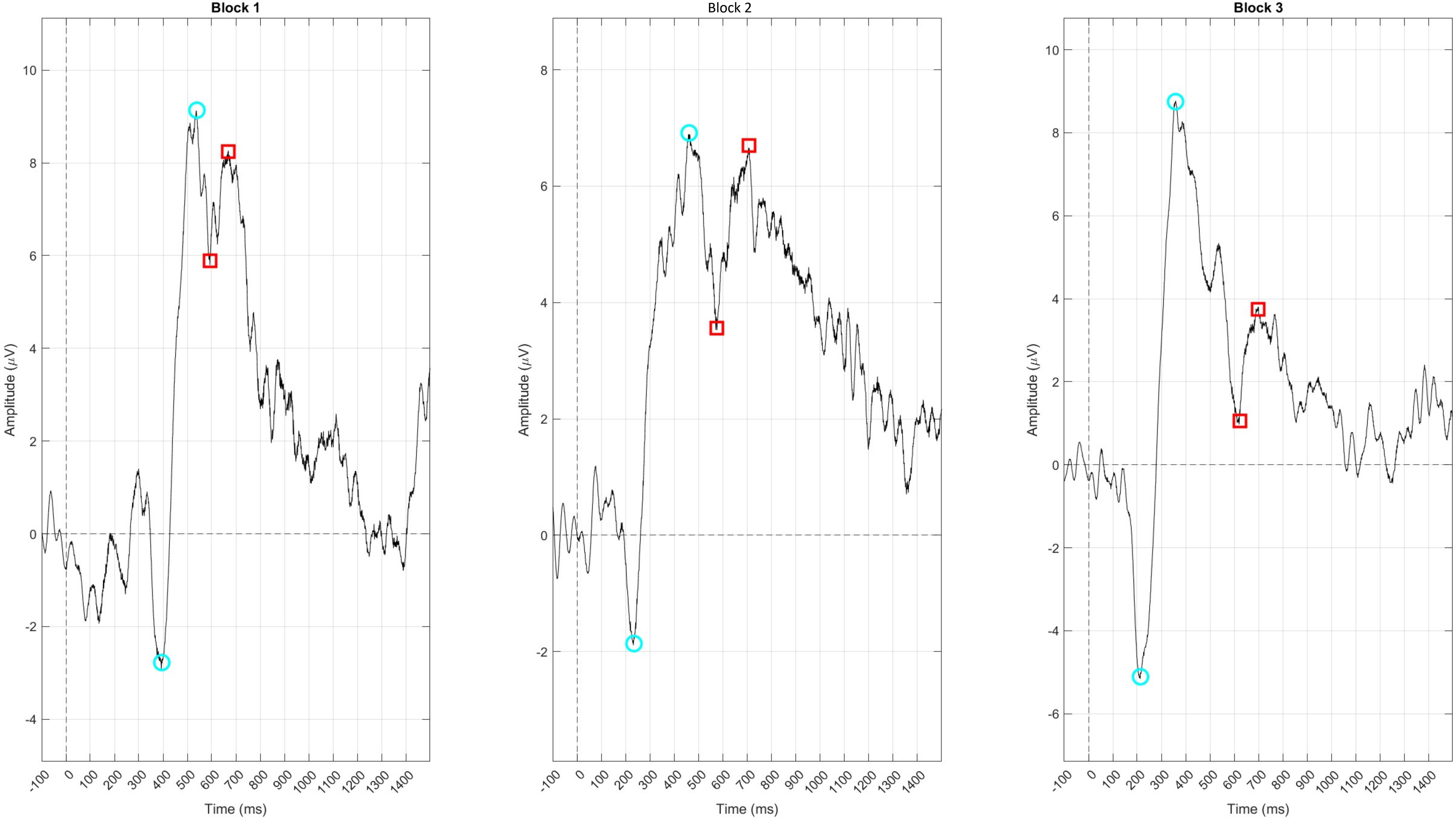

Subject 19

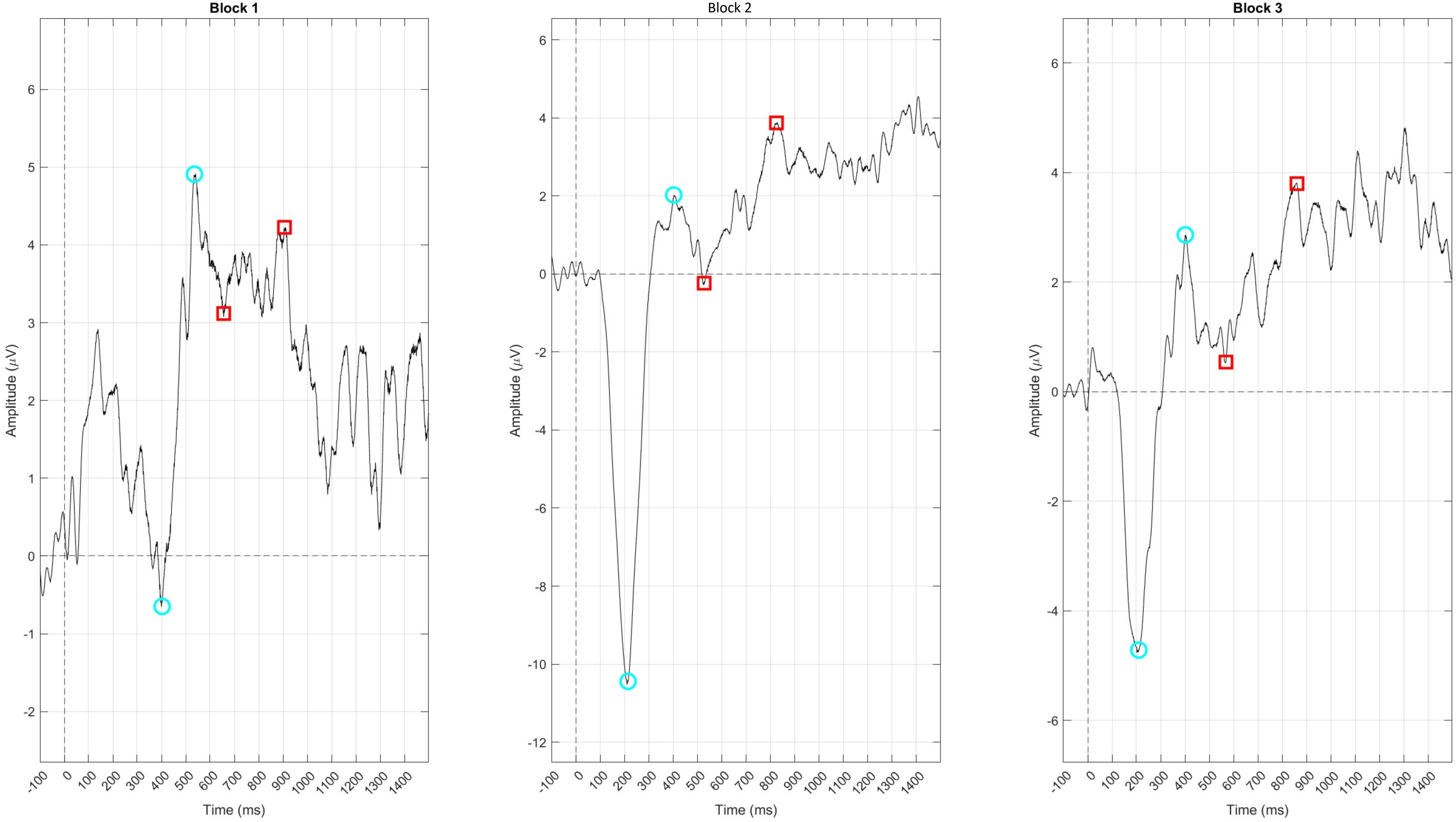

Block 1
Block 2
Block 3
Amplitude (μV)
Time (ms)
-100
0
100
200
300
400
500
600
700
800
900
1000
1100
1200
1300
1400

Subject 20

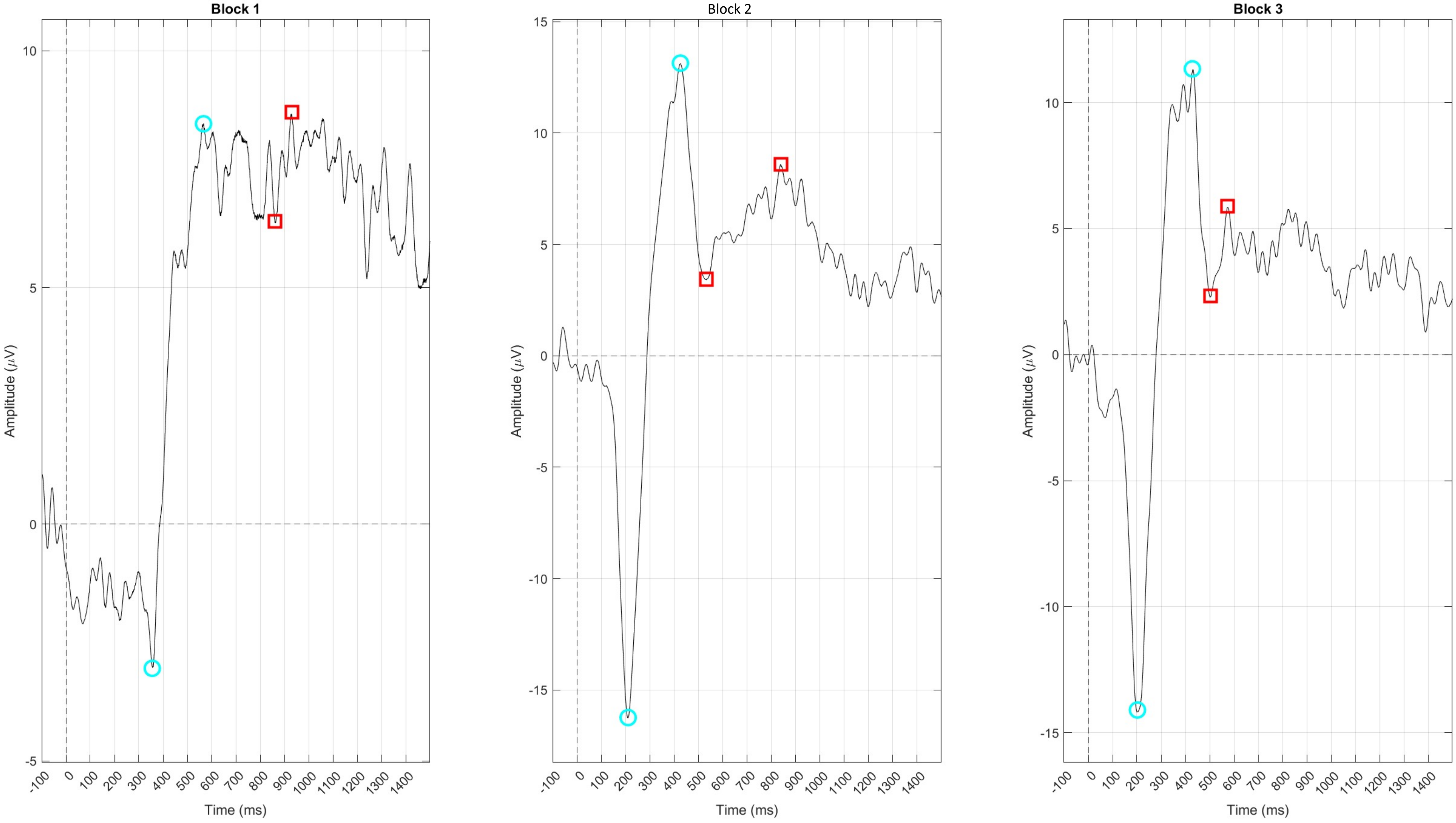
Block 1
Block 2
Block 3
Amplitude (μV)
Time (ms)
-100
0
100
200
300
400
500
600
700
800
900
1000
1100
1200
1300
1400
10
5
0
-5
15
-10
-15

Subject 21

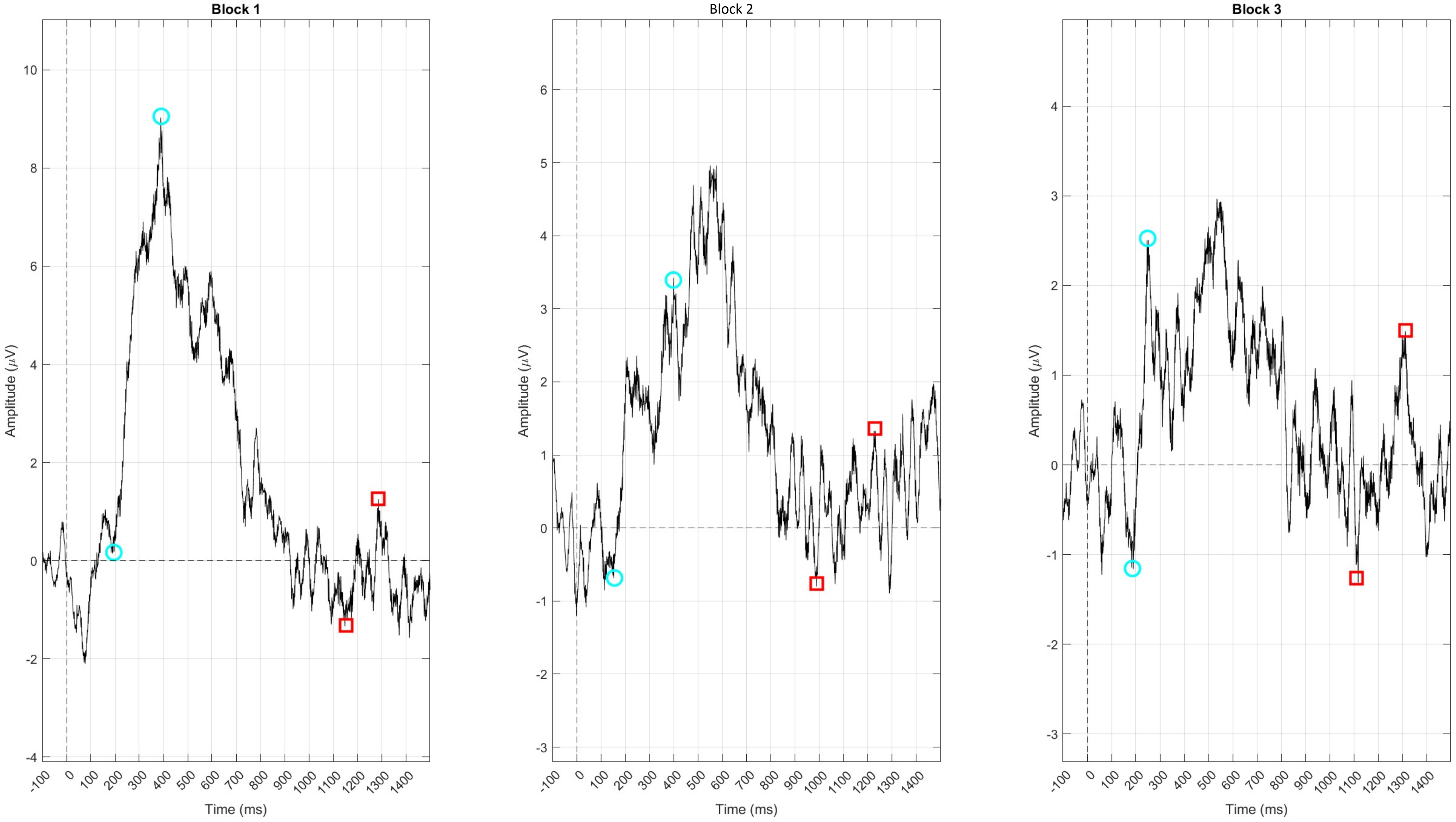

Block 1
Block 2
Block 3
Amplitude (μV)
Time (ms)
-100 0 100 200 300 400 500 600 700 800 900 1000 1100 1200 1300 1400
10 8 6 4 2 0 -2 -4
6 5 4 3 2 1 0 -1 -2 -3
4 3 2 1 0 -1 -2 -3

Subject 22

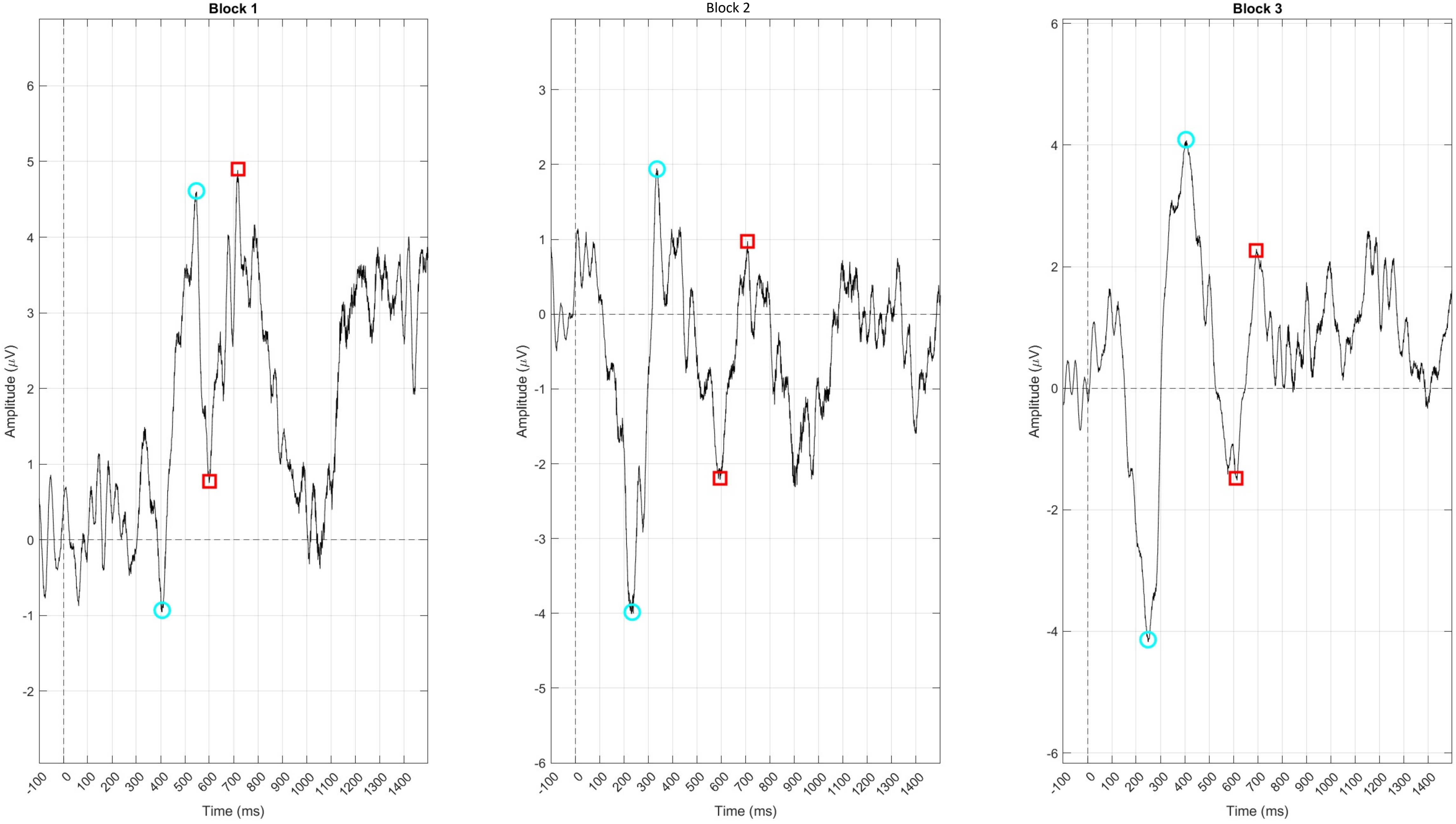

Subject 23

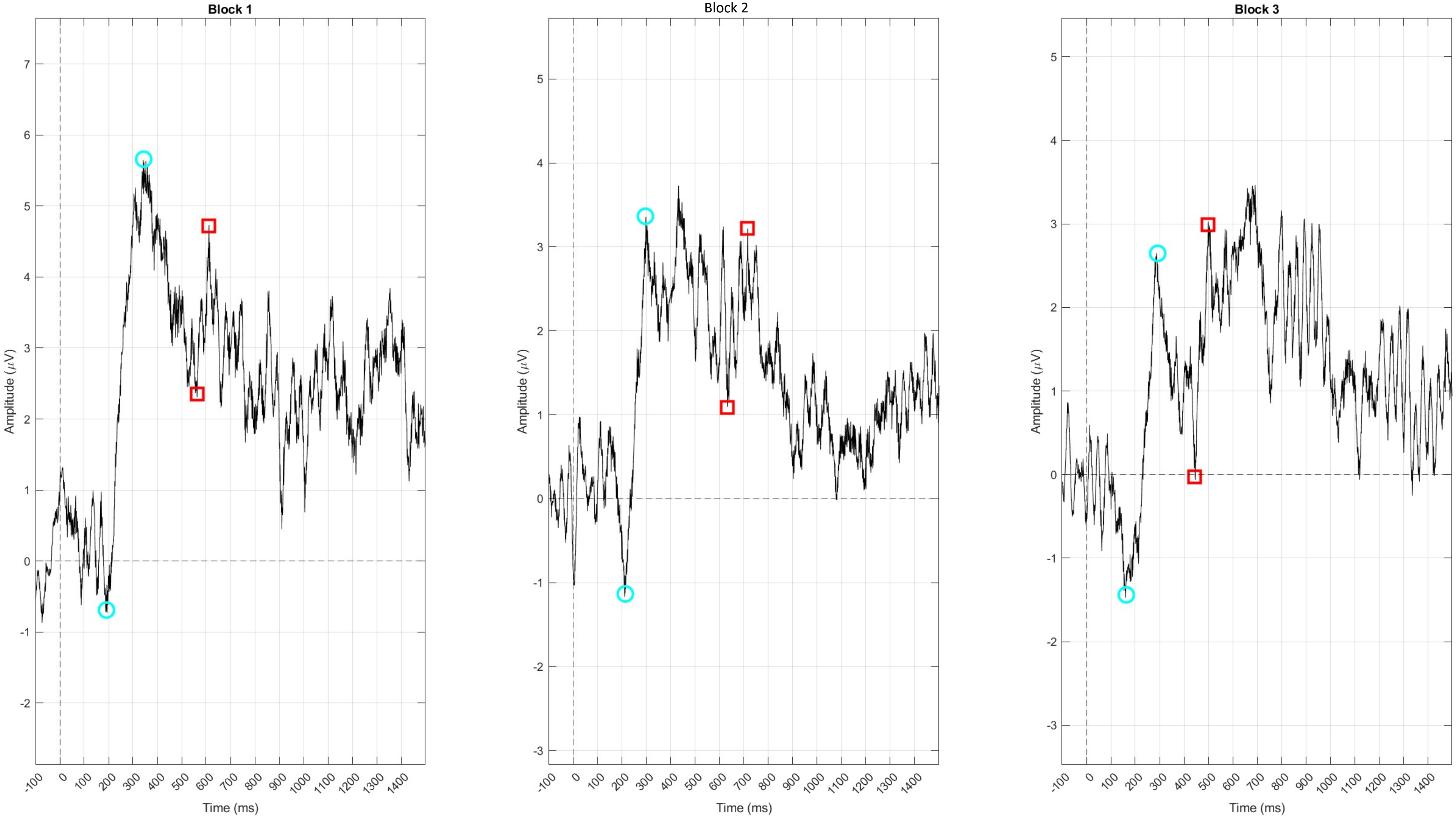

Subject 24 was excluded from analysis

Subject 25

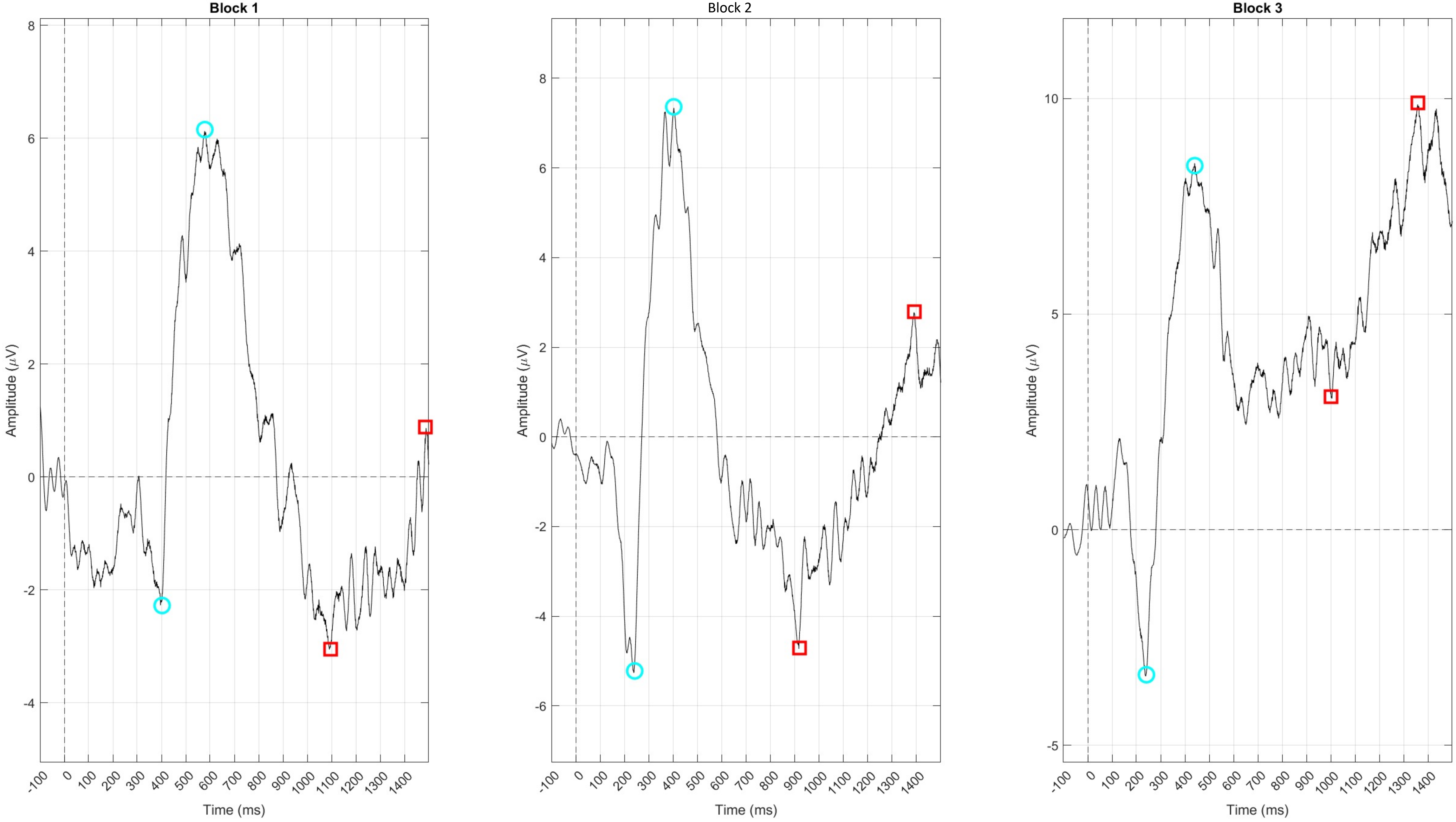

Block 1
Block 2
Block 3
Amplitude (μV)
Time (ms)
-100
0
100
200
300
400
500
600
700
800
900
1000
1100
1200
1300
1400

Subject 26

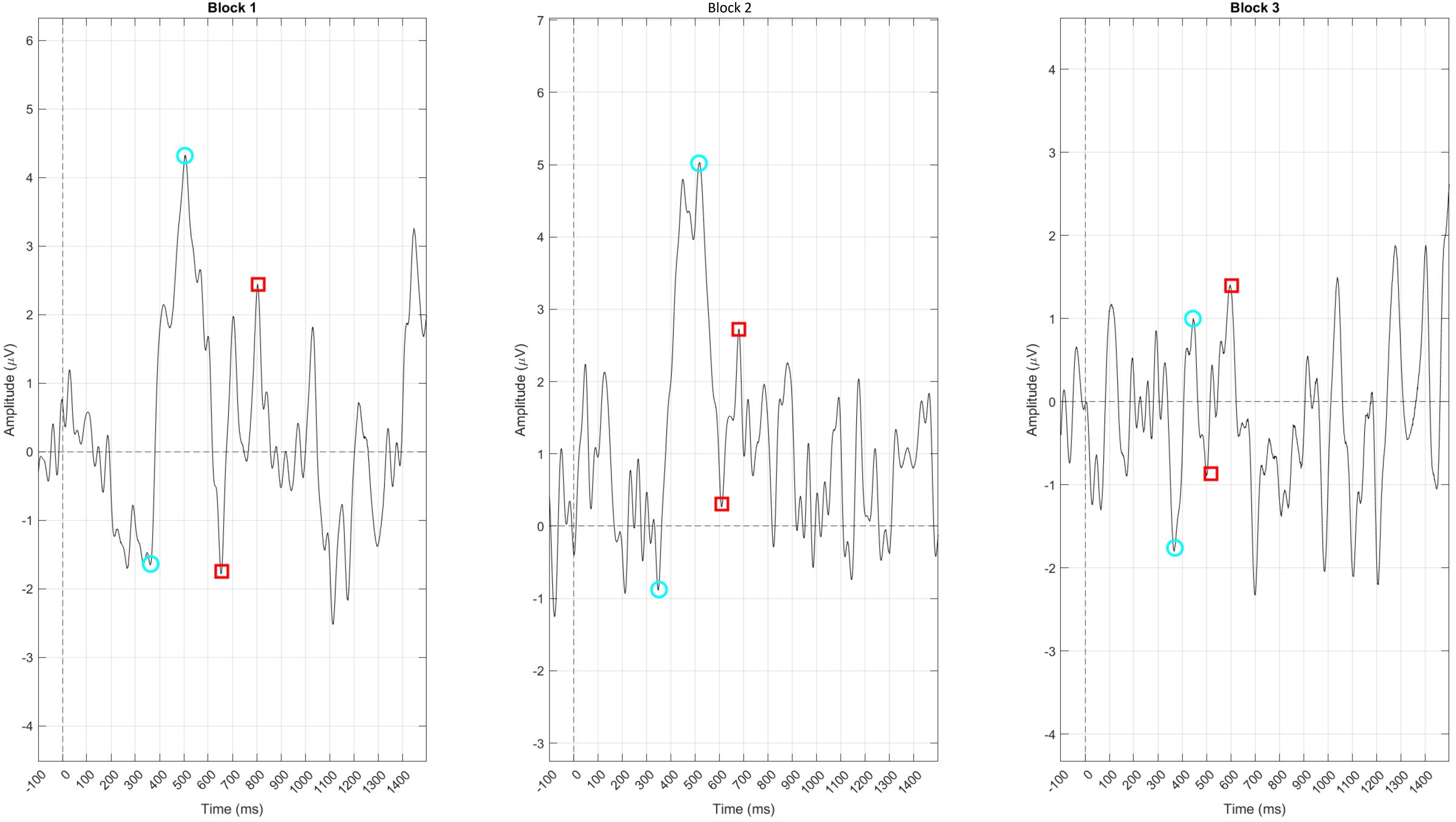
Block 1
Block 2
Block 3
Amplitude (μV)
Time (ms)
-100
0
100
200
300
400
500
600
700
800
900
1000
1100
1200
1300
1400

Subject 27

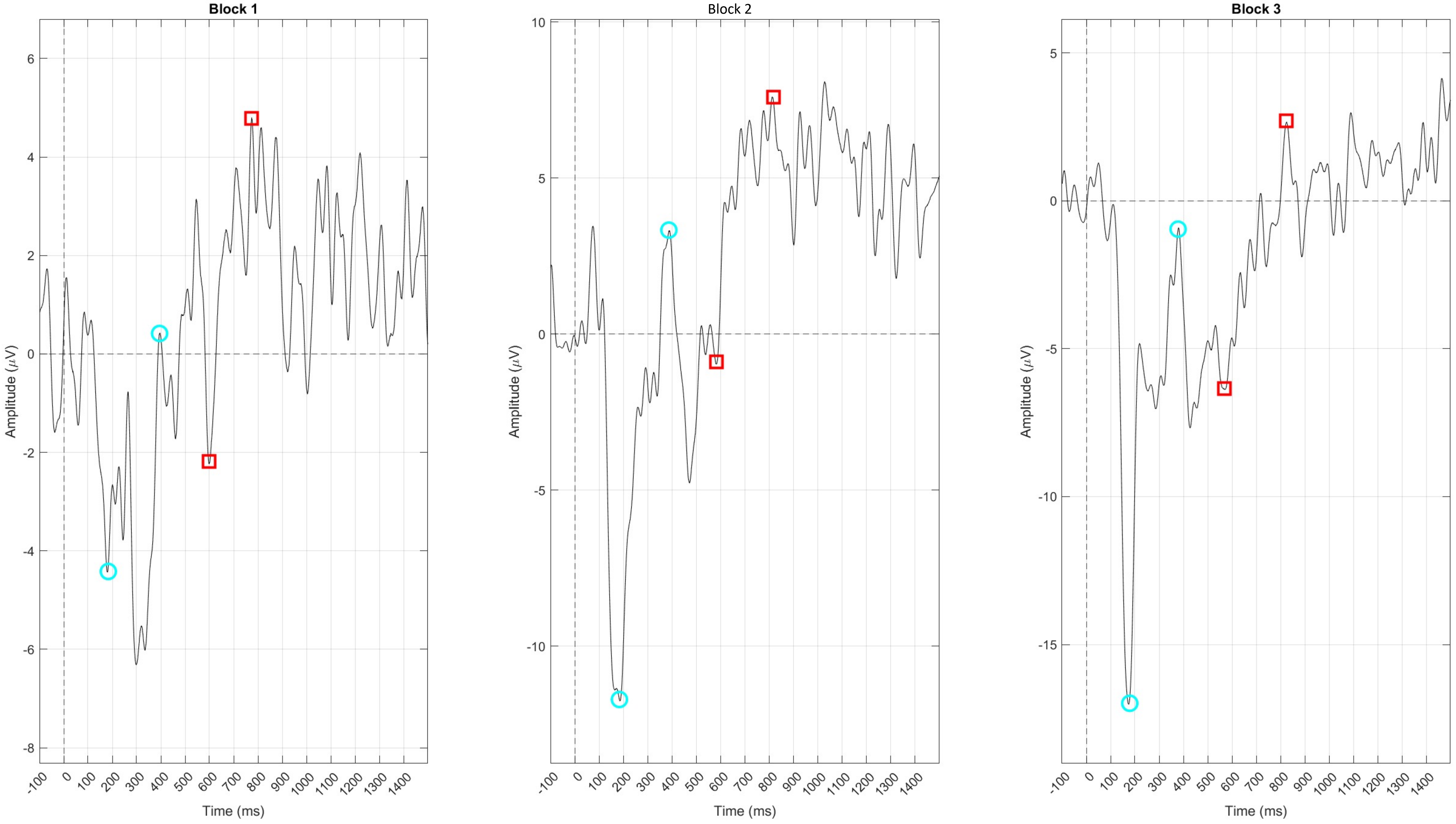

Block 1
Amplitude (μV)
6
4
2
0
-2
-4
-6
-8
Time (ms)
-100
0
100
200
300
400
500
600
700
800
900
1000
1100
1200
1300
1400
Block 2
Amplitude (μV)
10
5
0
-5
-10
Time (ms)
Block 3
Amplitude (μV)
5
0
-5
-10
-15
Time (ms)

Subject 28

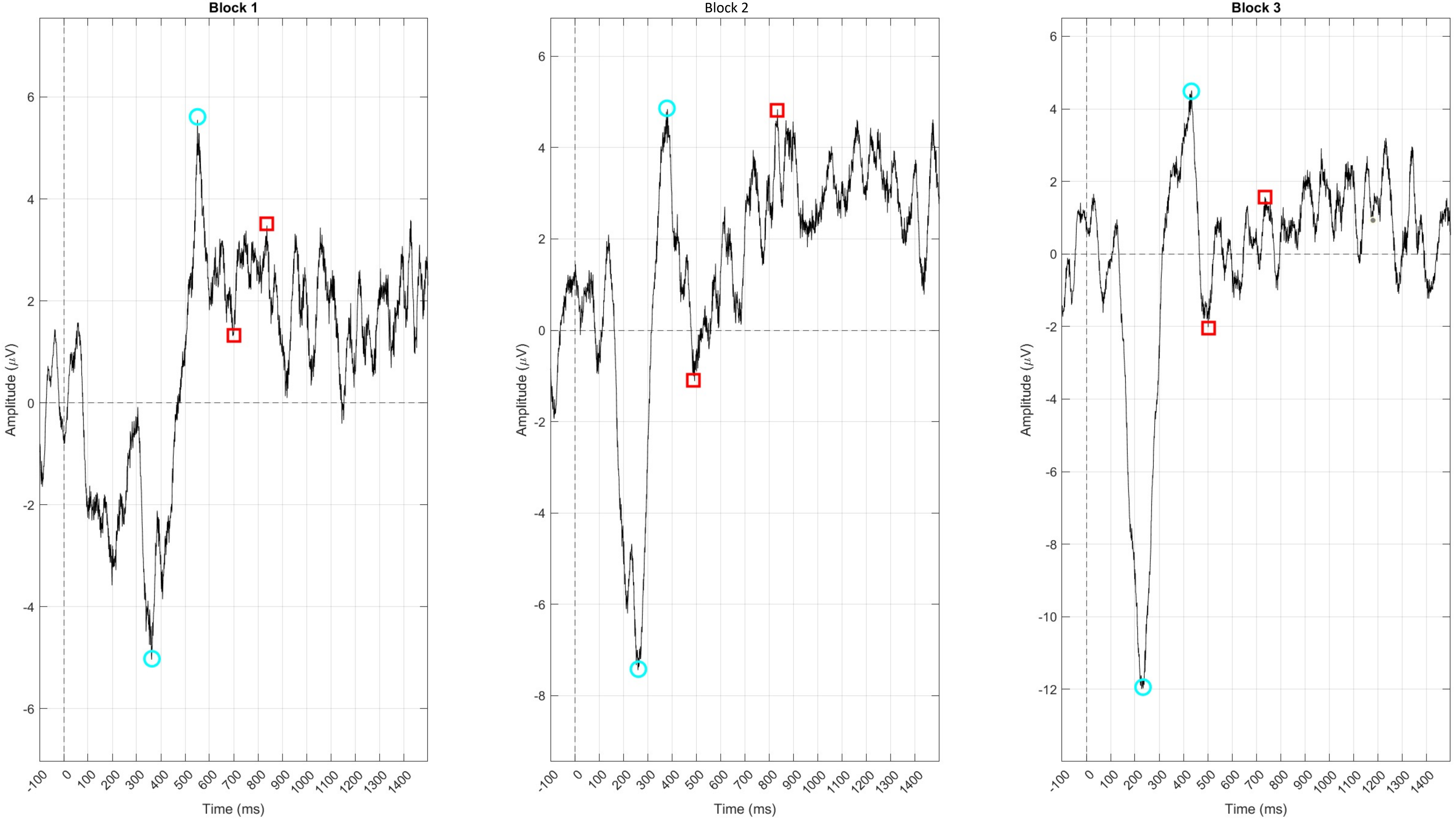
Block 1
Block 2
Block 3
Amplitude (μV)
Time (ms)
-100
0
100
200
300
400
500
600
700
800
900
1000
1100
1200
1300
1400
6
4
2
0
-2
-4
-6
-8
-10
-12

Subject 29

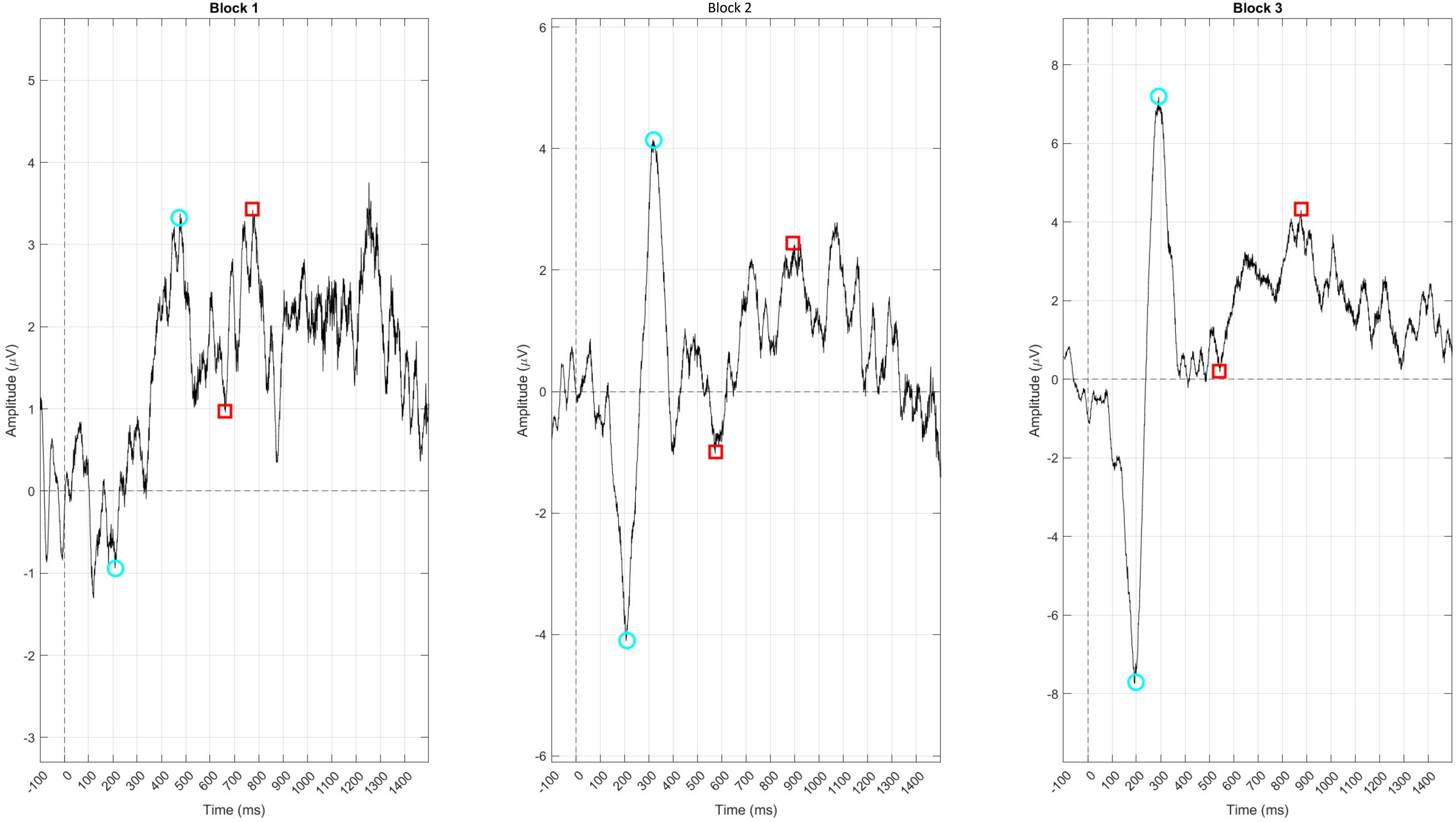
Block 1
Block 2
Block 3
Amplitude (μV)
Time (ms)
-100
0
100
200
300
400
500
600
700
800
900
1000
1100
1200
1300
1400
5
4
3
2
1
0
-1
-2
-3
6
-4
-6
8
-8

Subject 30

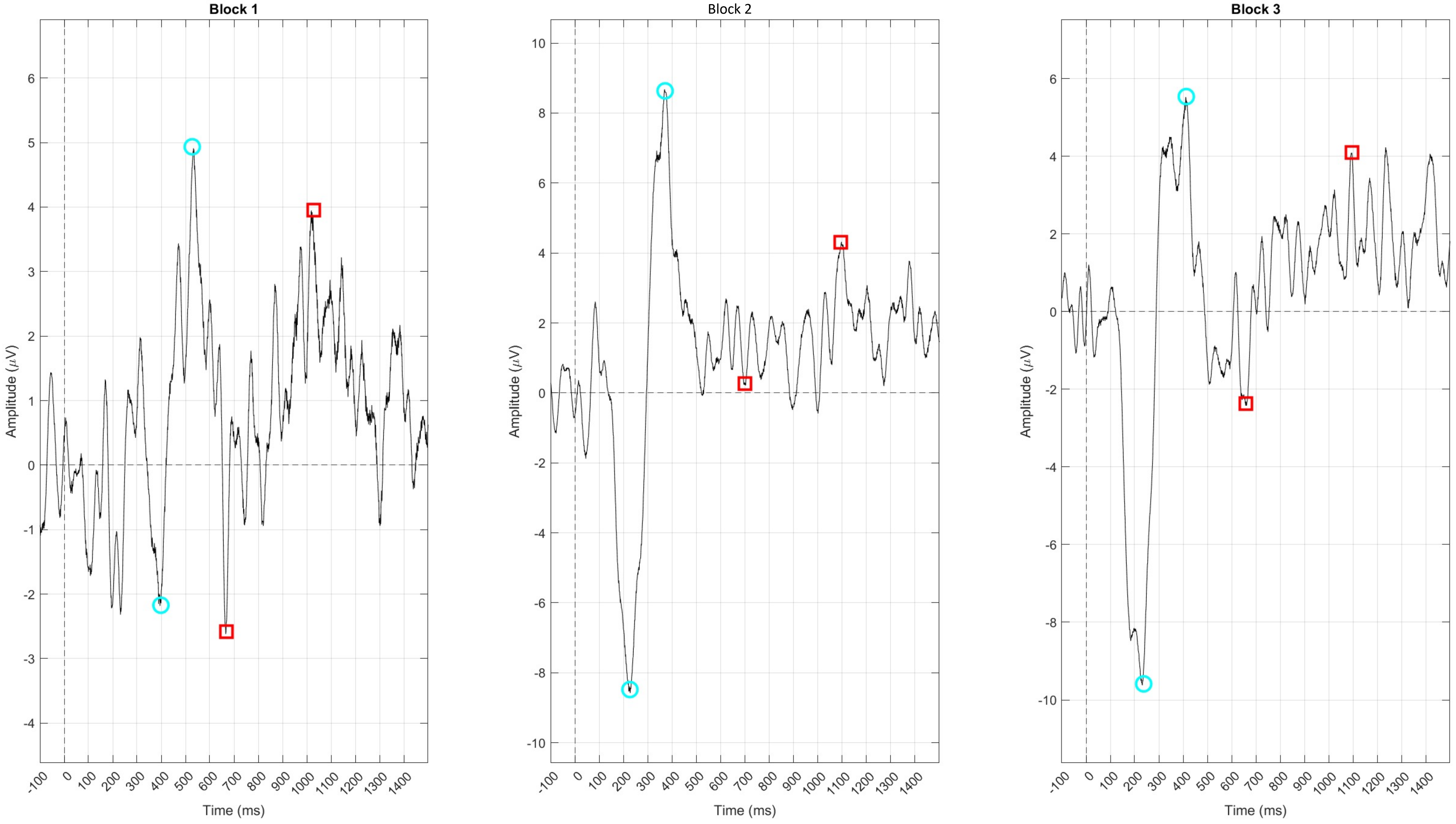

Block 1
Block 2
Block 3
Amplitude (μV)
Time (ms)
-100
0
100
200
300
400
500
600
700
800
900
1000
1100
1200
1300
1400

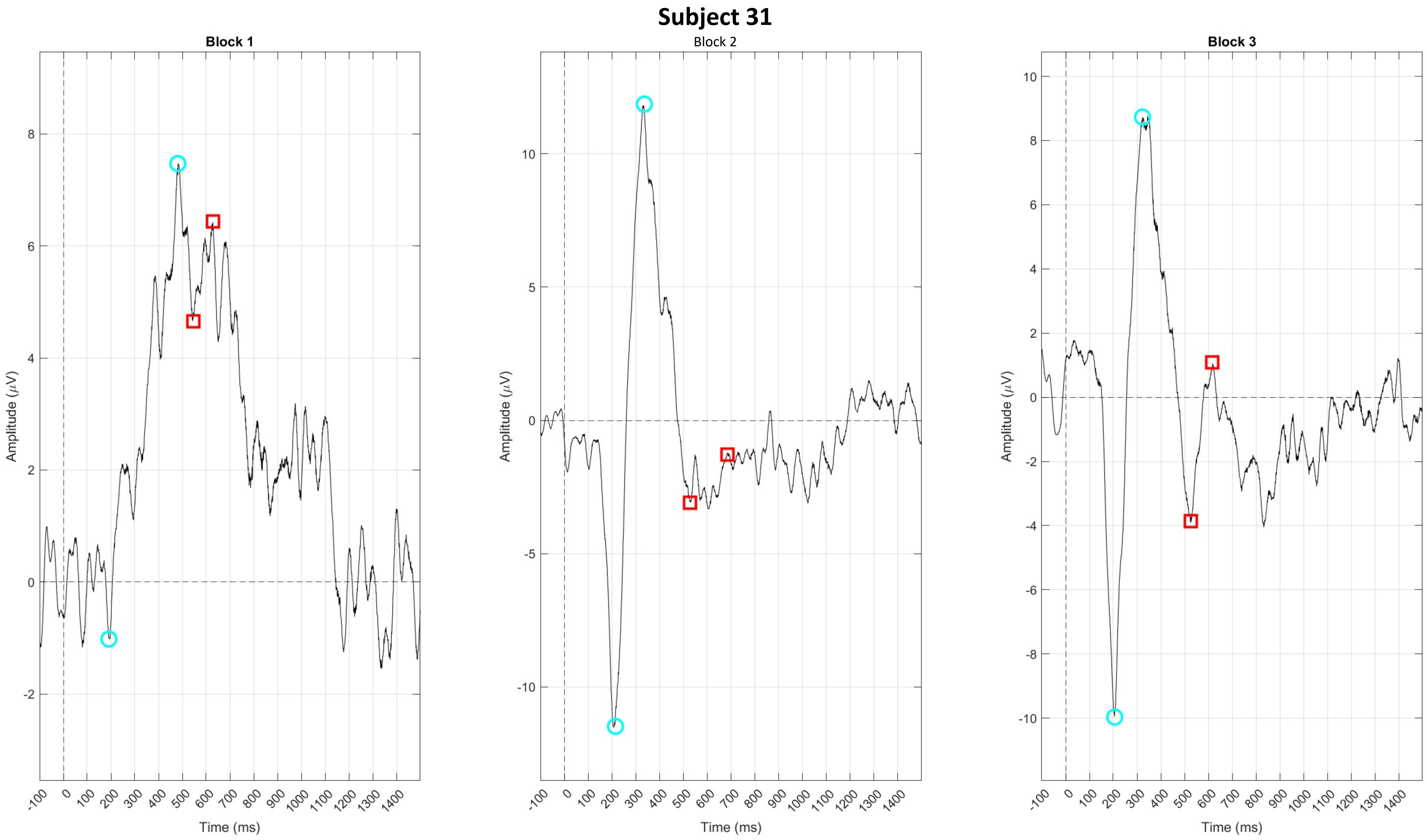
Subject 31
Block 1
Block 2
Block 3
Amplitude (μV)
Time (ms)

Subject 32

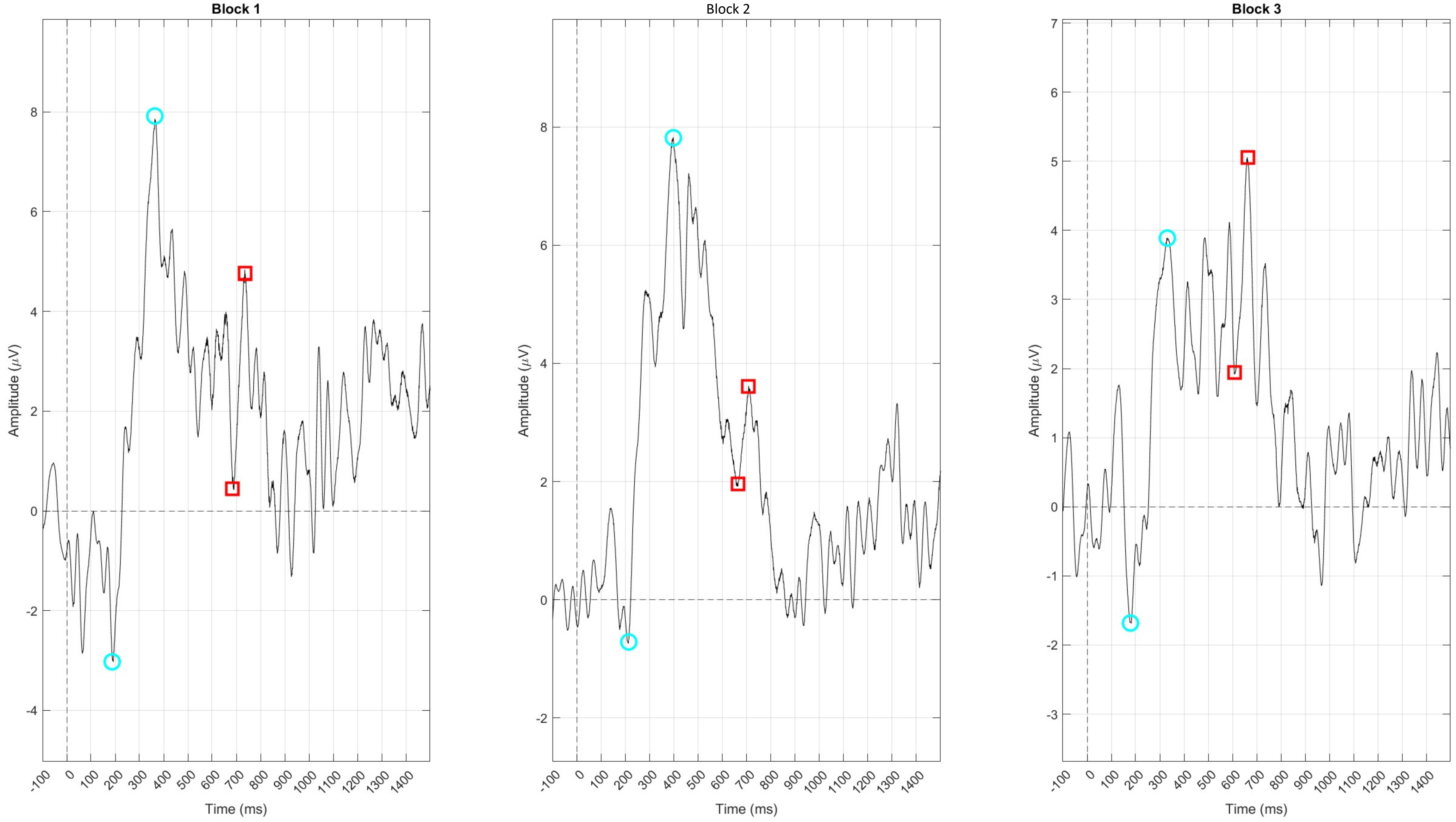

Block 1
Block 2
Block 3
Amplitude (μV)
Time (ms)
-100
0
100
200
300
400
500
600
700
800
900
1000
1100
1200
1300
1400

Subject 33

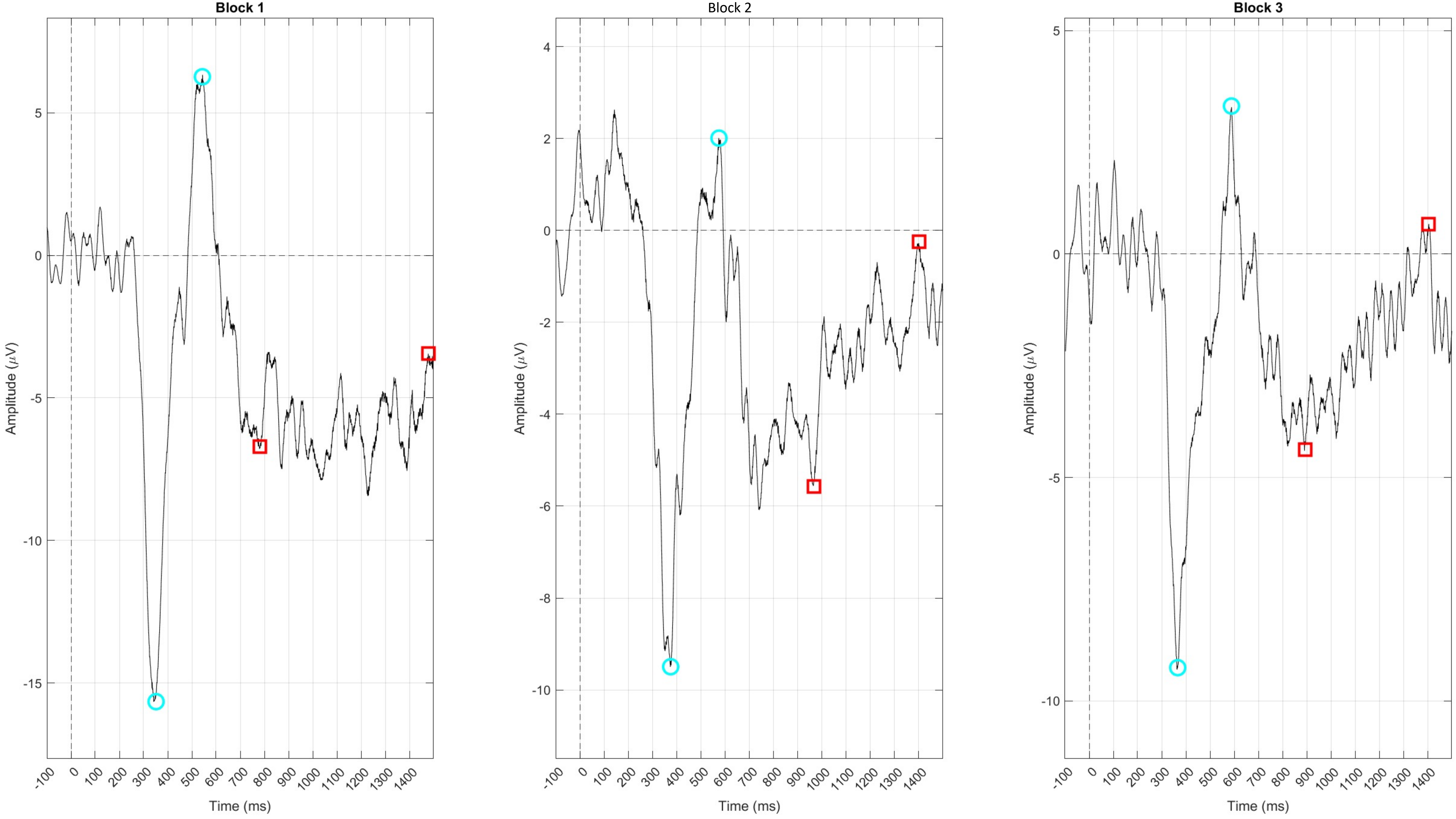
Block 1
Block 2
Block 3
Amplitude (μV)
Time (ms)
-100
0
100
200
300
400
500
600
700
800
900
1000
1100
1200
1300
1400
5
0
-5
-10
-15
4
2
-2
-4
-6
-8

Subject 34

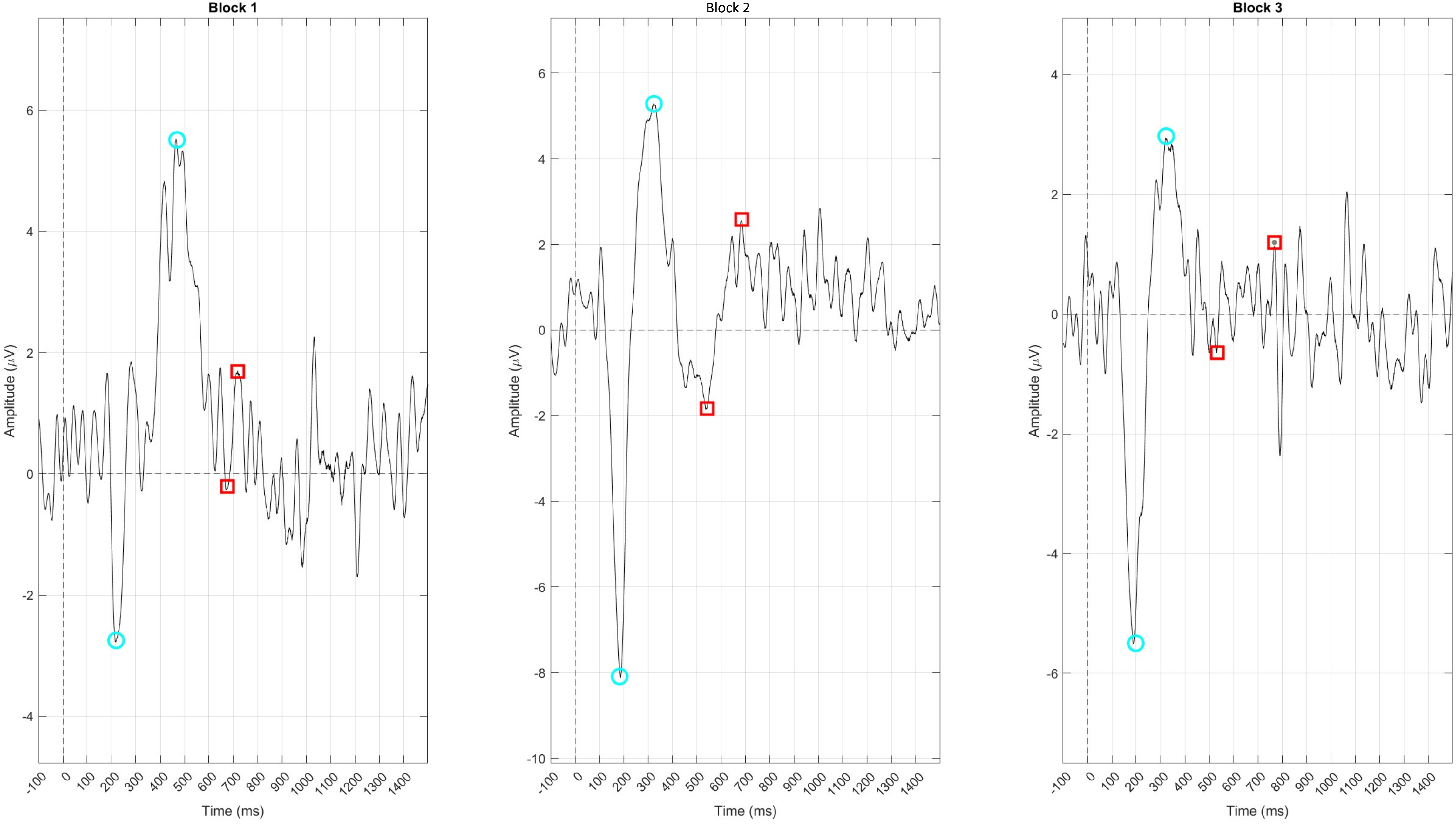

Block 1
Block 2
Block 3
Amplitude (μV)
Time (ms)
-100
0
100
200
300
400
500
600
700
800
900
1000
1100
1200
1300
1400

Subject 35

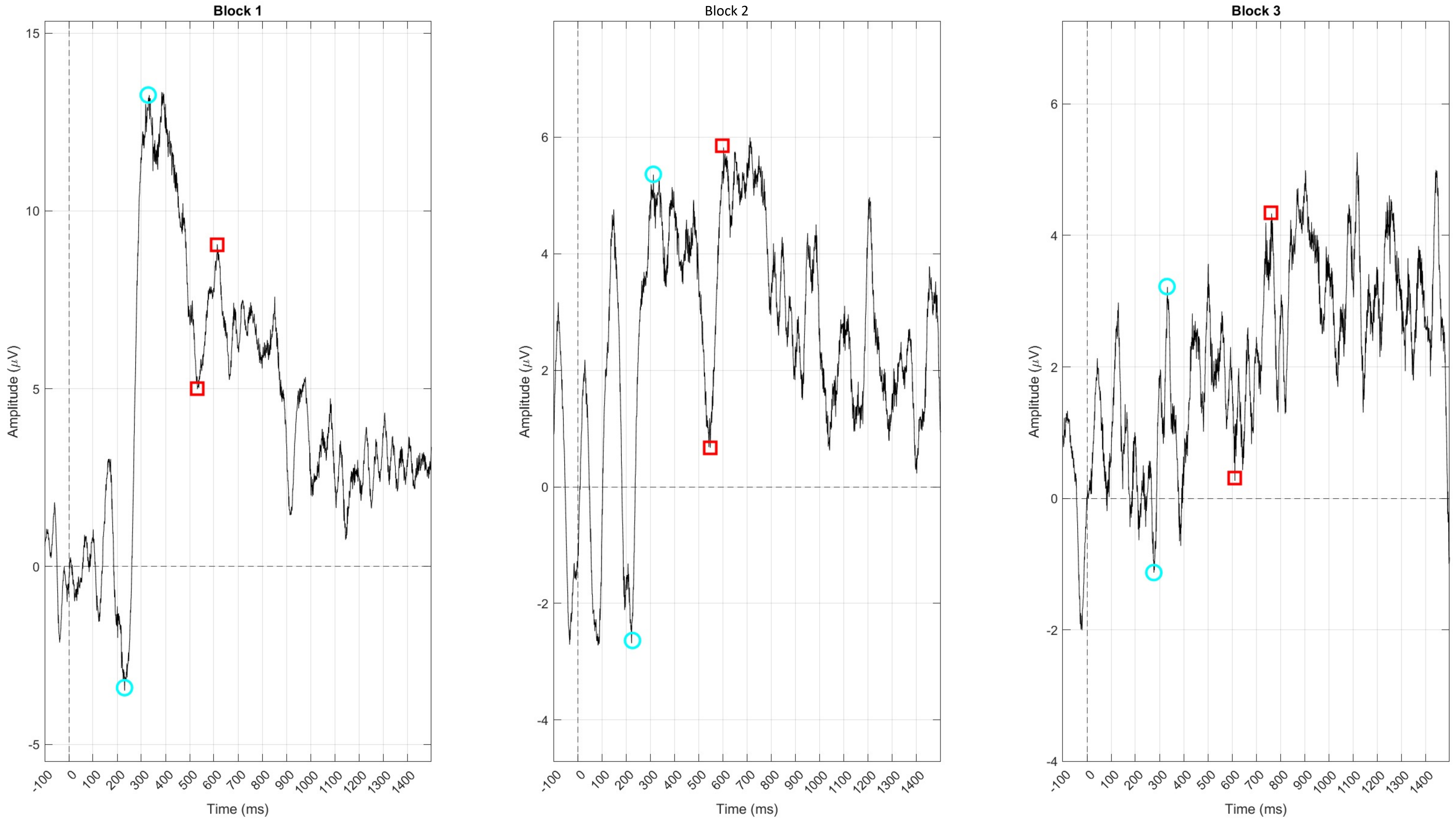
Block 1
Block 2
Block 3
Amplitude (μV)
Time (ms)
15
10
5
0
-5
6
4
2
-2
-4
-100
0
100
200
300
400
500
600
700
800
900
1000
1100
1200
1300
1400

Subject 36

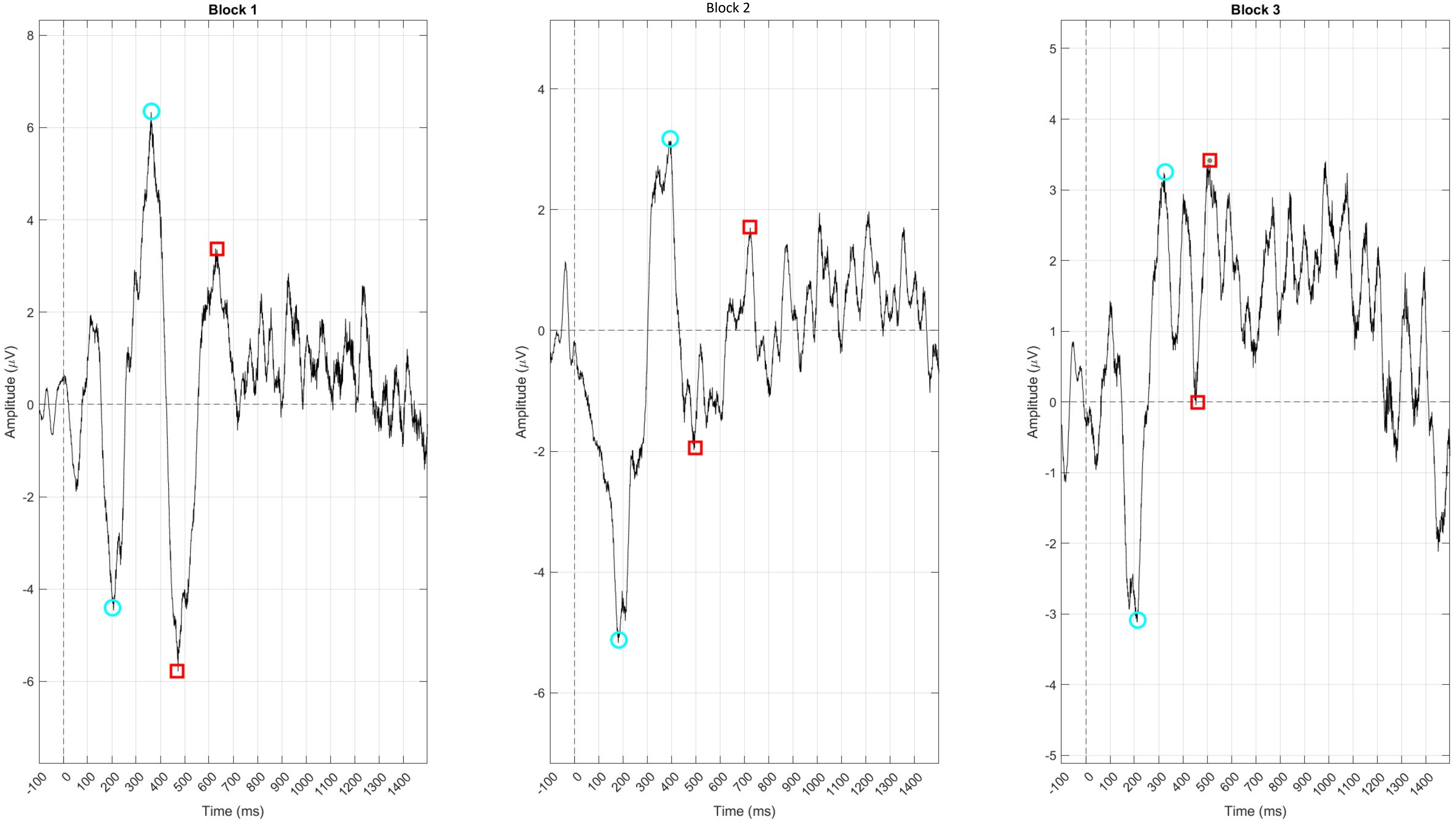

Subject 37

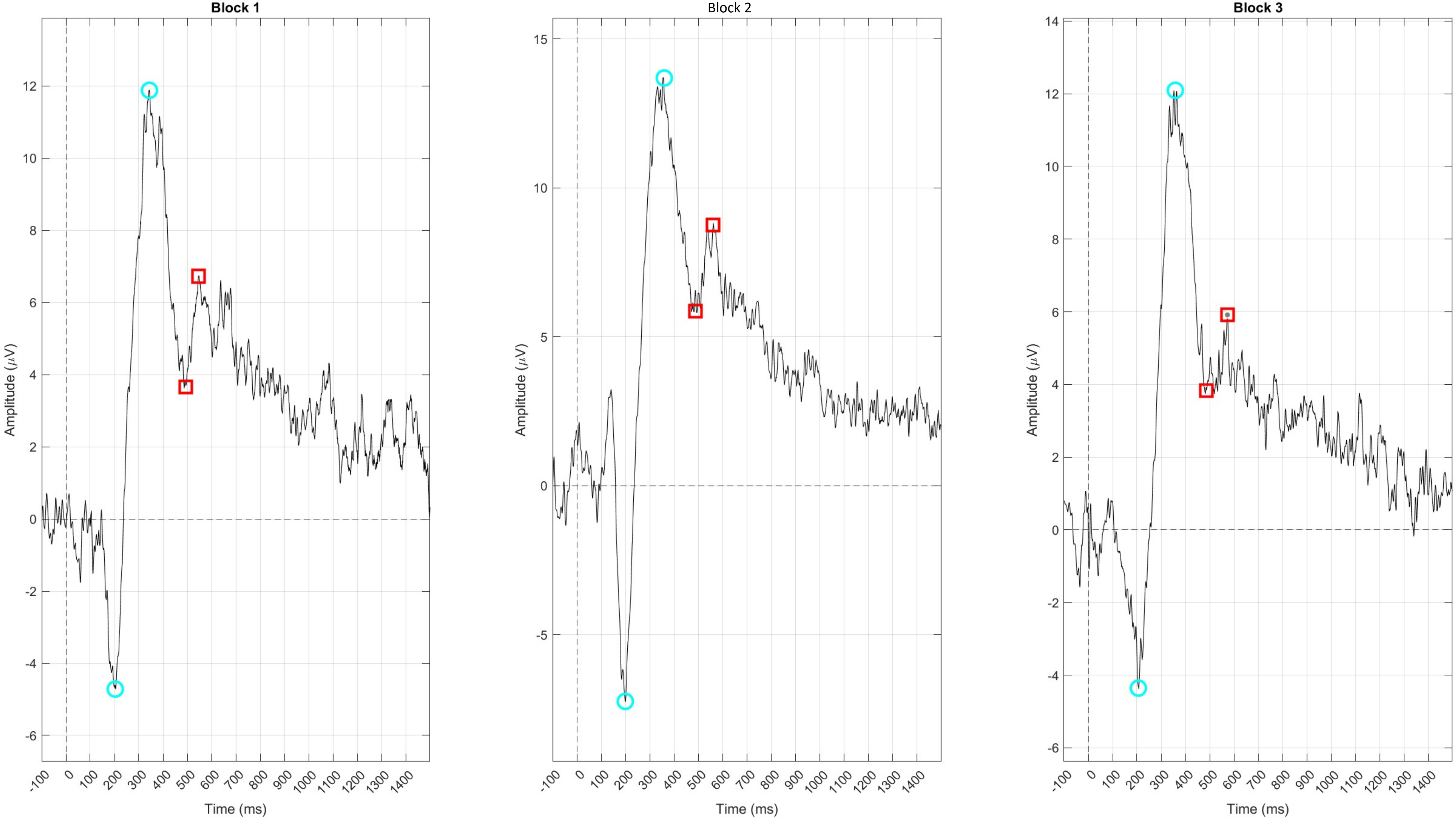
Block 1
Block 2
Block 3
Amplitude (μV)
Time (ms)
-100
0
100
200
300
400
500
600
700
800
900
1000
1100
1200
1300
1400
12
10
8
6
4
2
0
-2
-4
-6
15
5
-5
14

Subject 38

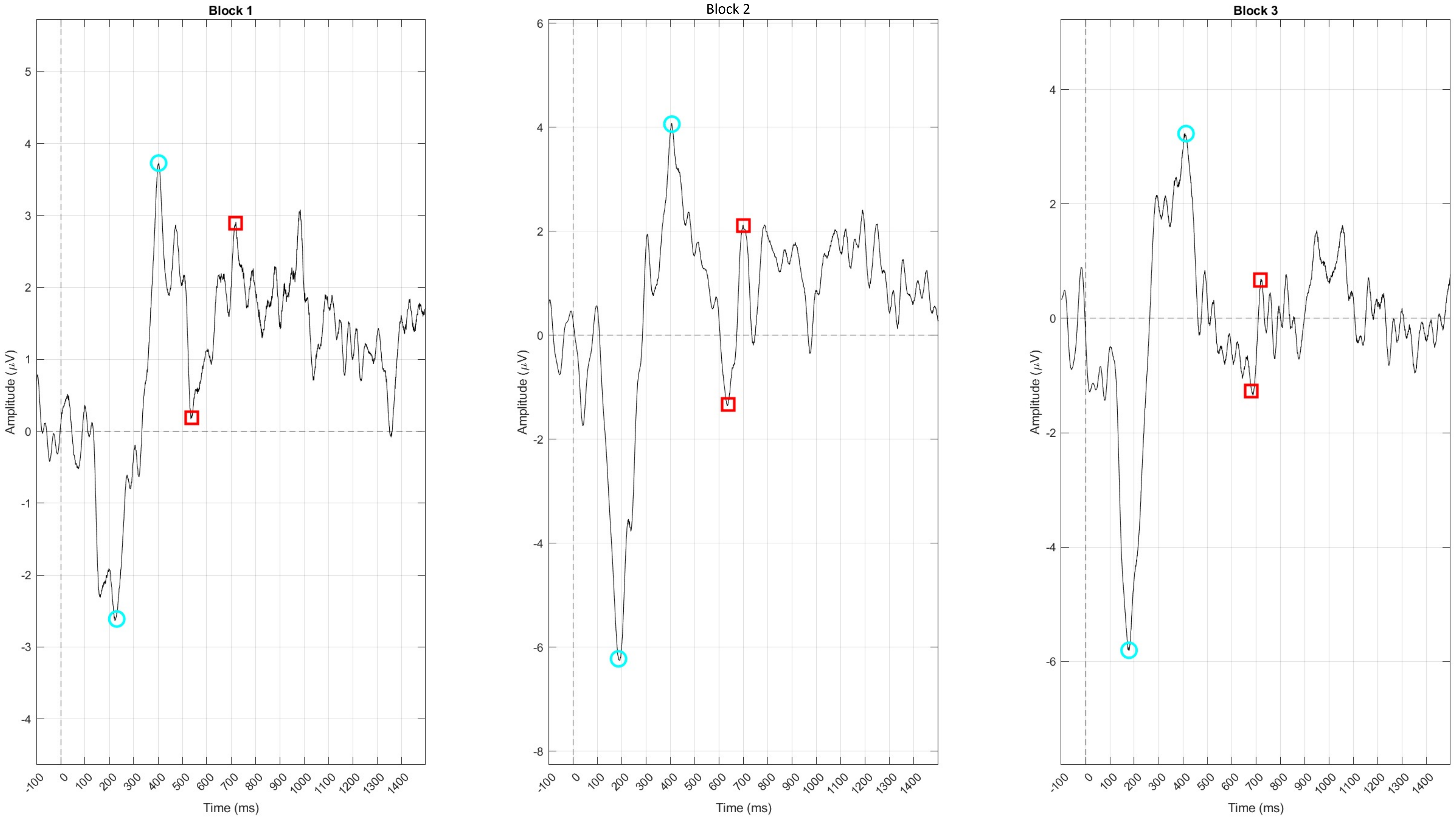

Subject 39

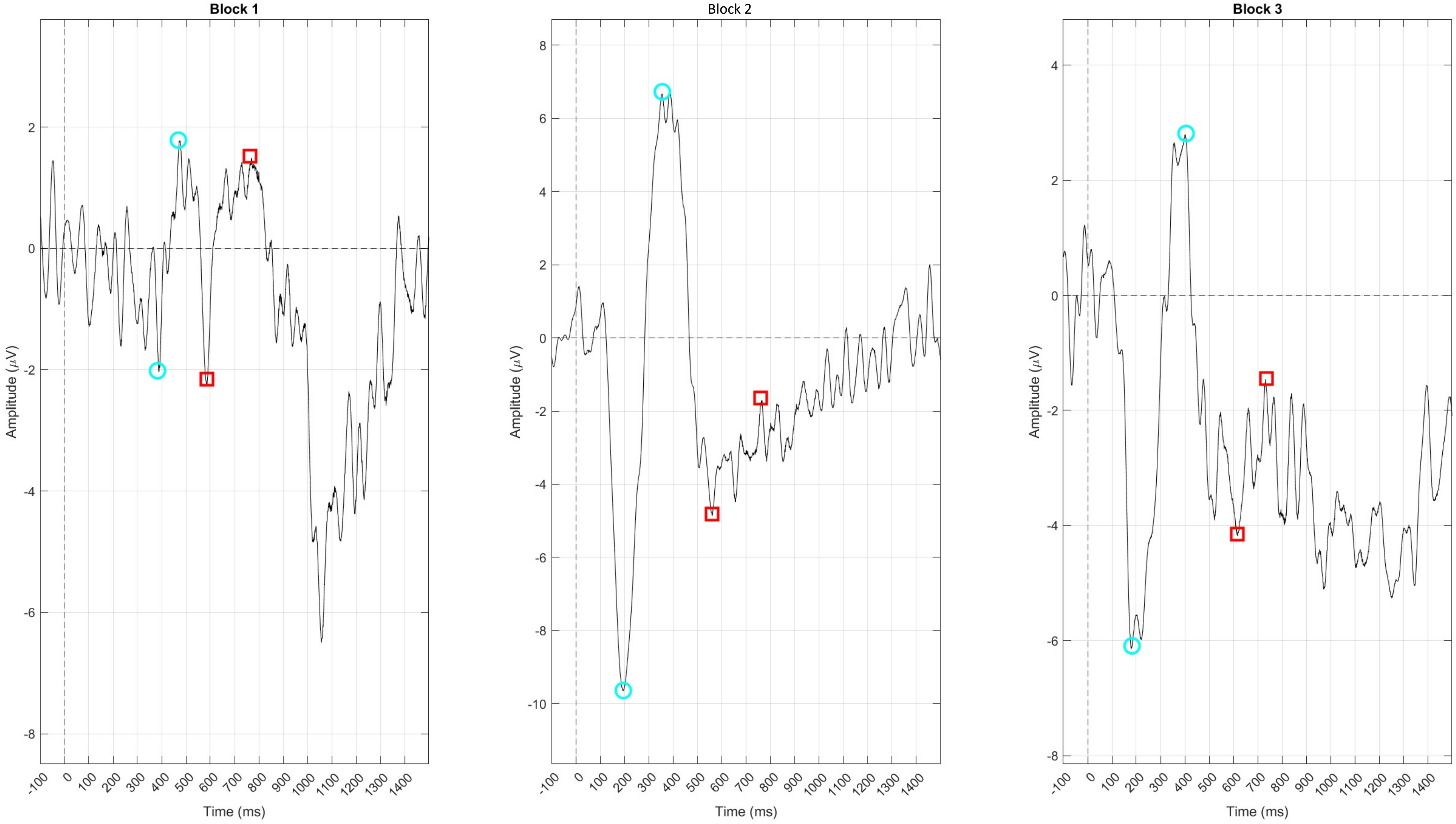
Block 1
Block 2
Block 3
Amplitude (μV)
Time (ms)
-100
0
100
200
300
400
500
600
700
800
900
1000
1100
1200
1300
1400
2
0
-2
-4
-6
-8
8
6
4
-10

Subject 40

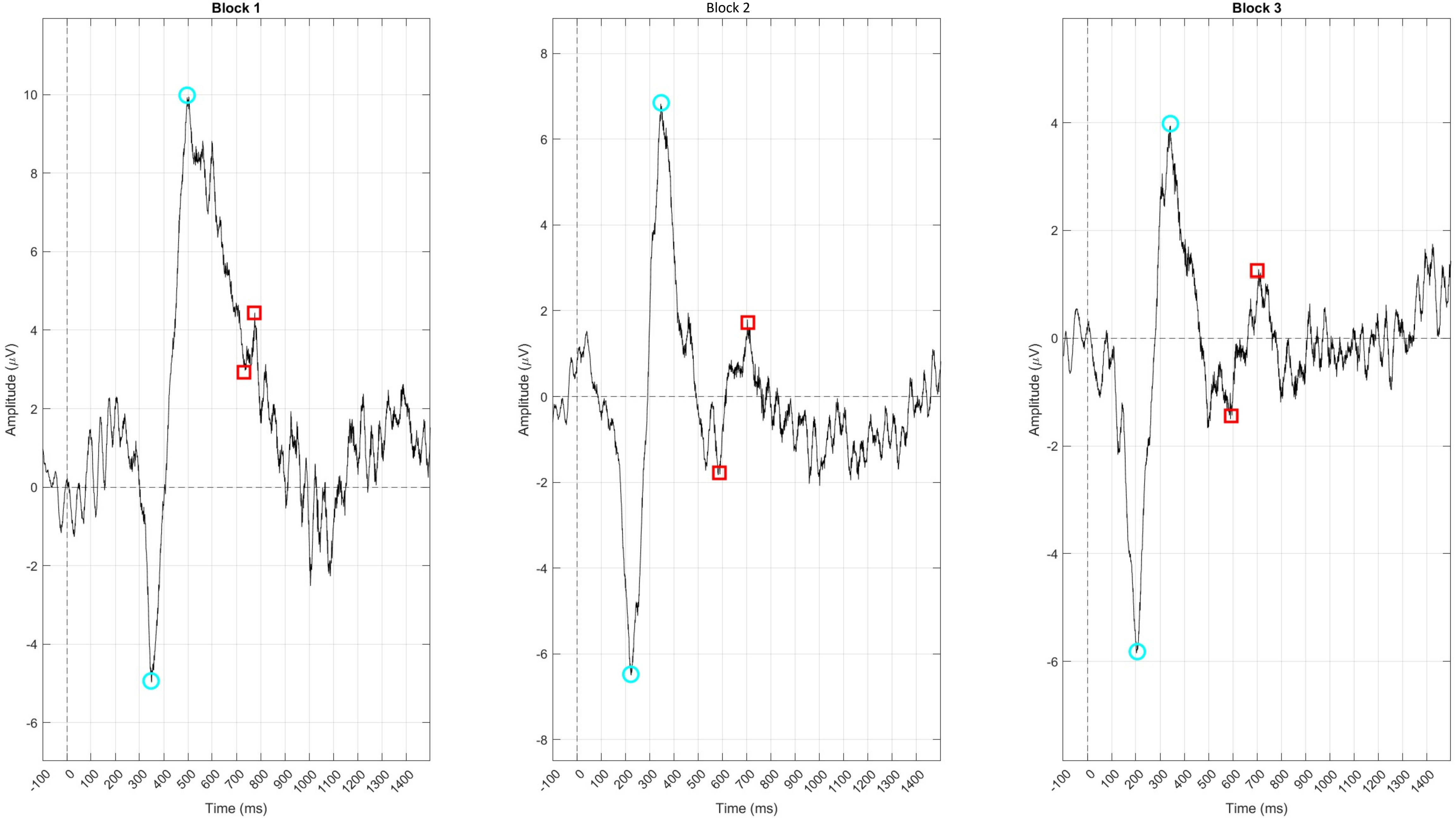

Block 1
Block 2
Block 3
Amplitude (μV)
Time (ms)
-100
0
100
200
300
400
500
600
700
800
900
1000
1100
1200
1300
1400

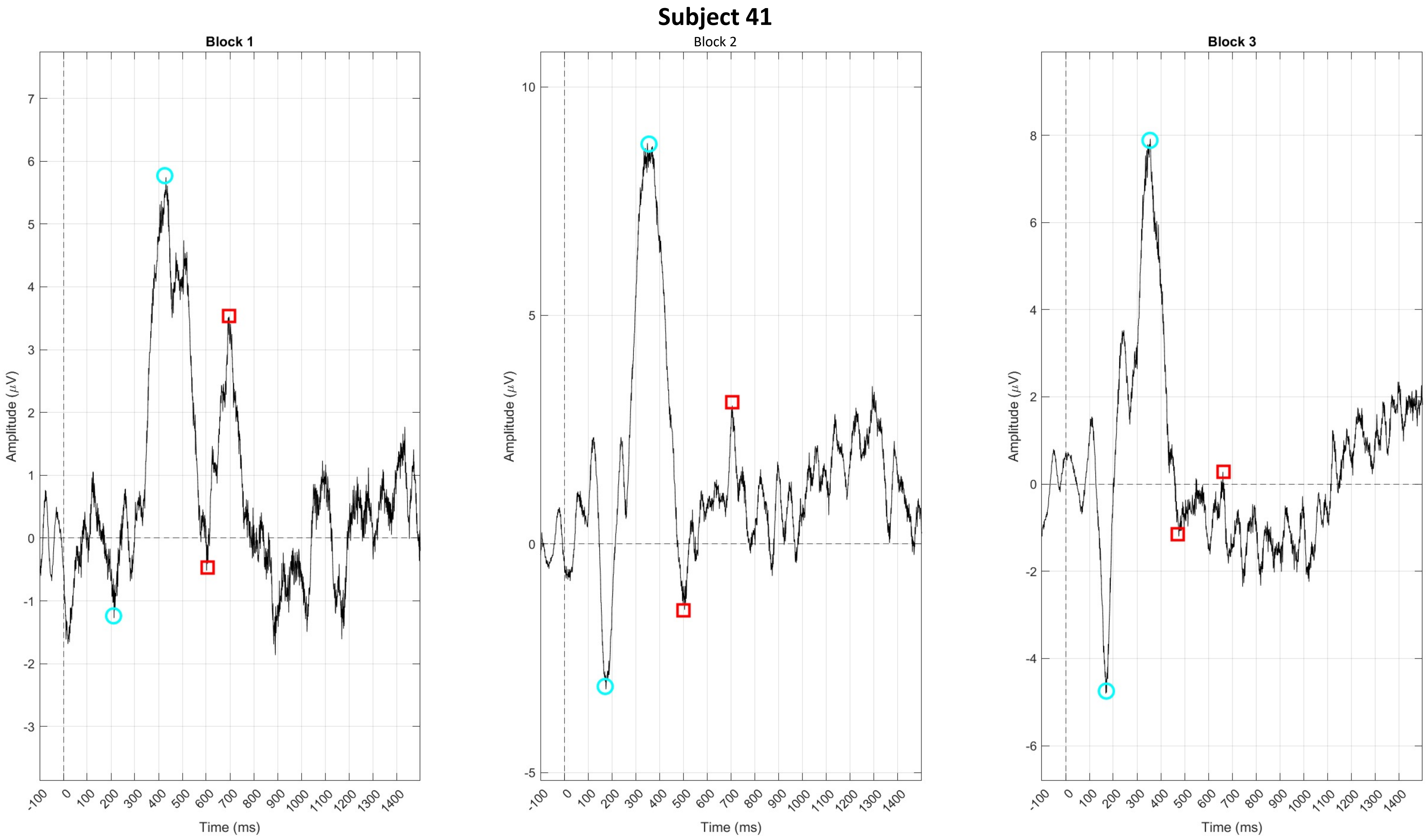
Subject 41
Block 1
Block 2
Block 3
Amplitude (μV)
Time (ms)
-100
0
100
200
300
400
500
600
700
800
900
1000
1100
1200
1300
1400

Subject 42

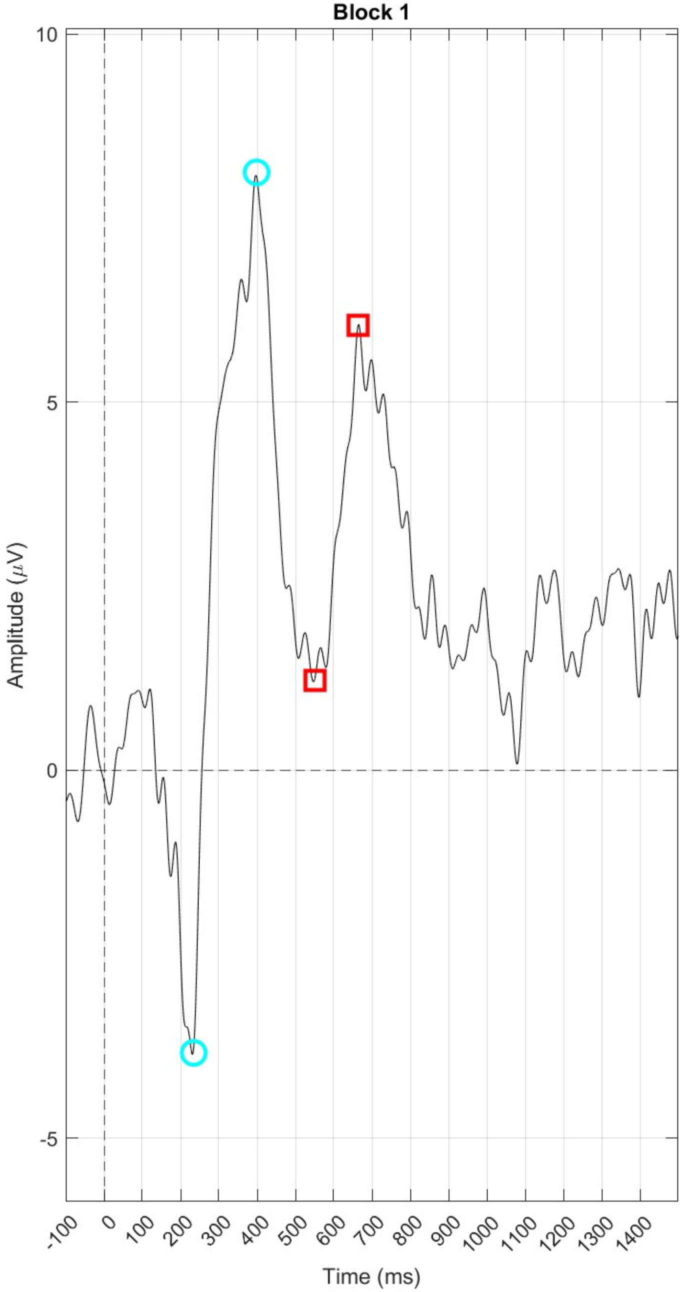
Block 1
10
5
0
-5
Amplitude (μV)
-100 0 100 200 300 400 500 600 700 800 900 1000 1100 1200 1300 1400
Time (ms)

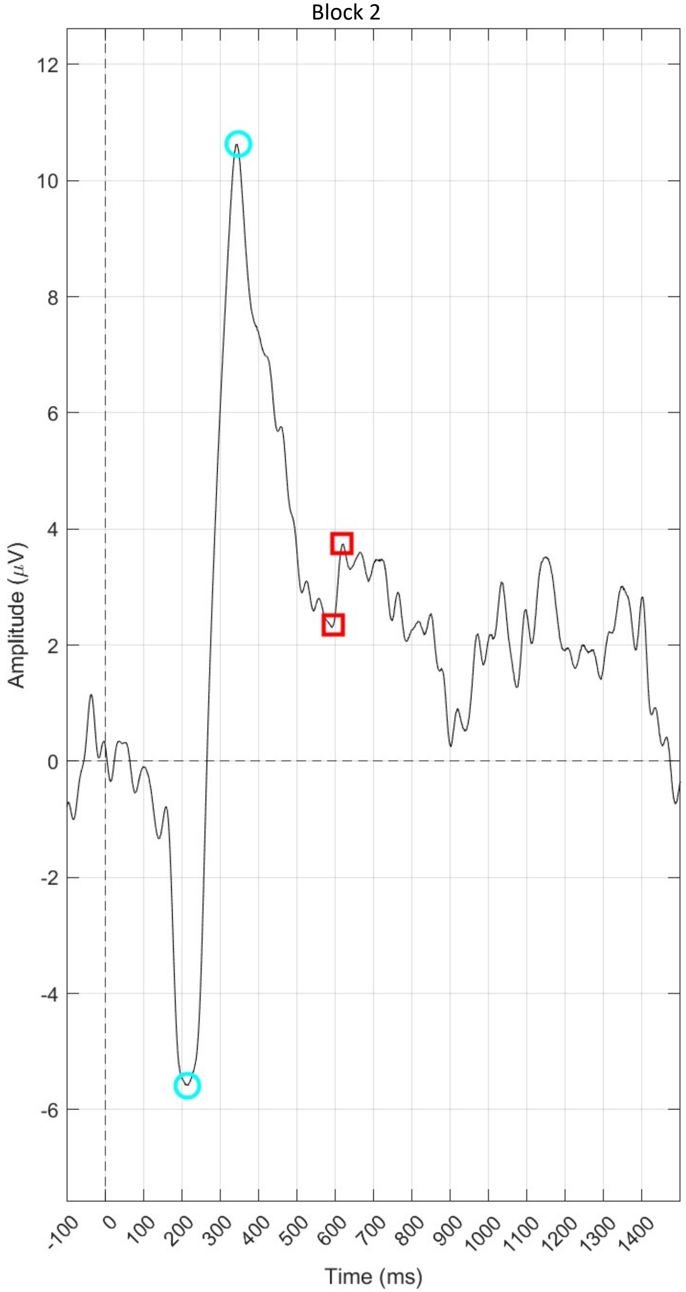
Block 2
12
10
8
6
4
2
0
-2
-4
-6
Amplitude (μV)
-100 0 100 200 300 400 500 600 700 800 900 1000 1100 1200 1300 1400
Time (ms)

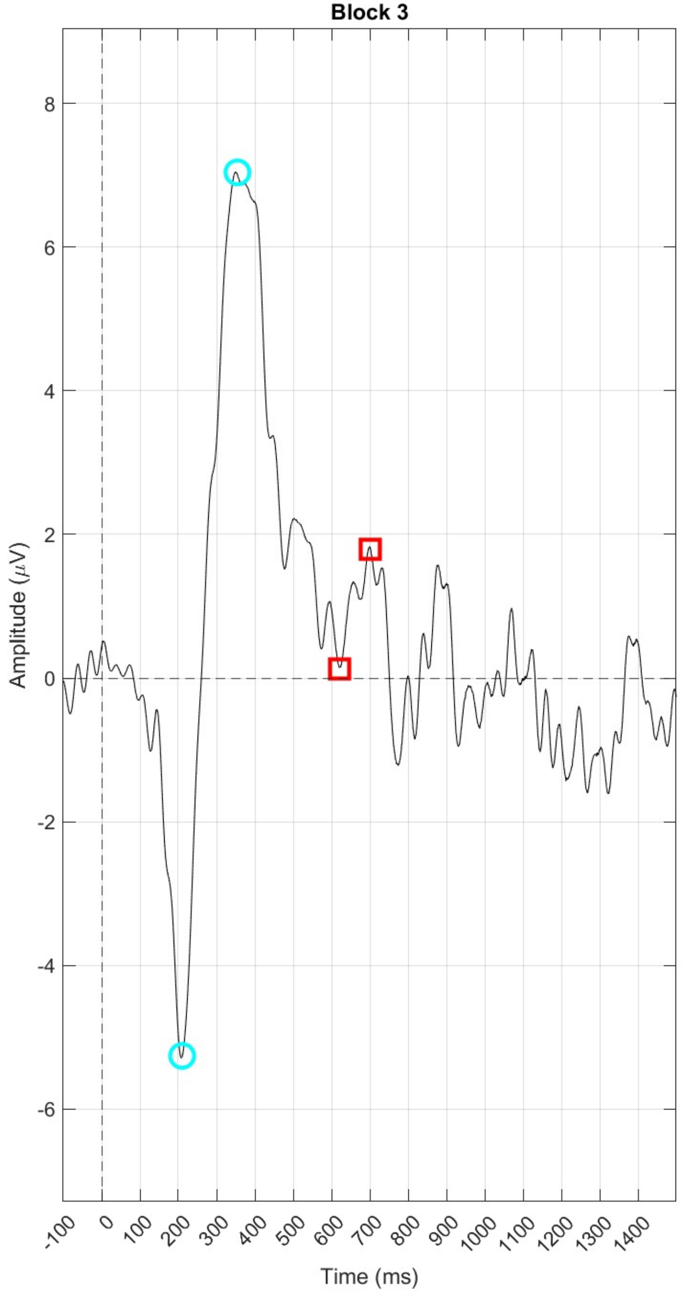
Block 3
8
6
4
2
0
-2
-4
-6
Amplitude (μV)
-100 0 100 200 300 400 500 600 700 800 900 1000 1100 1200 1300 1400
Time (ms)

Subject 43

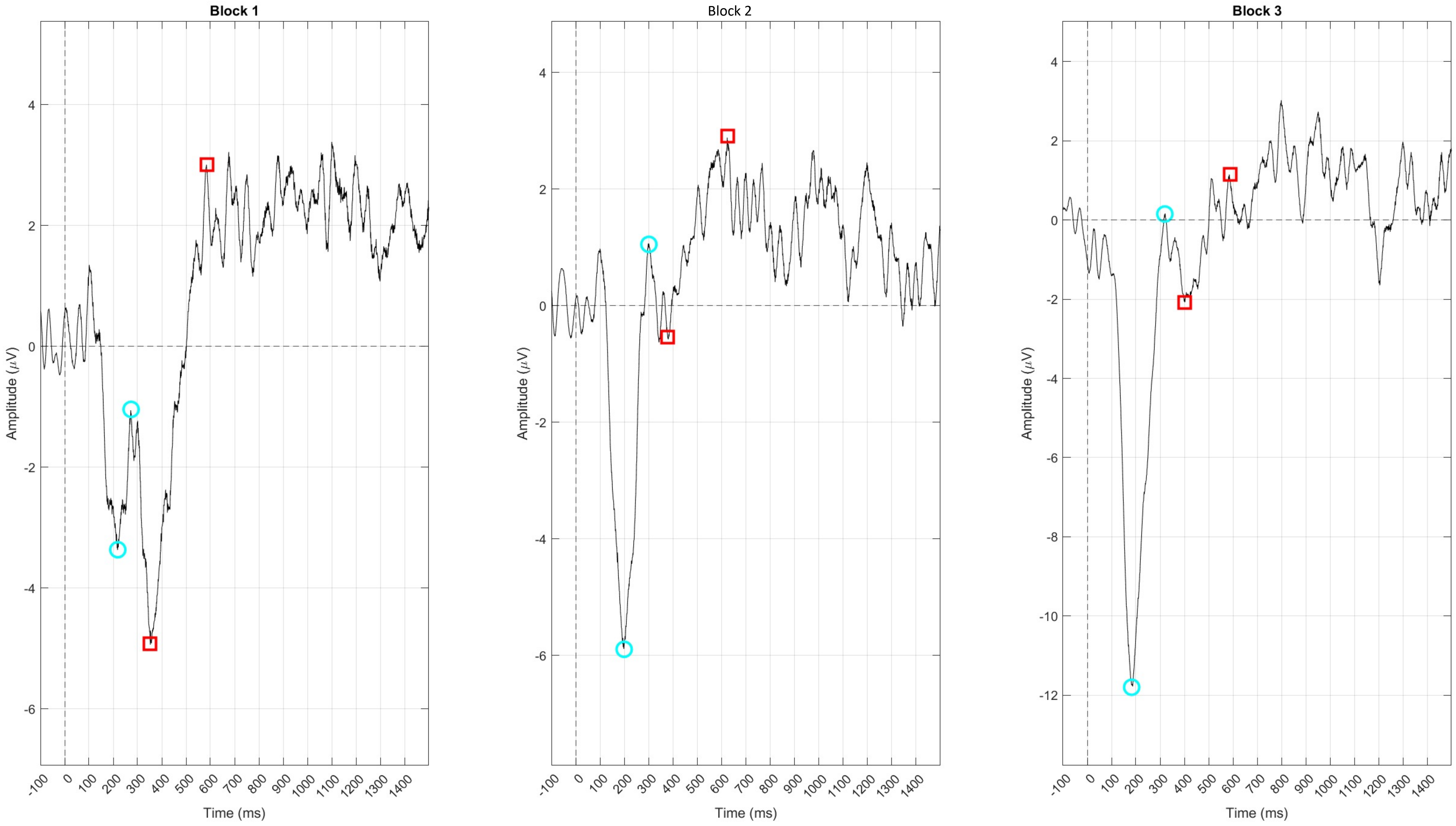

Subject 44

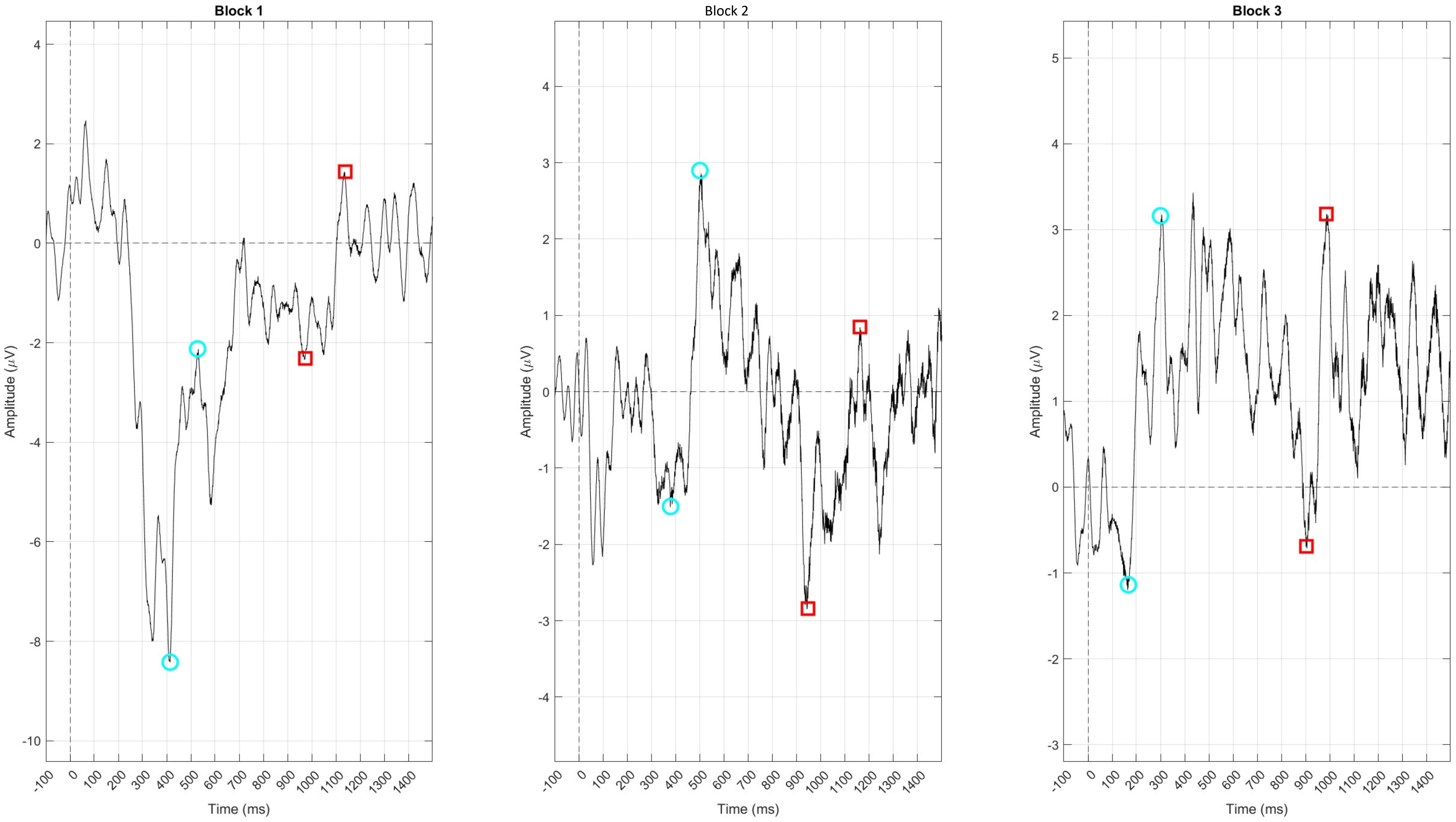

Block 1
Block 2
Block 3
Amplitude (μV)
Time (ms)
-100 0 100 200 300 400 500 600 700 800 900 1000 1100 1200 1300 1400
4 2 0 -2 -4 -6 -8 -10
4 3 2 1 0 -1 -2 -3 -4
5 4 3 2 1 0 -1 -2 -3

Subject 45

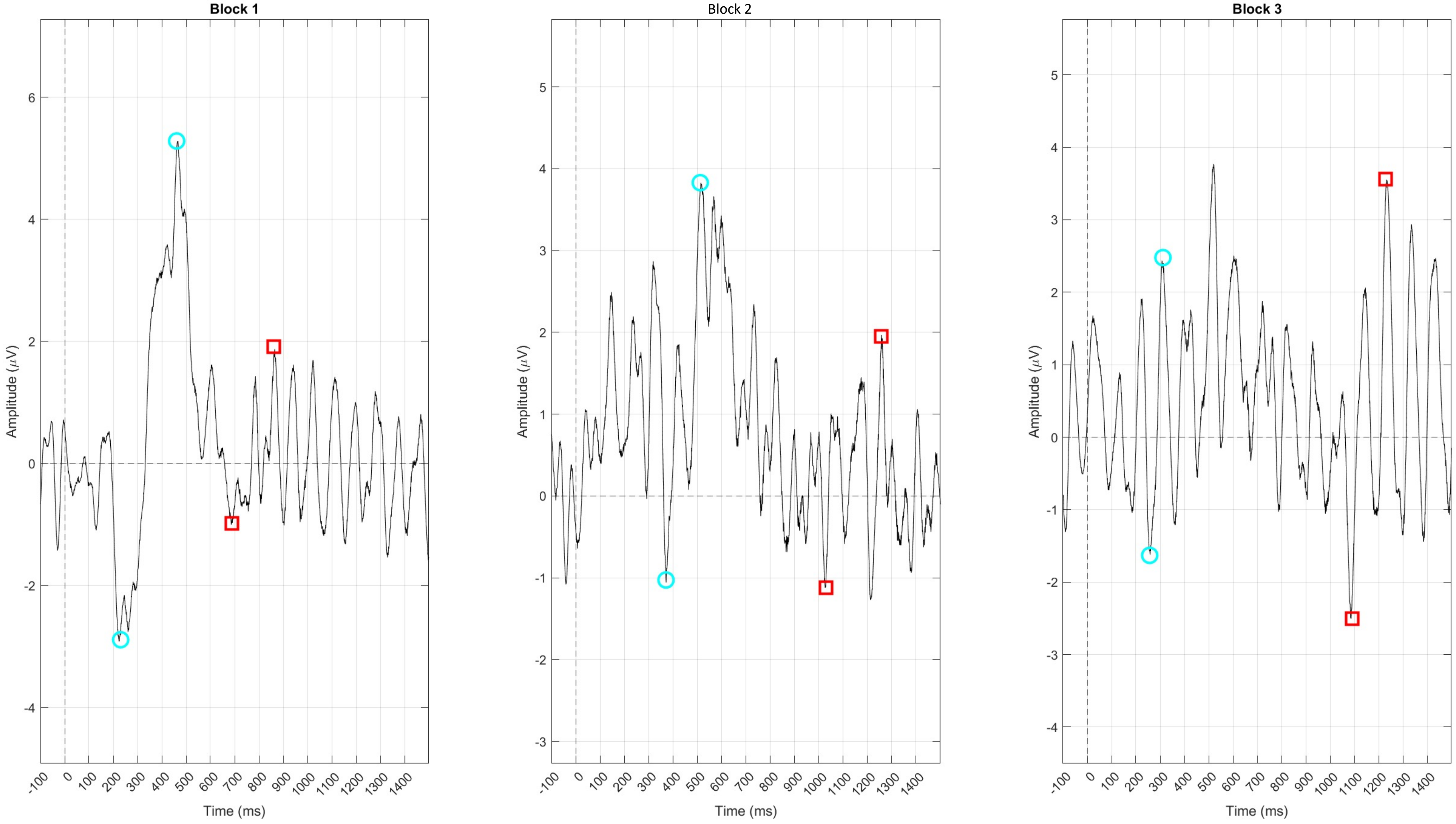

Subject 46

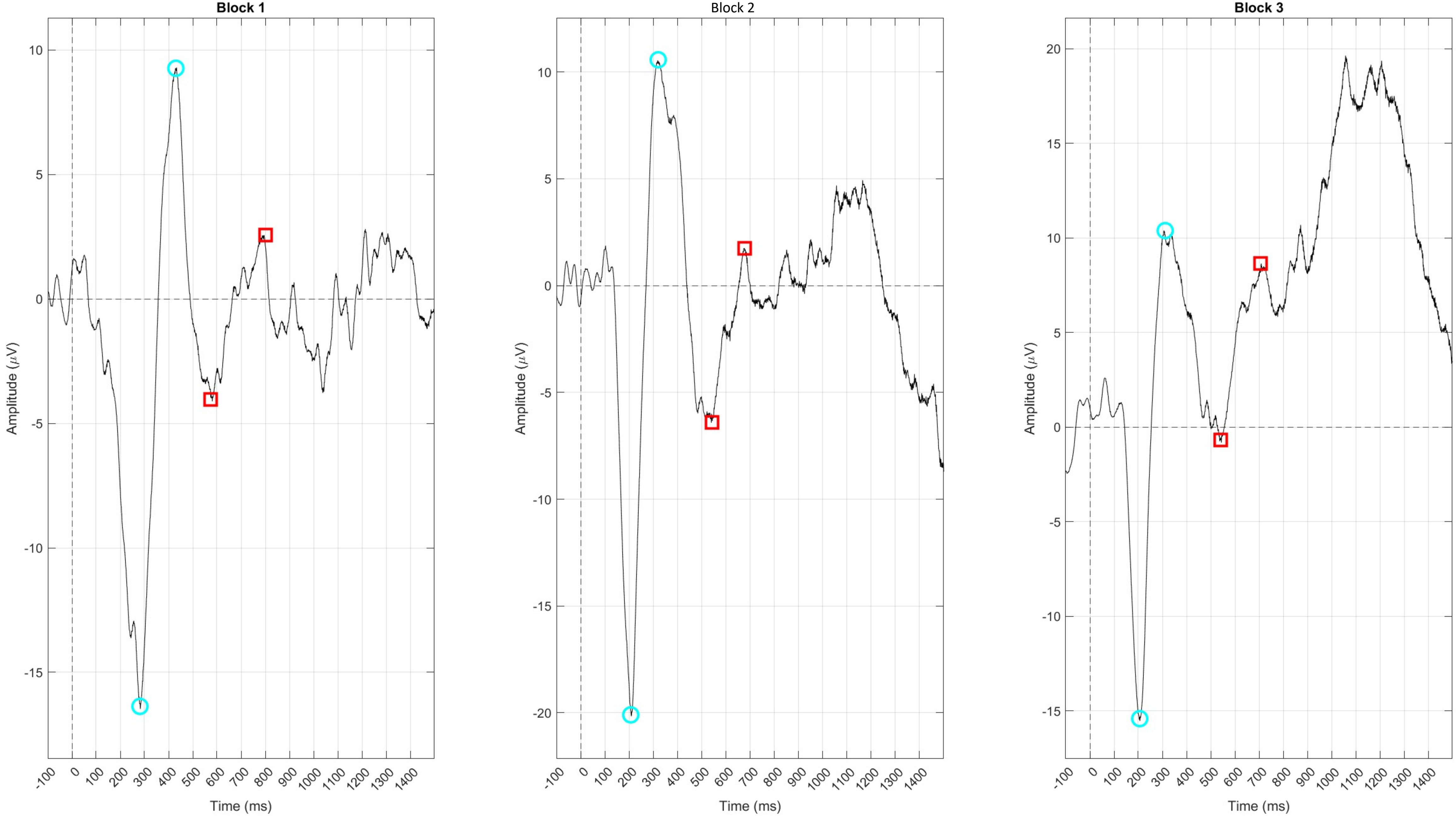

Block 1
Block 2
Block 3
Amplitude (μV)
Time (ms)
-100
0
100
200
300
400
500
600
700
800
900
1000
1100
1200
1300
1400
10
5
0
-5
-10
-15
-20
15
20

Subject 47

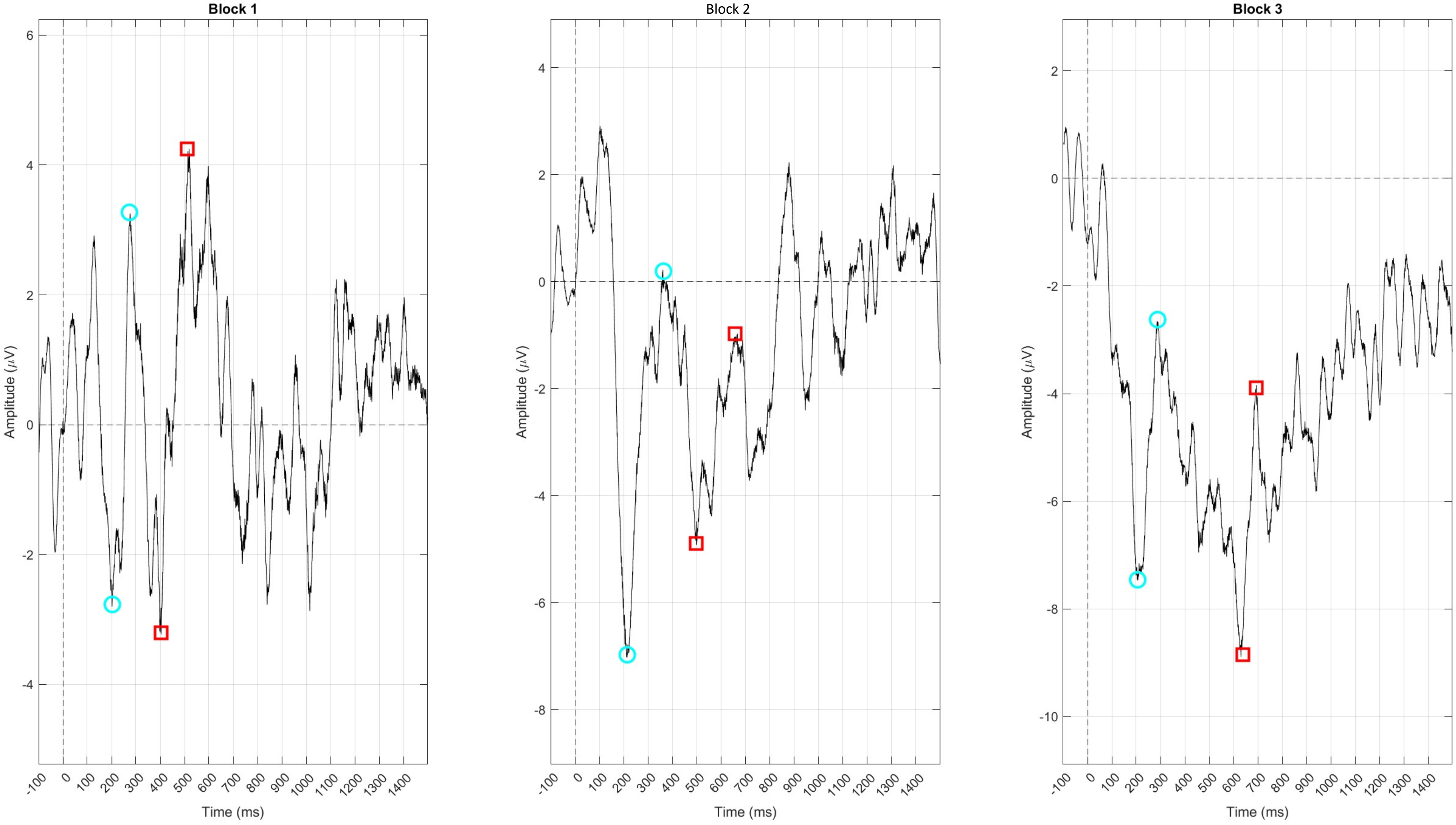
Block 1
Block 2
Block 3
Amplitude (μV)
Time (ms)
-100
0
100
200
300
400
500
600
700
800
900
1000
1100
1200
1300
1400

Subject 48

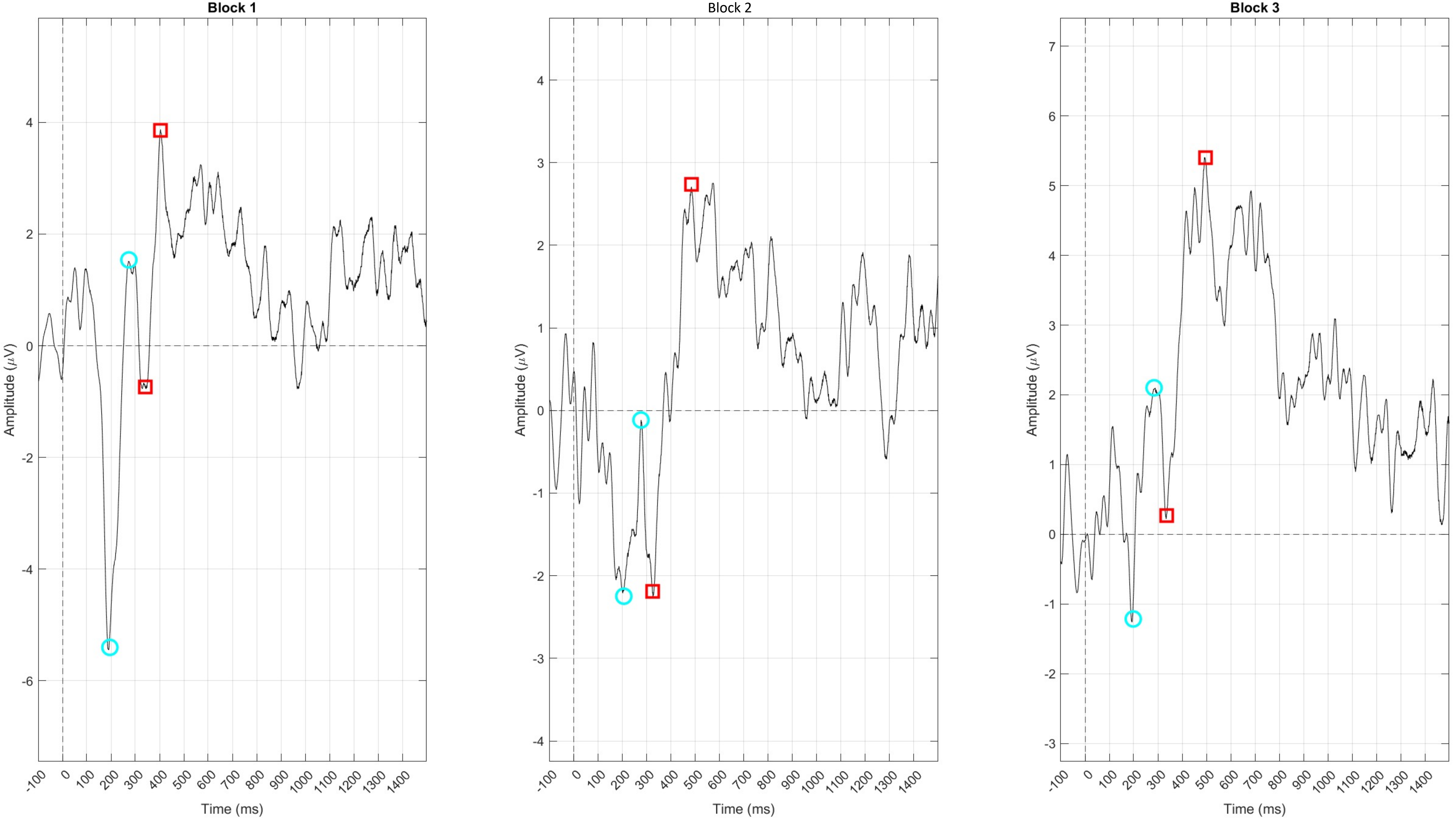

Block 1
Block 2
Block 3
Amplitude (μV)
Time (ms)
-100
0
100
200
300
400
500
600
700
800
900
1000
1100
1200
1300
1400

Subject 49

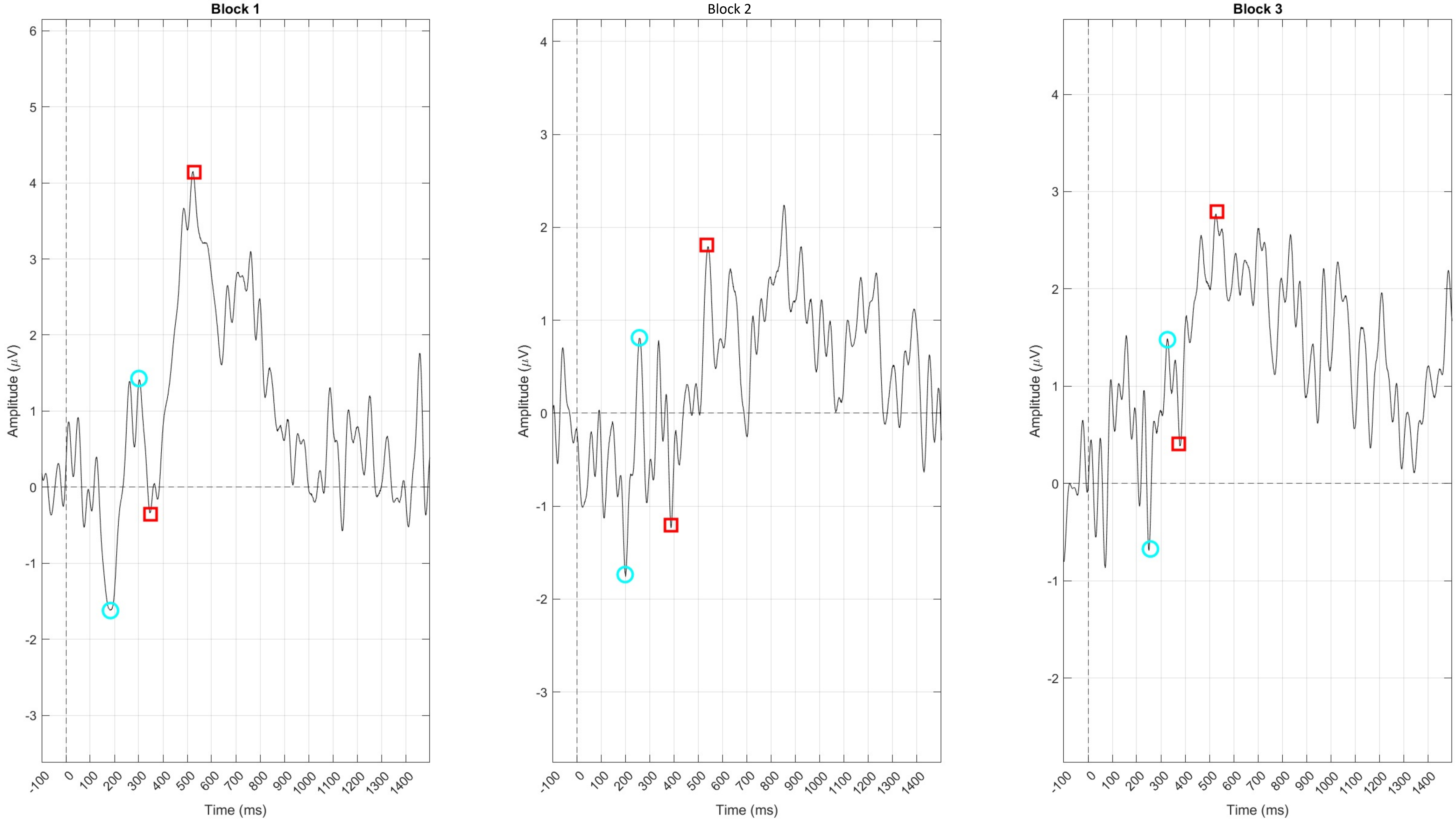

Block 1
Block 2
Block 3
Amplitude (μV)
Time (ms)
-100
0
100
200
300
400
500
600
700
800
900
1000
1100
1200
1300
1400

Subject 50

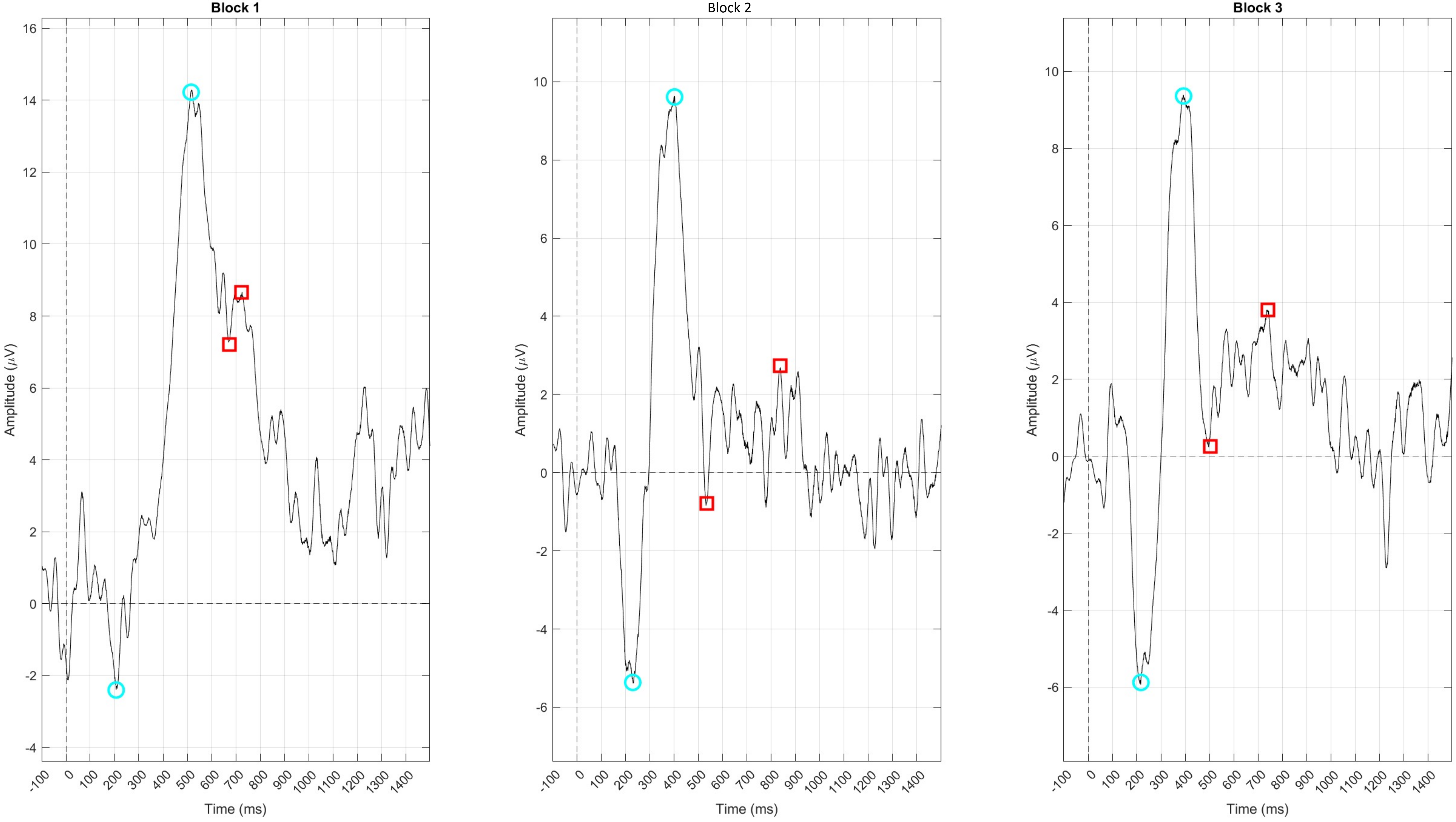

Subject 51

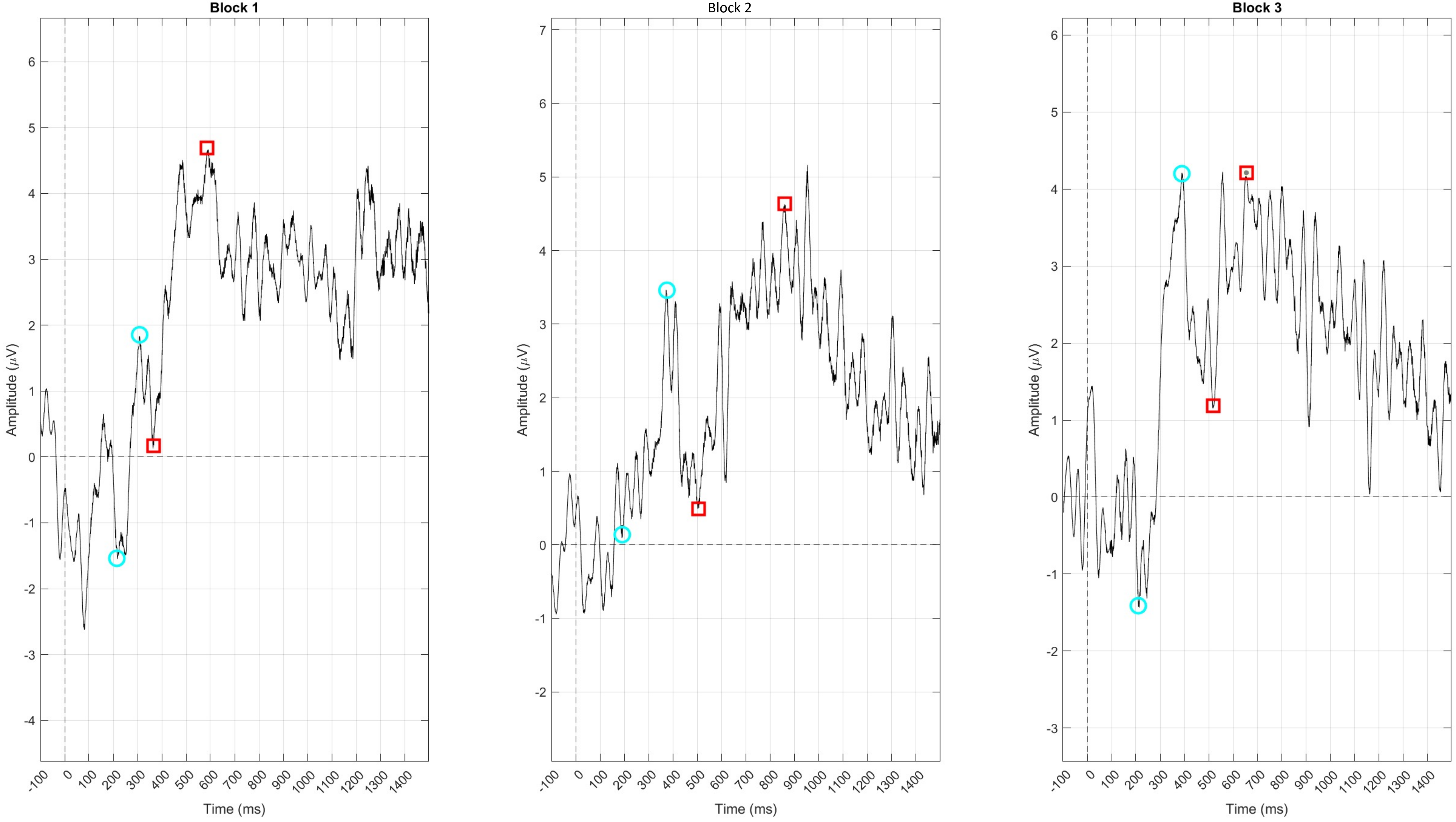
Block 1
Block 2
Block 3
Amplitude (μV)
Time (ms)
-100
0
100
200
300
400
500
600
700
800
900
1000
1100
1200
1300
1400

Subject 52

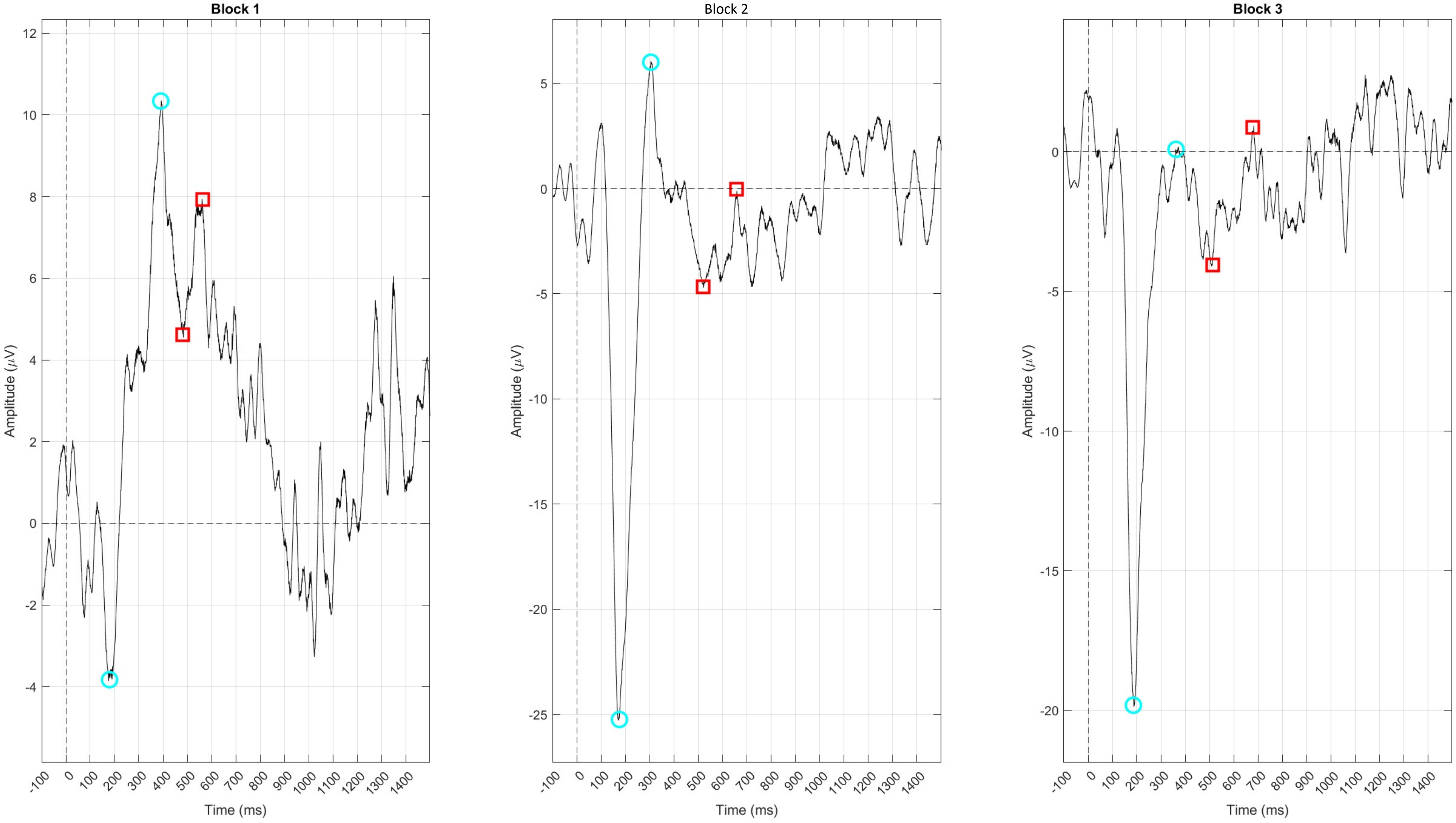

Subject 53

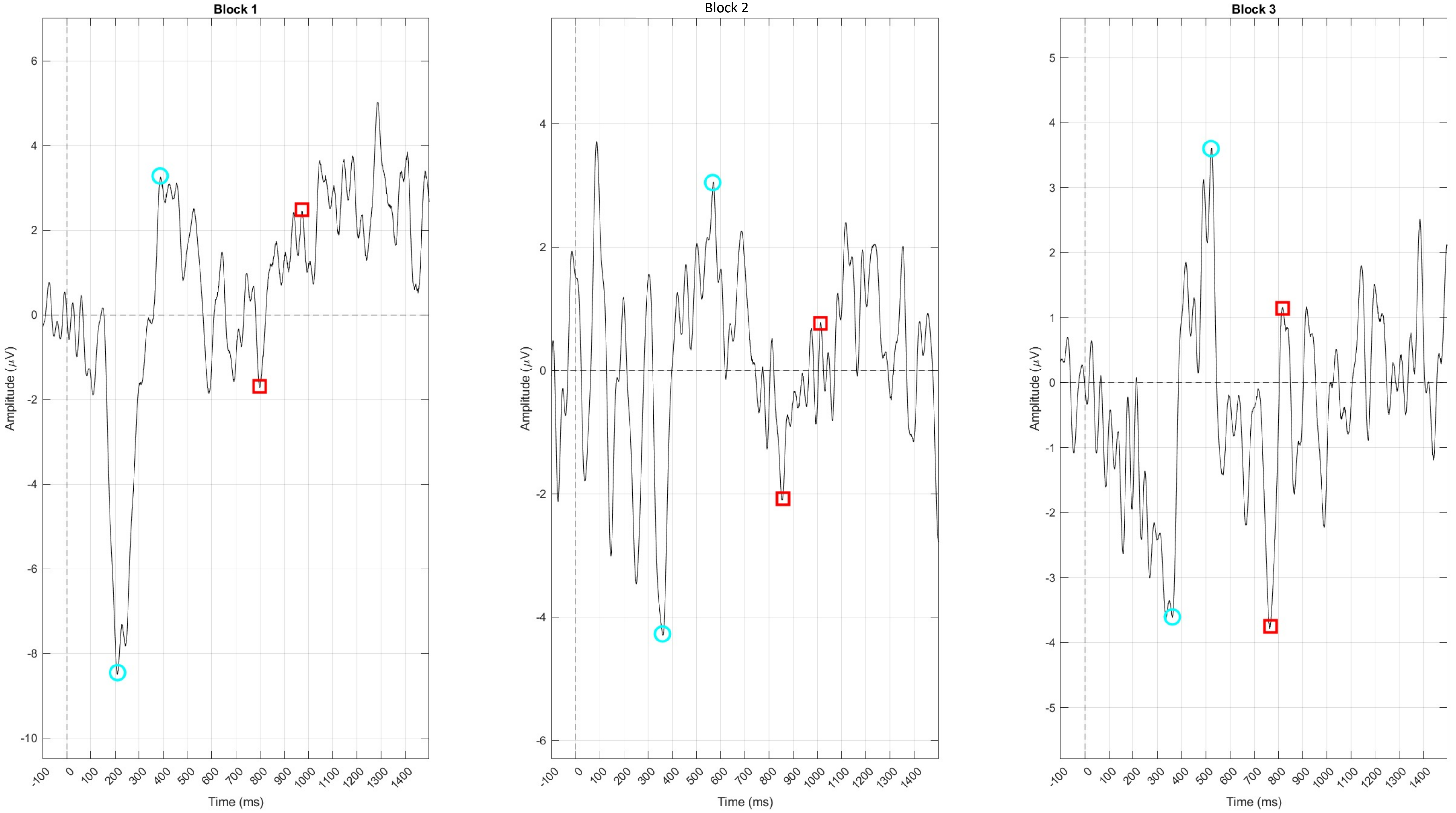
Block 1
Block 2
Block 3
Amplitude (μV)
Time (ms)
6
4
2
0
-2
-4
-6
-8
-10
5
3
1
-1
-3
-5
-100
100
200
300
400
500
600
700
800
900
1000
1100
1200
1300
1400

Subject 54

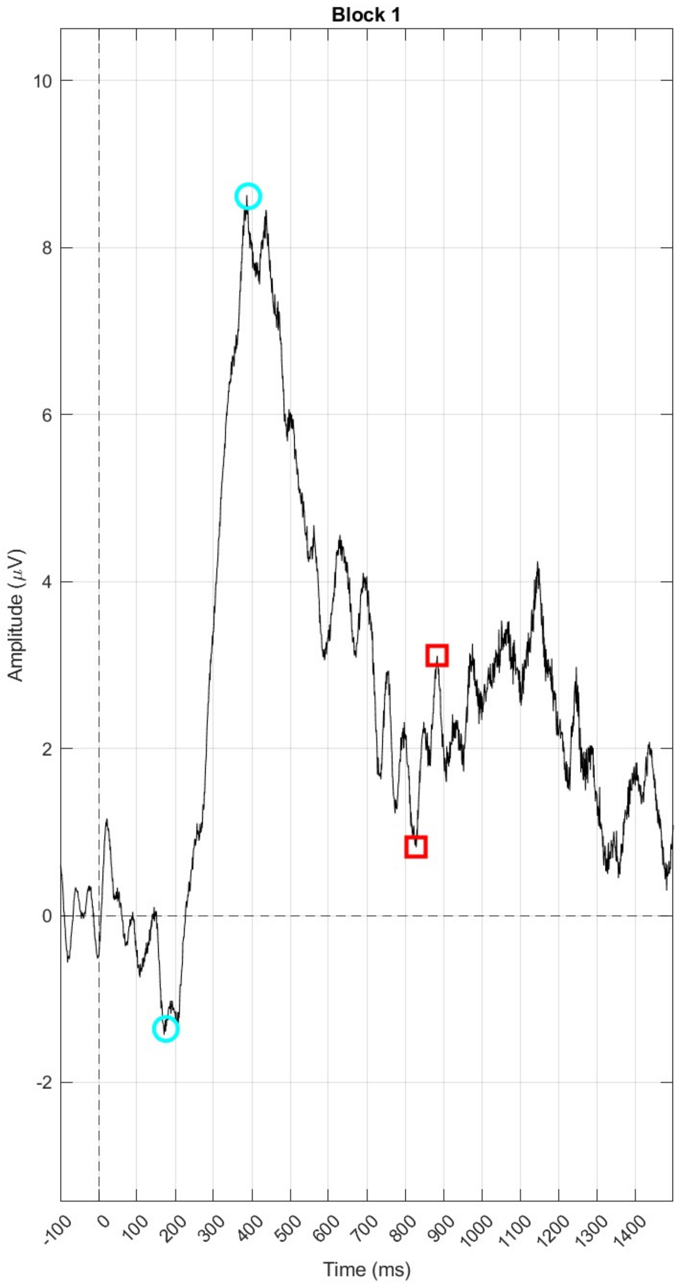

Block 1
Amplitude (μV)
Time (ms)

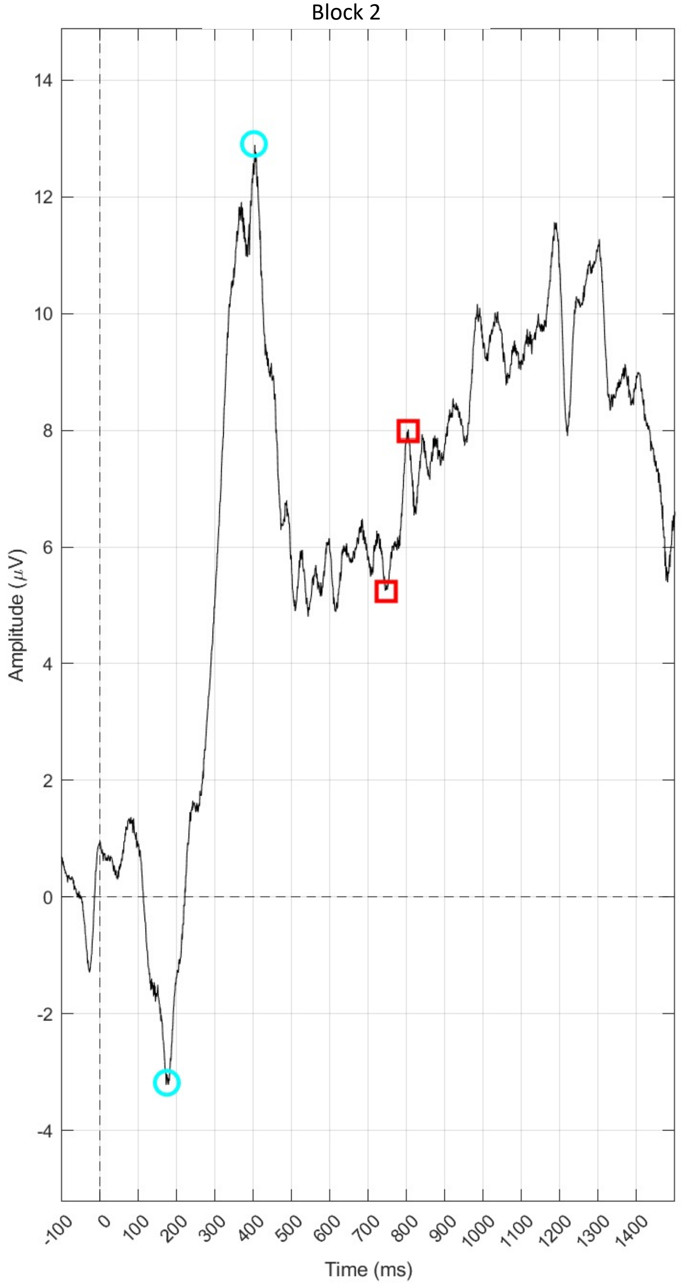

Block 2
Amplitude (μV)
Time (ms)

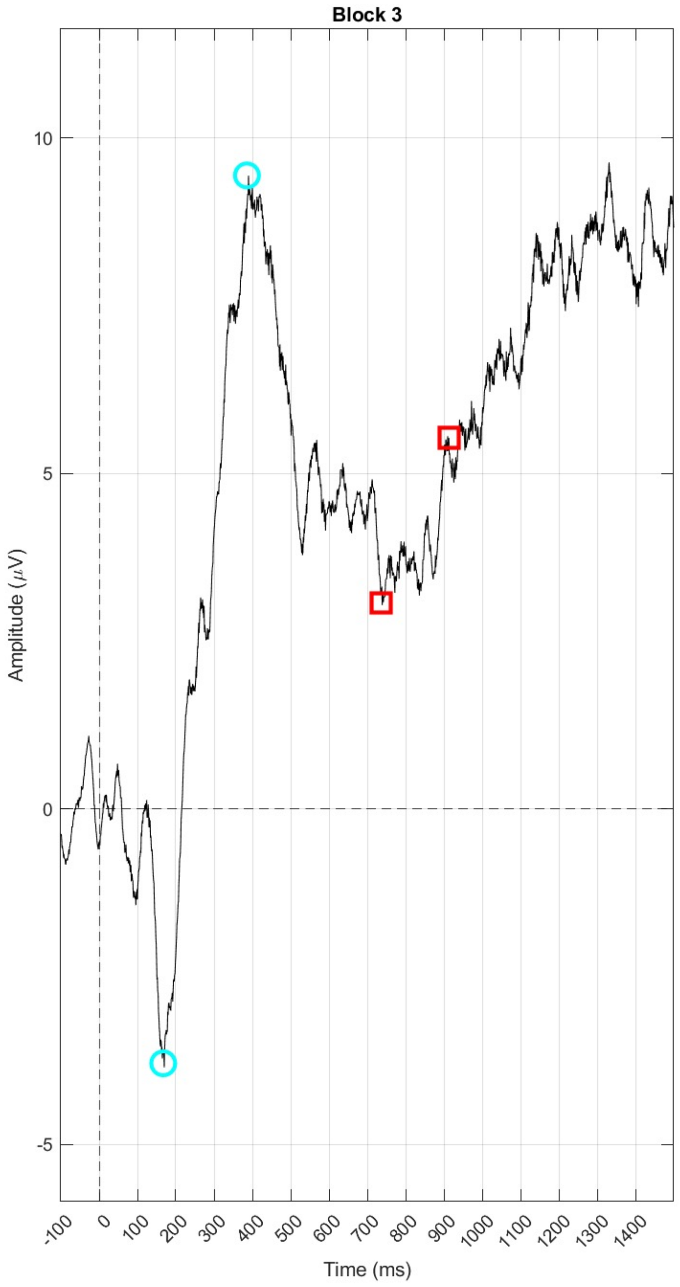

Block 3
Amplitude (μV)
Time (ms)

Subject 55

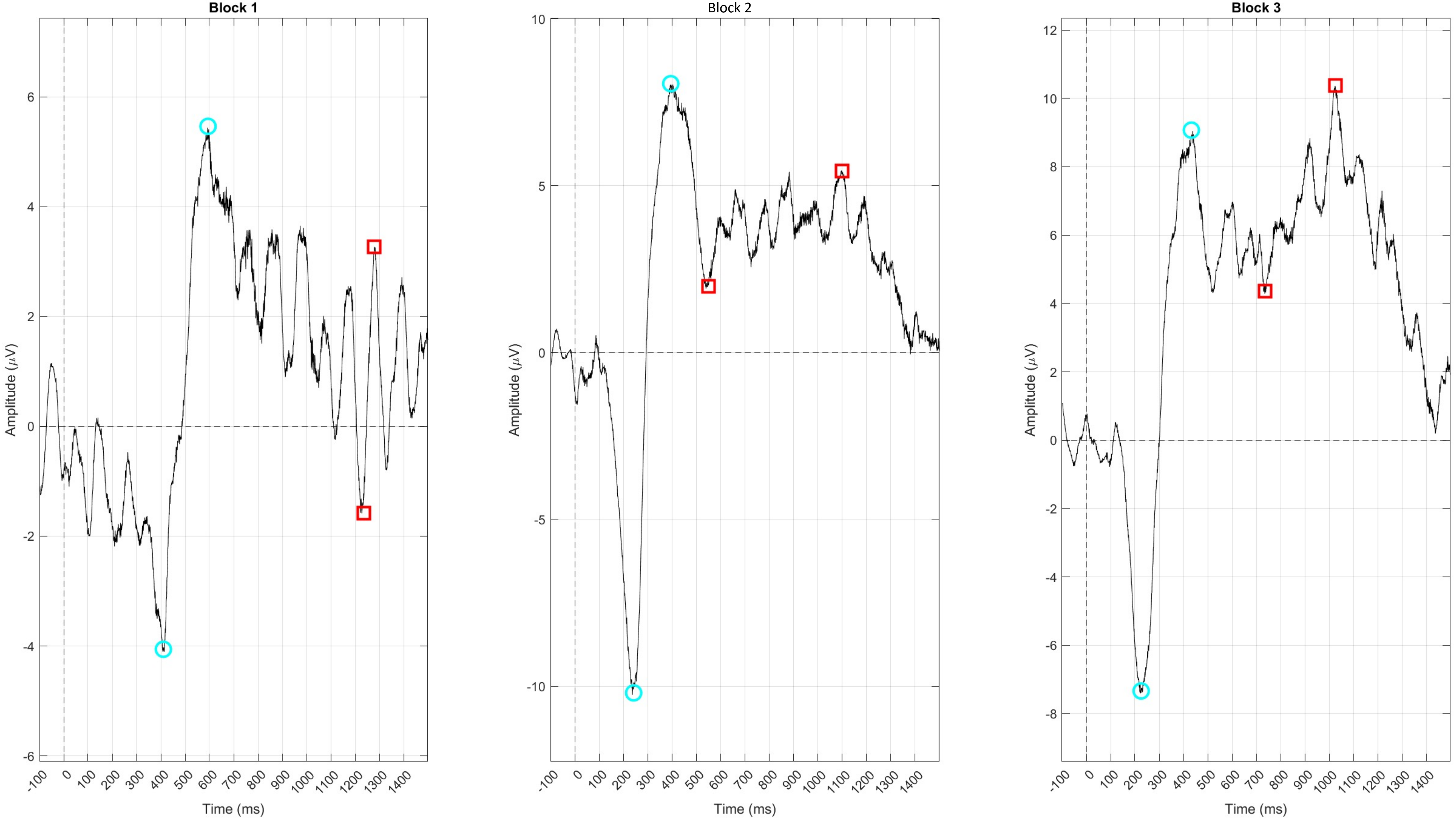

Block 1
Block 2
Block 3
Amplitude (μV)
Time (ms)
-100
0
100
200
300
400
500
600
700
800
900
1000
1100
1200
1300
1400

Subject 56

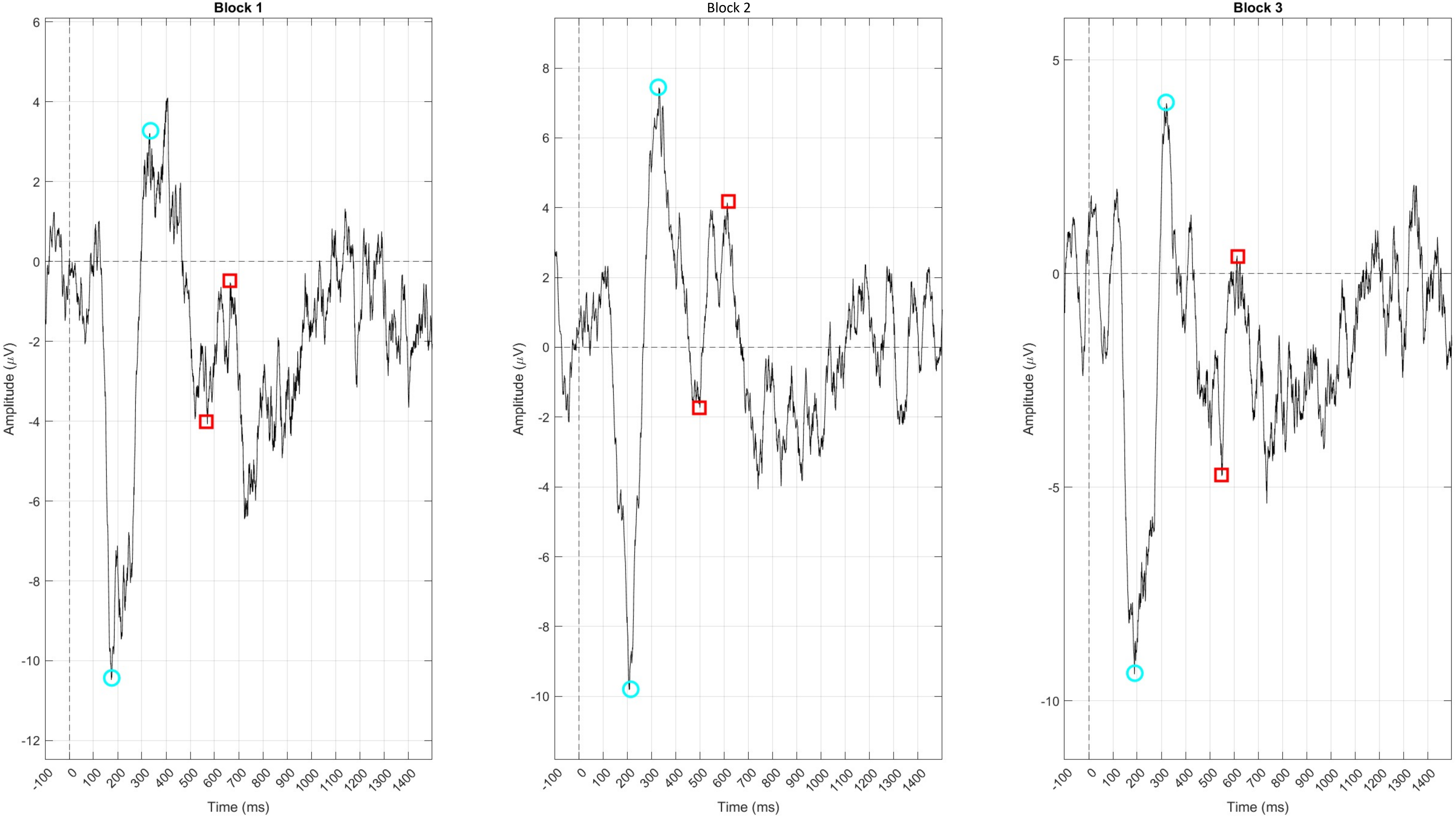

Block 1
Block 2
Block 3
Amplitude (μV)
Time (ms)
-100
0
100
200
300
400
500
600
700
800
900
1000
1100
1200
1300
1400

Subject 57

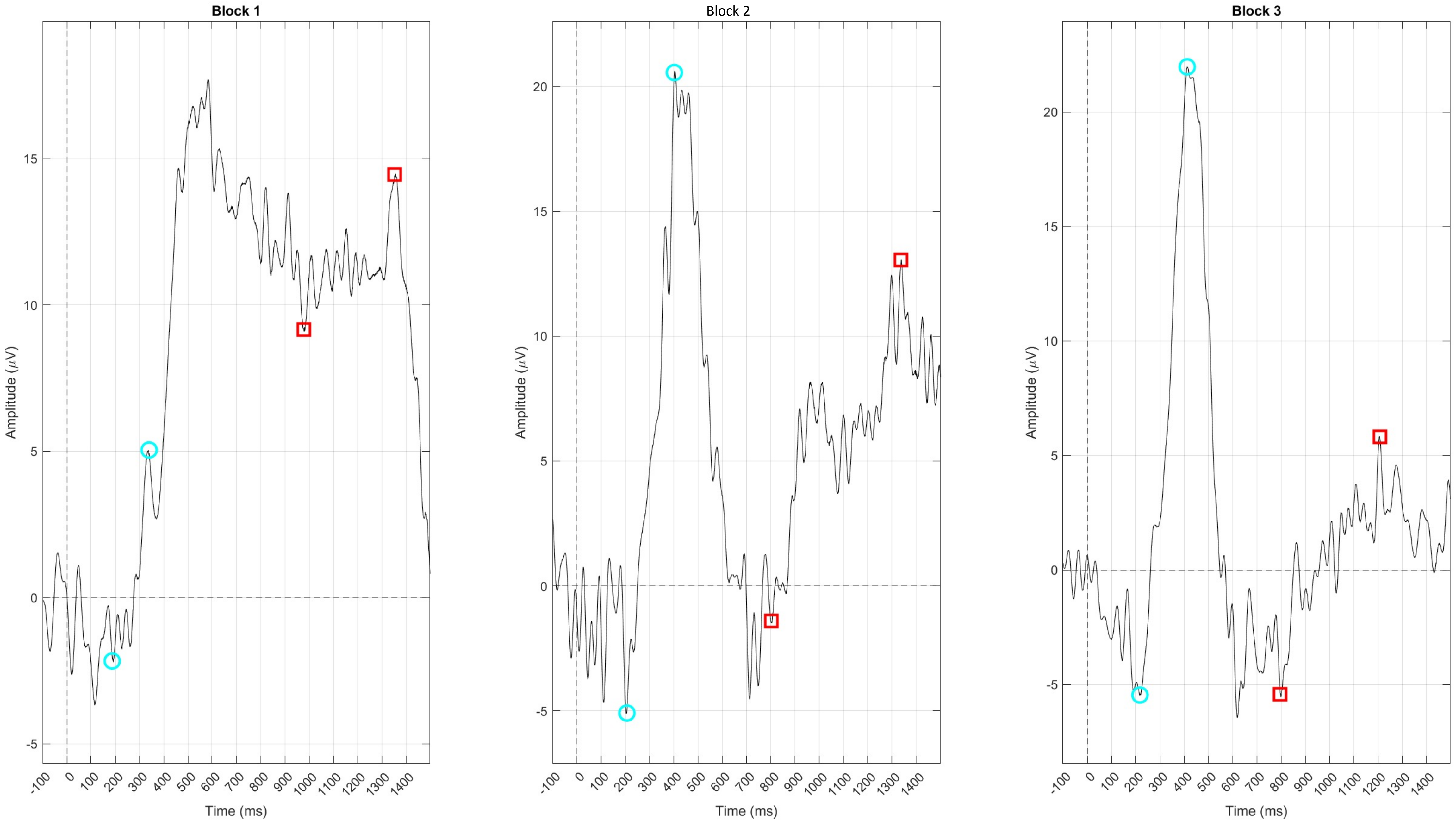
Block 1
Block 2
Block 3
Amplitude (μV)
Time (ms)
-100
0
100
200
300
400
500
600
700
800
900
1000
1100
1200
1300
1400
-5
0
5
10
15
20

Subject 58

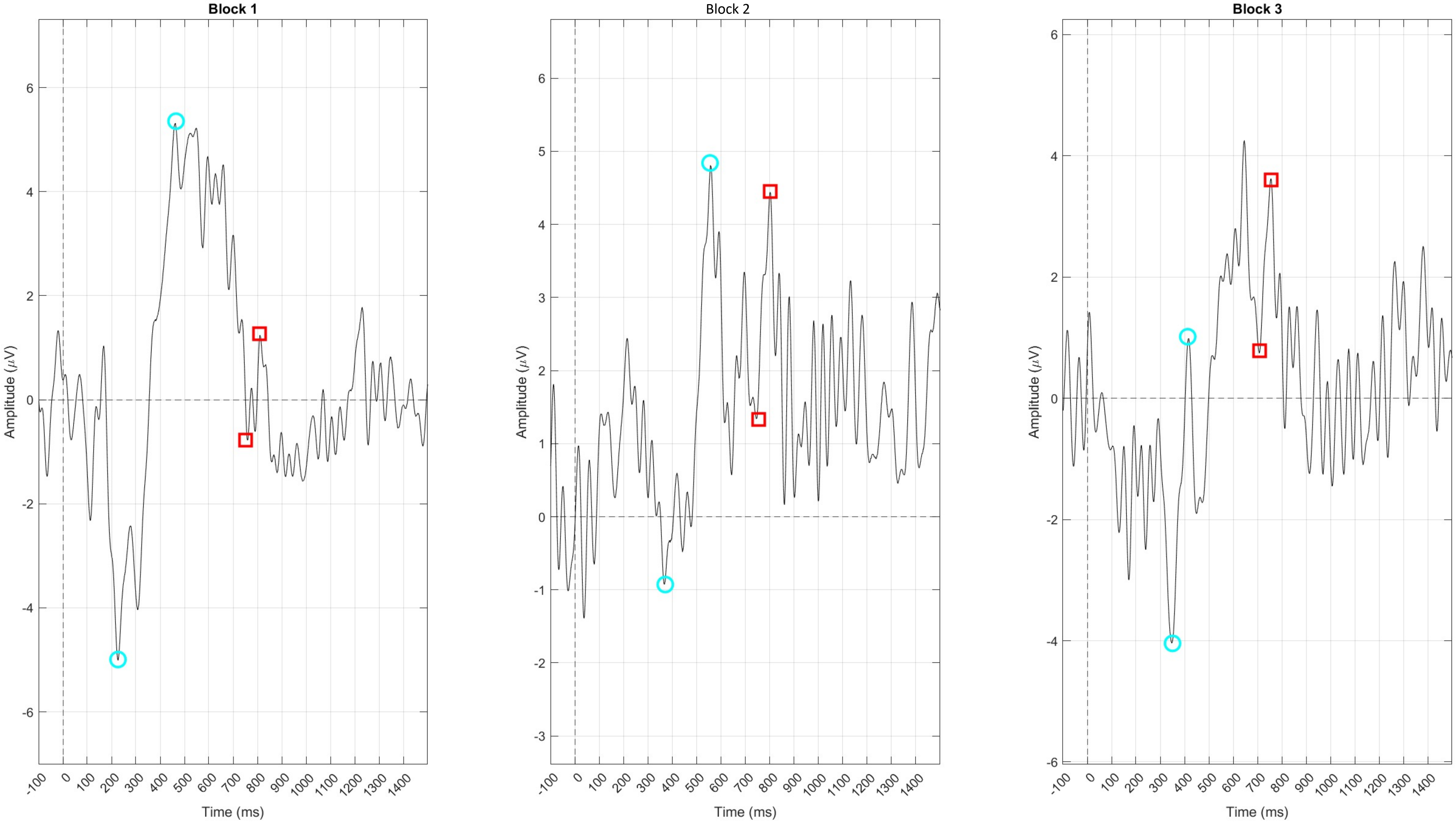

Block 1
Block 2
Block 3
Amplitude (μV)
Time (ms)
-100
0
100
200
300
400
500
600
700
800
900
1000
1100
1200
1300
1400

Subject 59

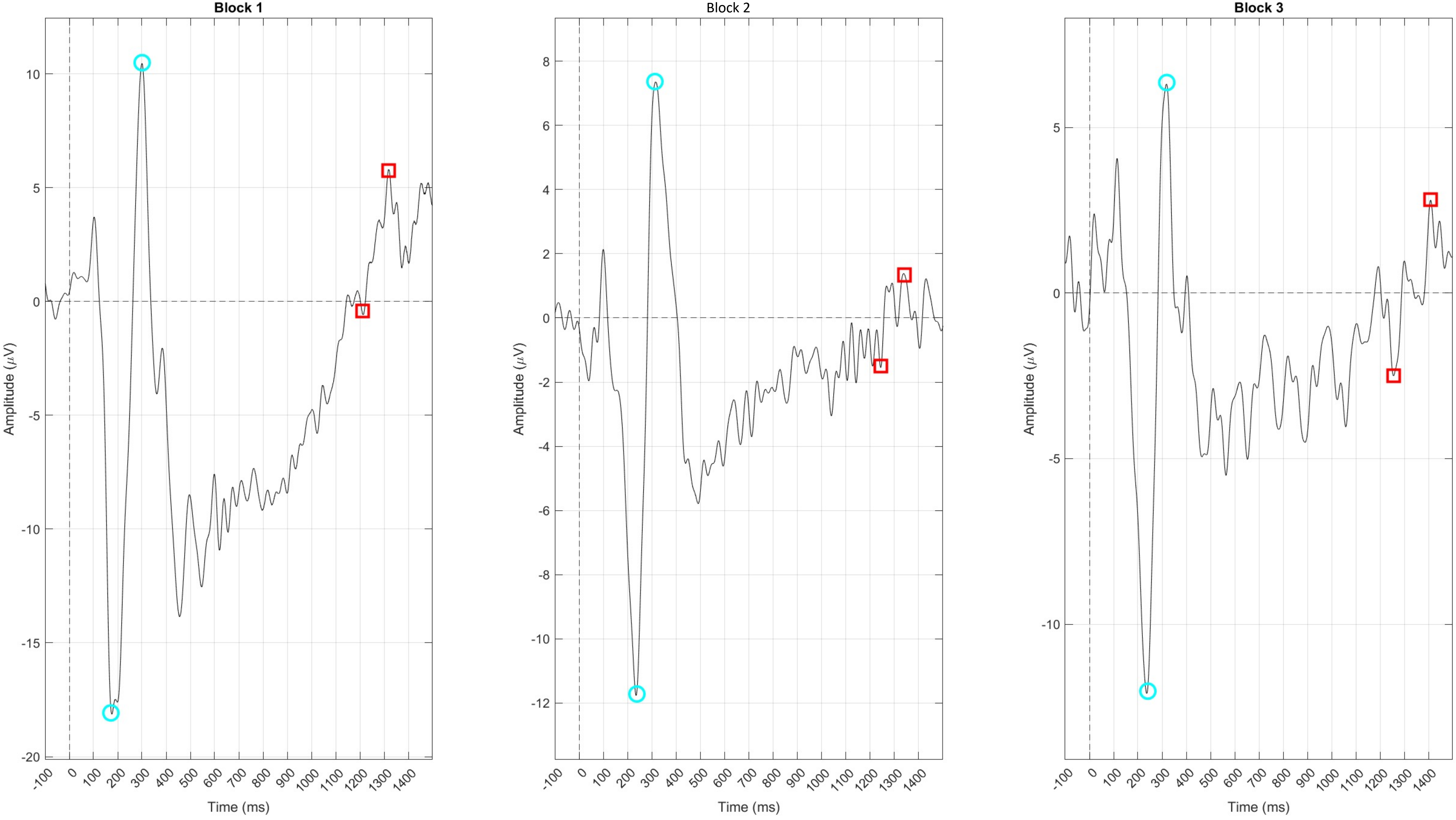

Block 1
Block 2
Block 3
Amplitude (μV)
Time (ms)
-100
0
100
200
300
400
500
600
700
800
900
1000
1100
1200
1300
1400
10
5
0
-5
-10
-15
-20
8
6
4
2
-2
-4
-6
-8
-12

Subject 60

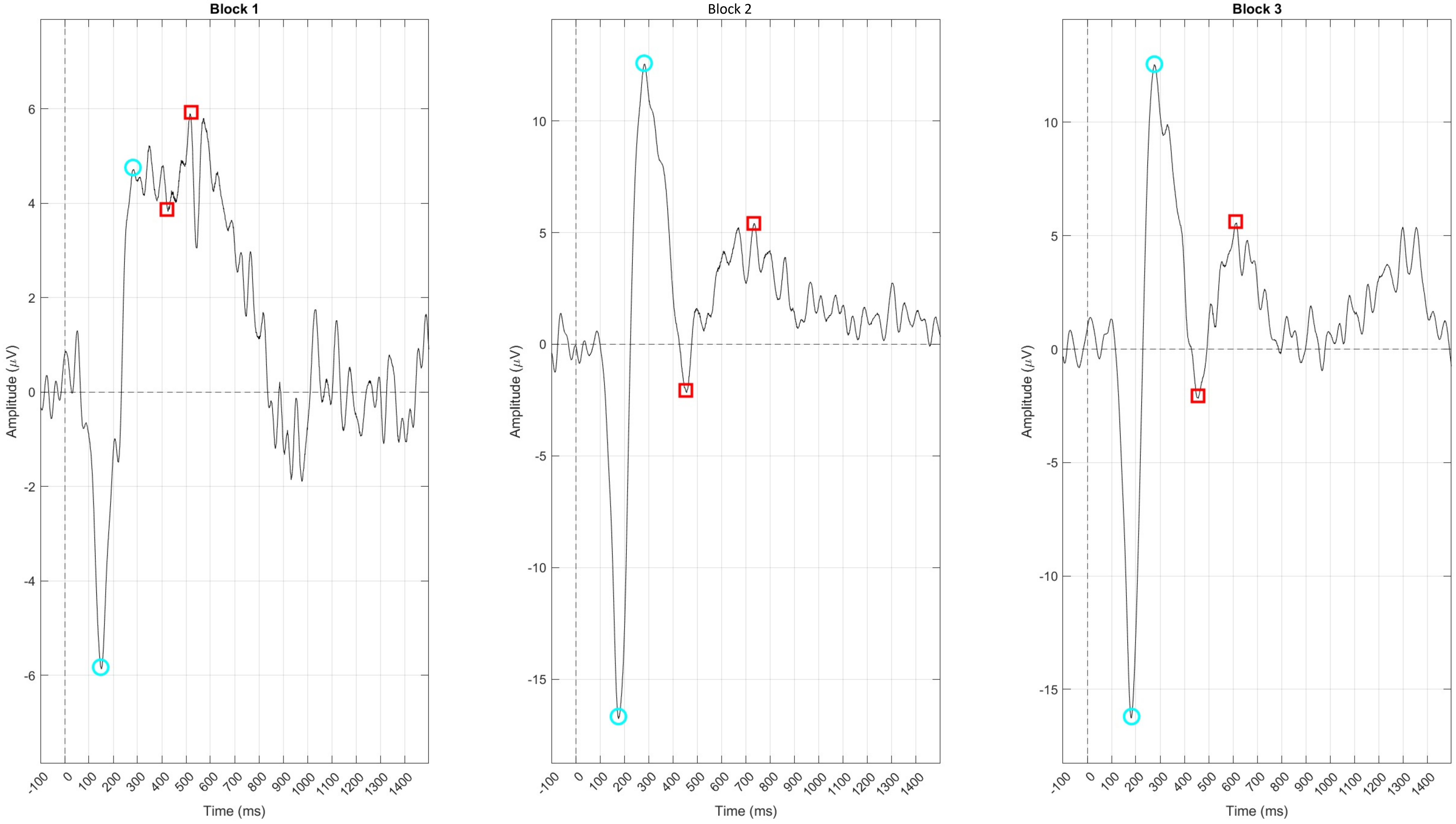

Block 1
Block 2
Block 3
Amplitude (μV)
Time (ms)
-100
0
100
200
300
400
500
600
700
800
900
1000
1100
1200
1300
1400

Subject 61

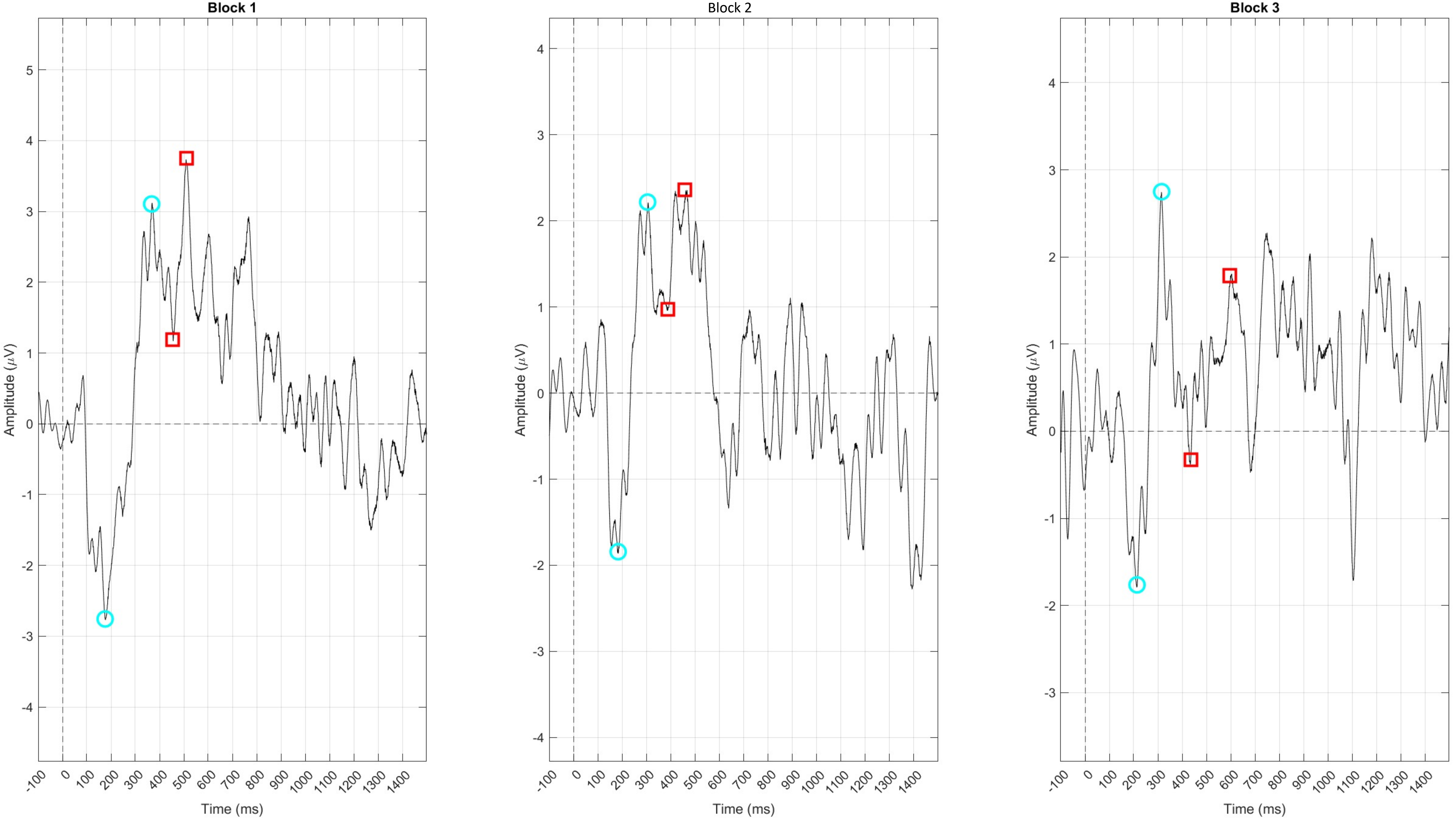
Block 1
Block 2
Block 3
Amplitude (μV)
Time (ms)
-100 0 100 200 300 400 500 600 700 800 900 1000 1100 1200 1300 1400

Subject 62

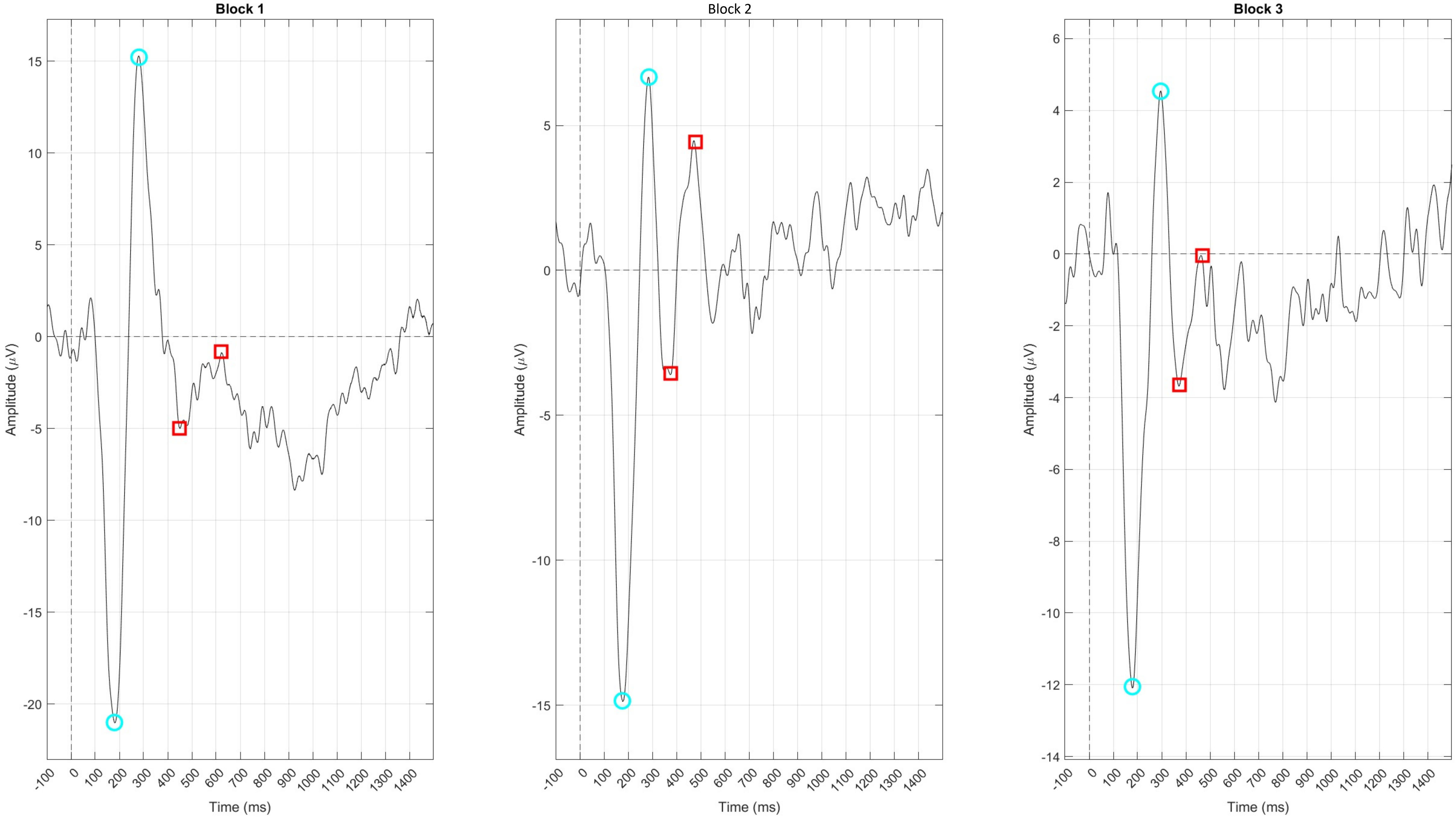
Block 1
Block 2
Block 3
Amplitude (μV)
Time (ms)
-100
0
100
200
300
400
500
600
700
800
900
1000
1100
1200
1300
1400
15
10
5
0
-5
-10
-15
-20
6
4
2
-2
-4
-6
-8
-12
-14

Subject 63

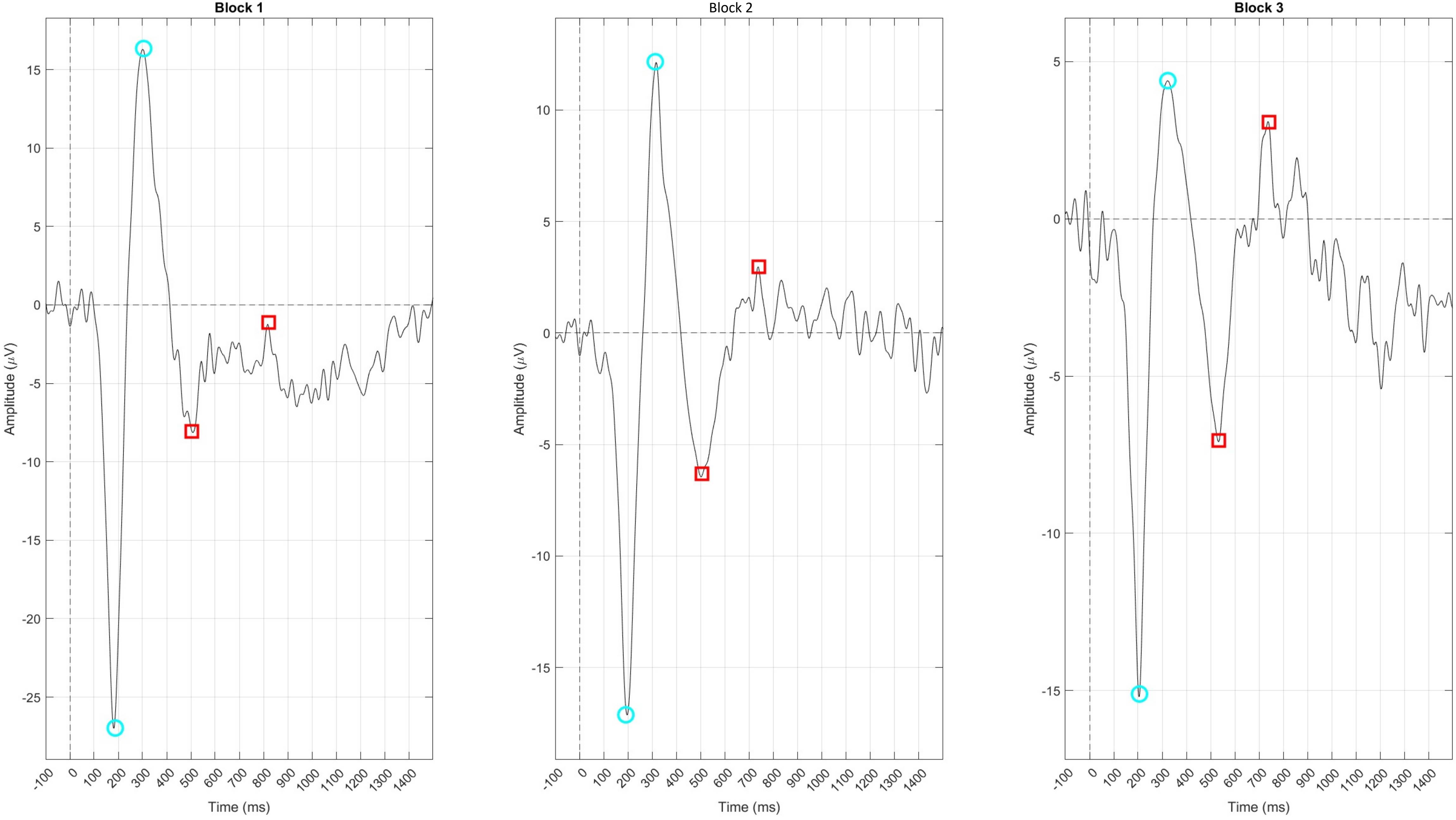

Subject 64

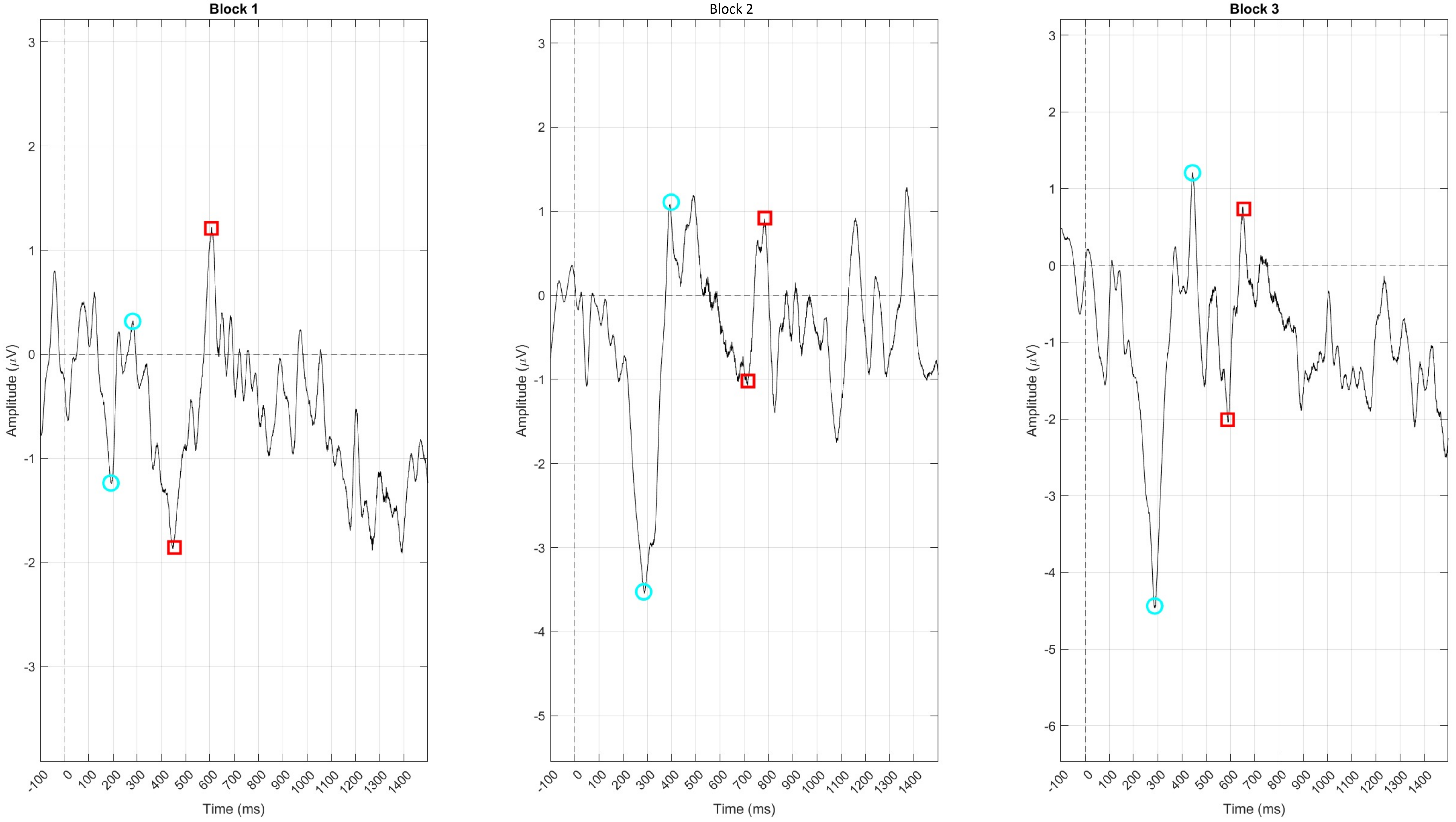

Block 1
Block 2
Block 3
Amplitude (μV)
Time (ms)
-100
0
100
200
300
400
500
600
700
800
900
1000
1100
1200
1300
1400

Subject 65

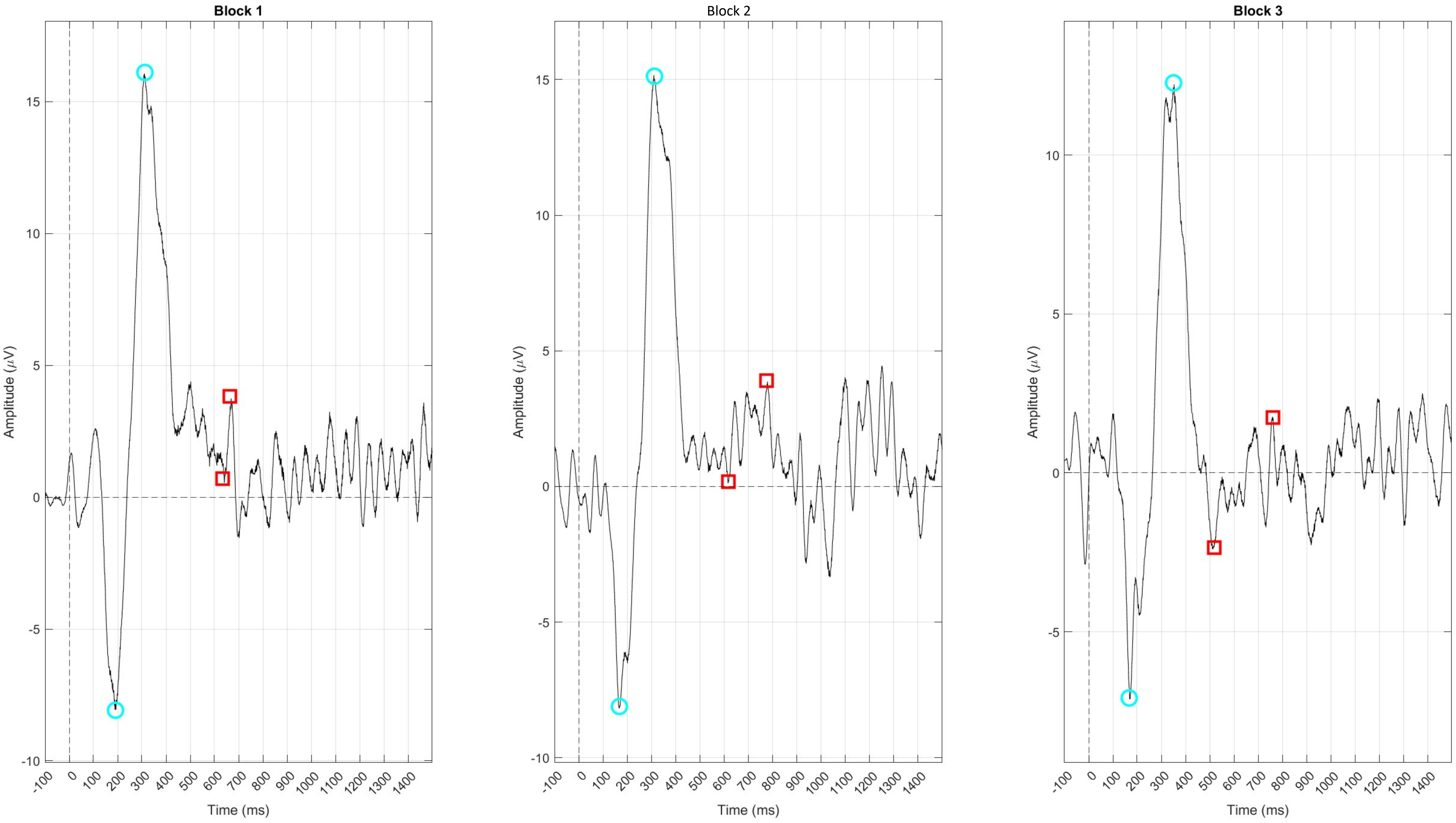

Block 1
Block 2
Block 3
Amplitude (μV)
Time (ms)
-10
-5
0
5
10
15
-100
0
100
200
300
400
500
600
700
800
900
1000
1100
1200
1300
1400

Subject 66

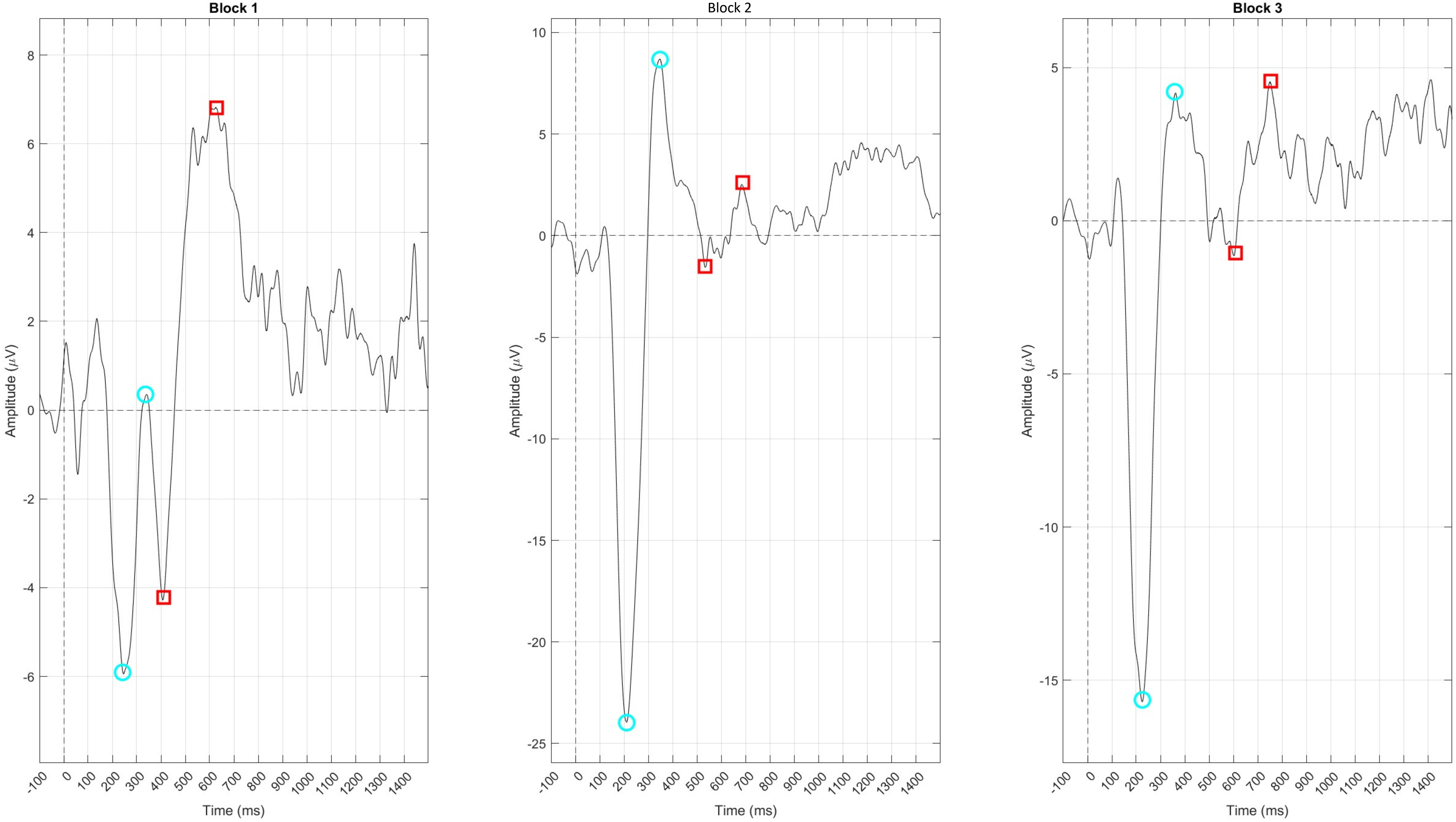

Subject 67

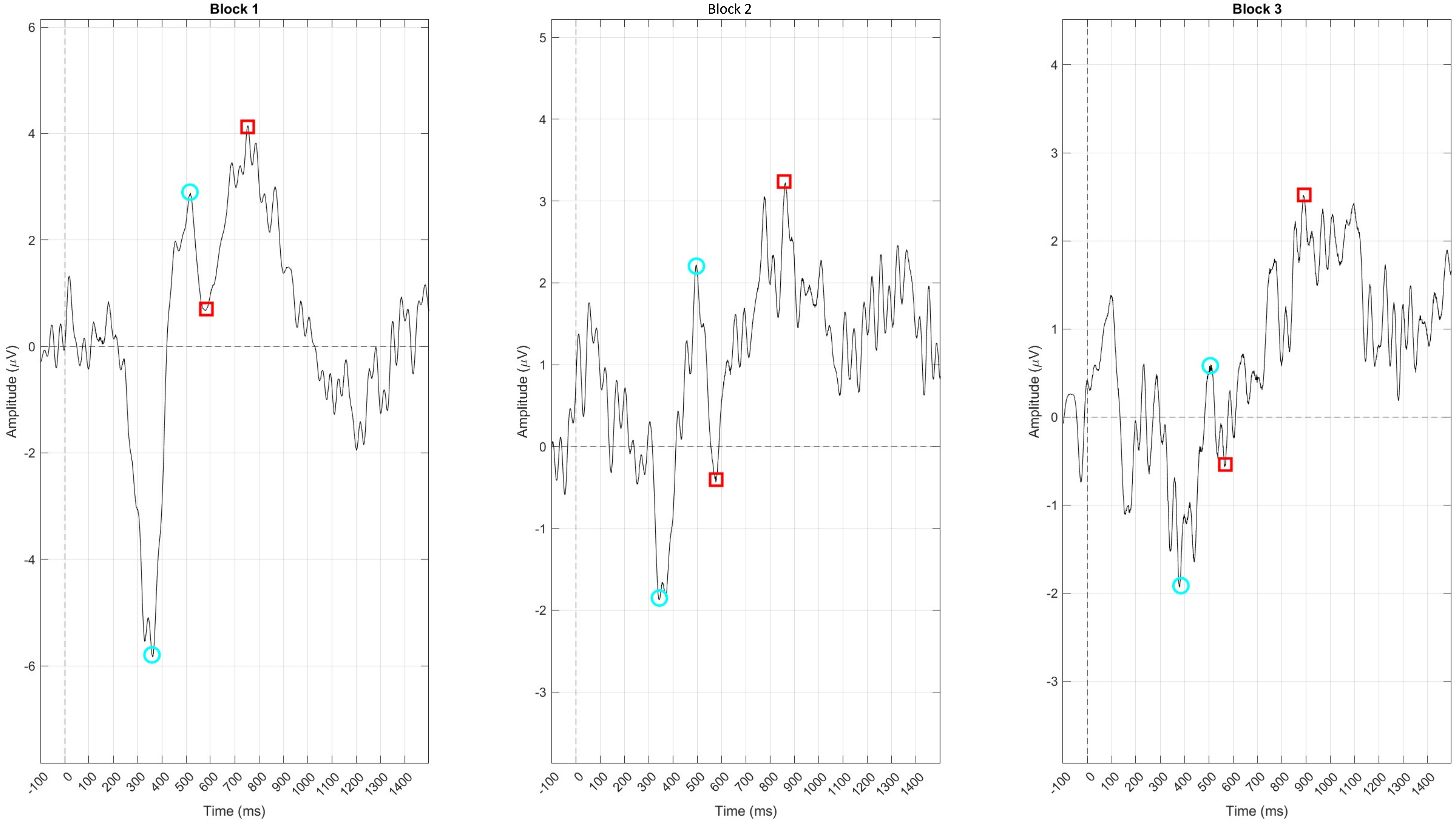

Block 1
Block 2
Block 3
Amplitude (μV)
Time (ms)
-100
0
100
200
300
400
500
600
700
800
900
1000
1100
1200
1300
1400

Subject 68

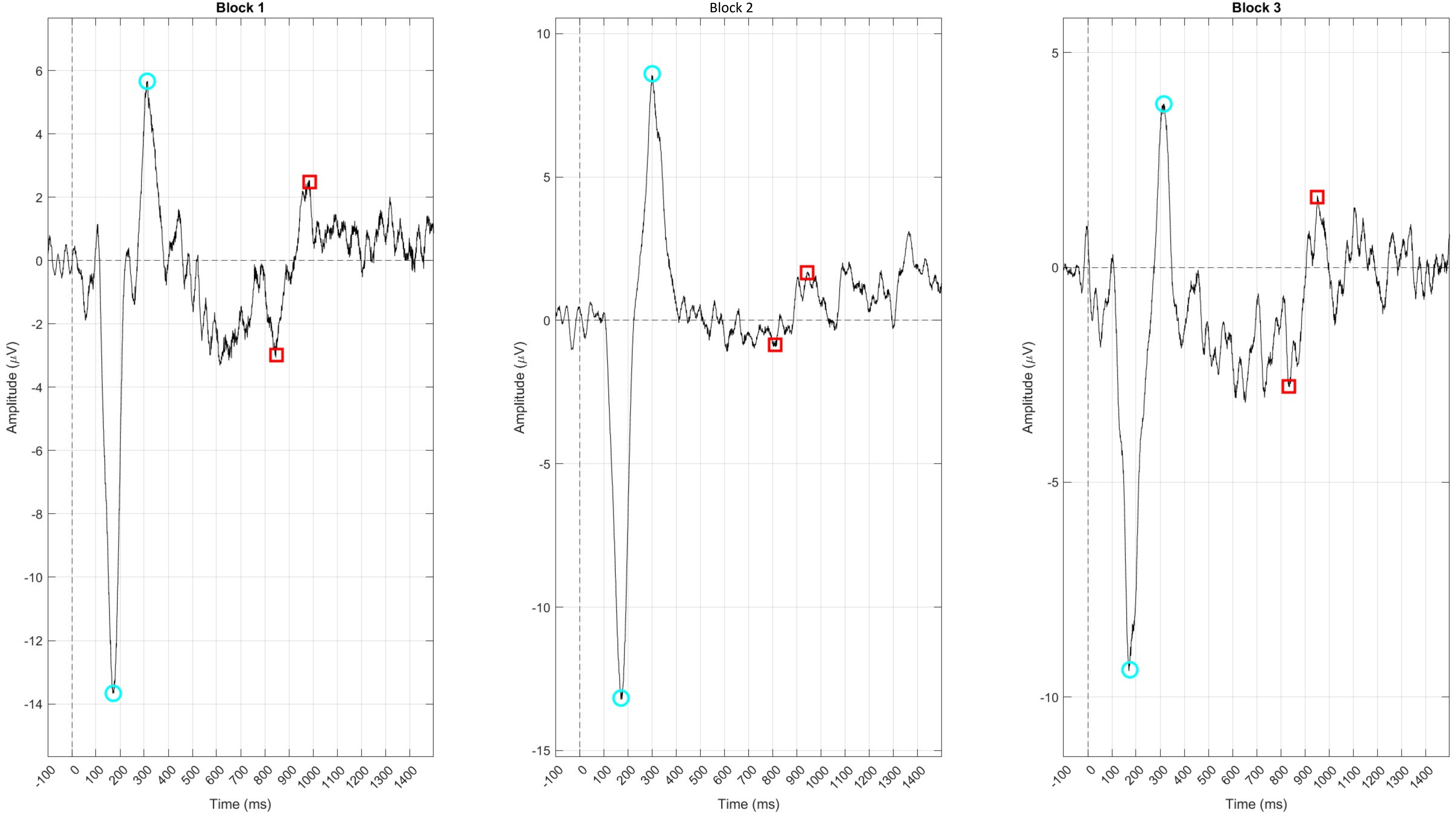
Block 1
Block 2
Block 3
Amplitude (μV)
Time (ms)
-100
0
100
200
300
400
500
600
700
800
900
1000
1100
1200
1300
1400
6
4
2
0
-2
-4
-6
-8
-10
-12
-14
10
5
-5
-15

Subject 69

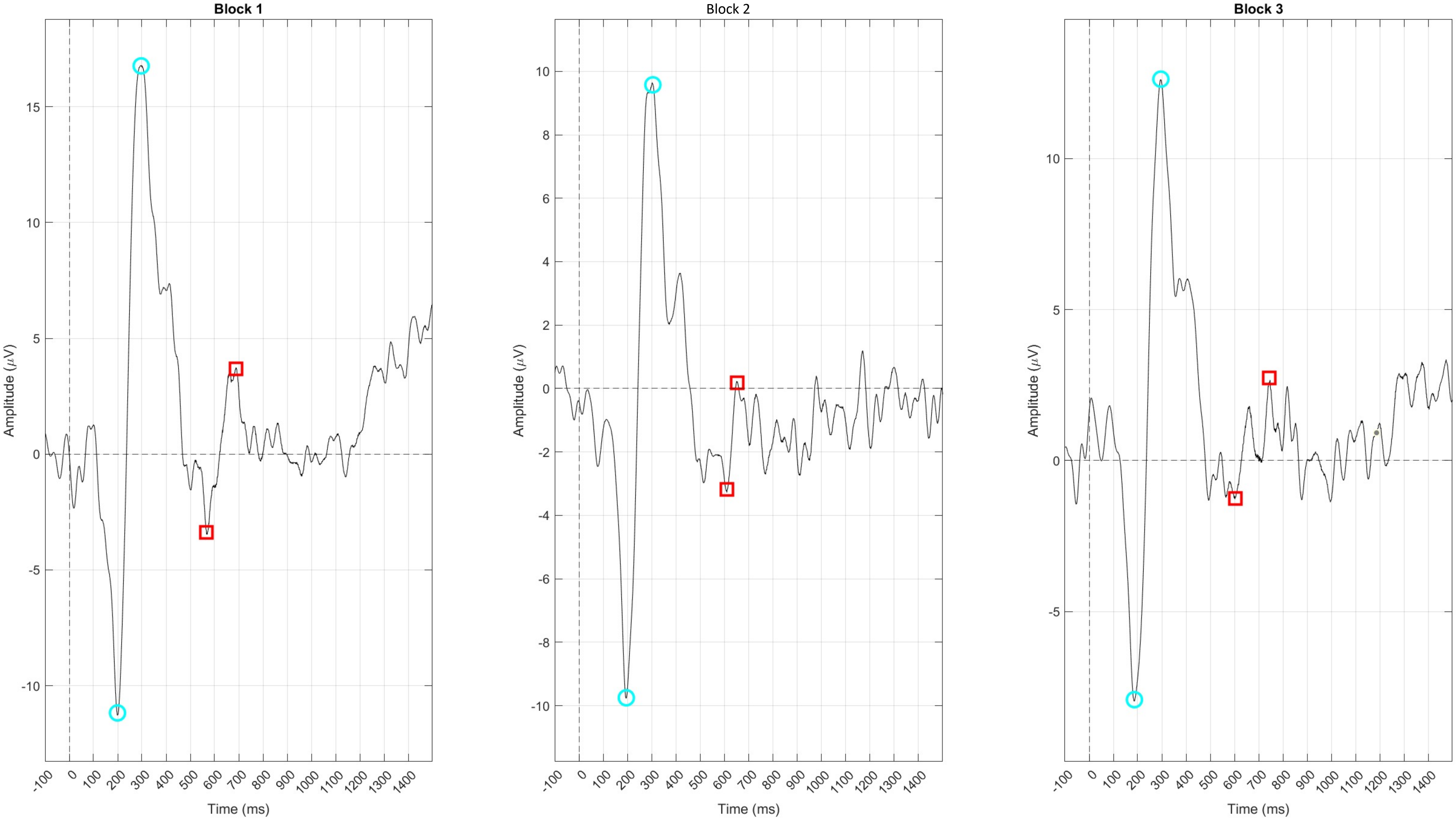

Block 1
Amplitude (μV)
Time (ms)
Block 2
Amplitude (μV)
Time (ms)
Block 3
Amplitude (μV)
Time (ms)

Subject 70

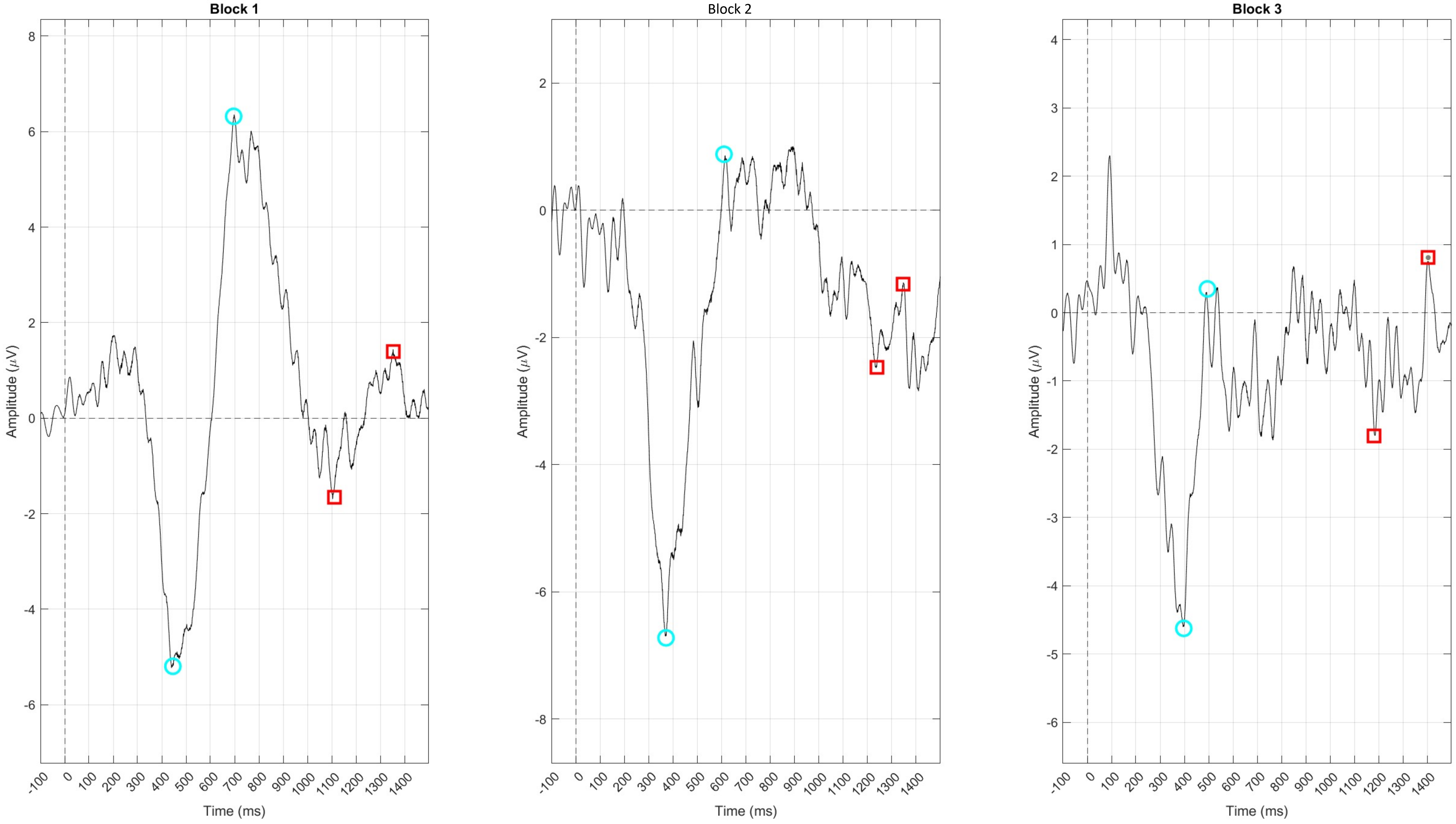

Block 1
Block 2
Block 3
Amplitude (μV)
Time (ms)
-100
0
100
200
300
400
500
600
700
800
900
1000
1100
1200
1300
1400

Subject 71

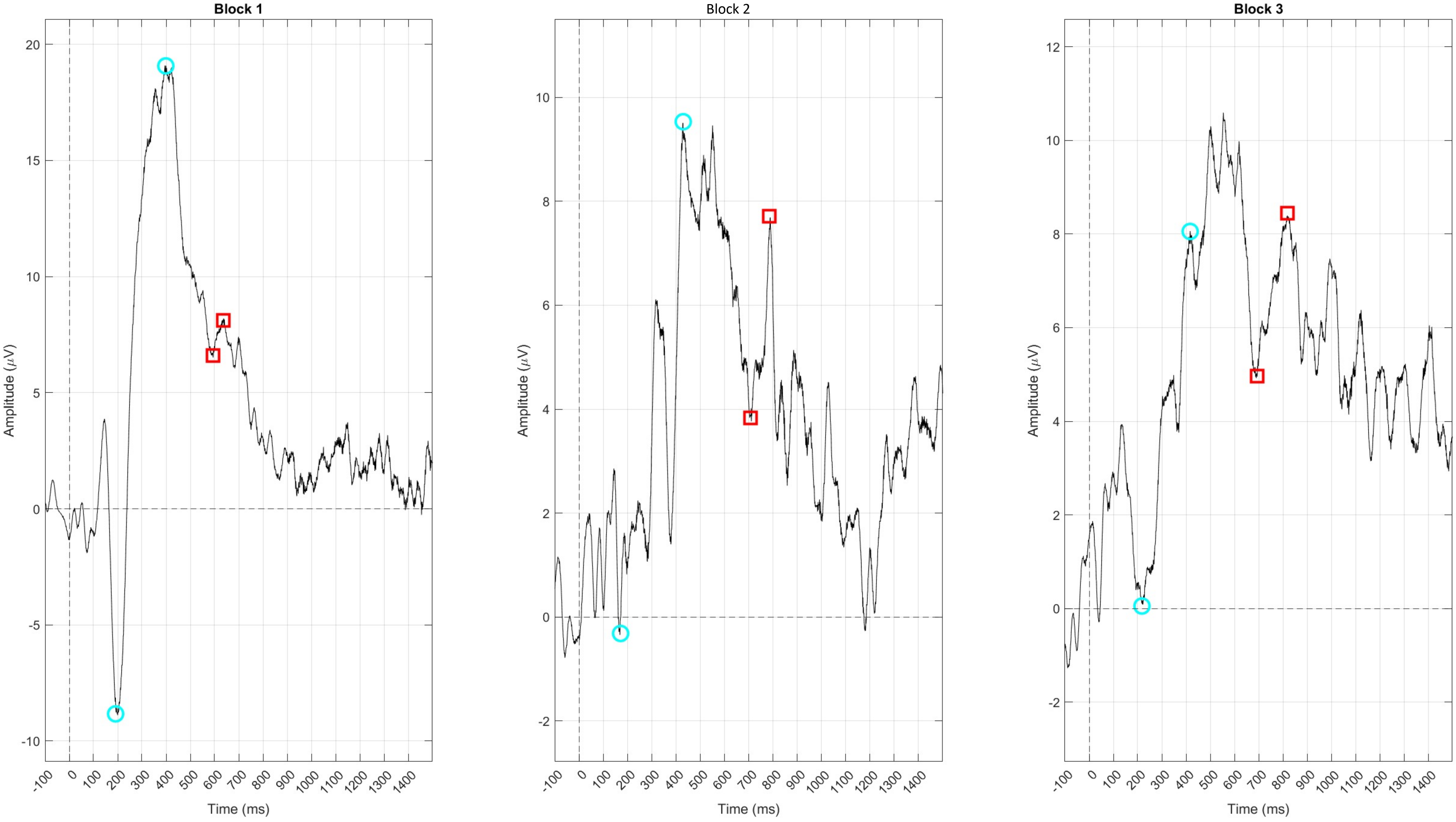
Block 1
Block 2
Block 3
Amplitude (μV)
Time (ms)
-100
0
100
200
300
400
500
600
700
800
900
1000
1100
1200
1300
1400
20
15
10
5
0
-5
-10
12
8
6
4
2
-2

Subject 72

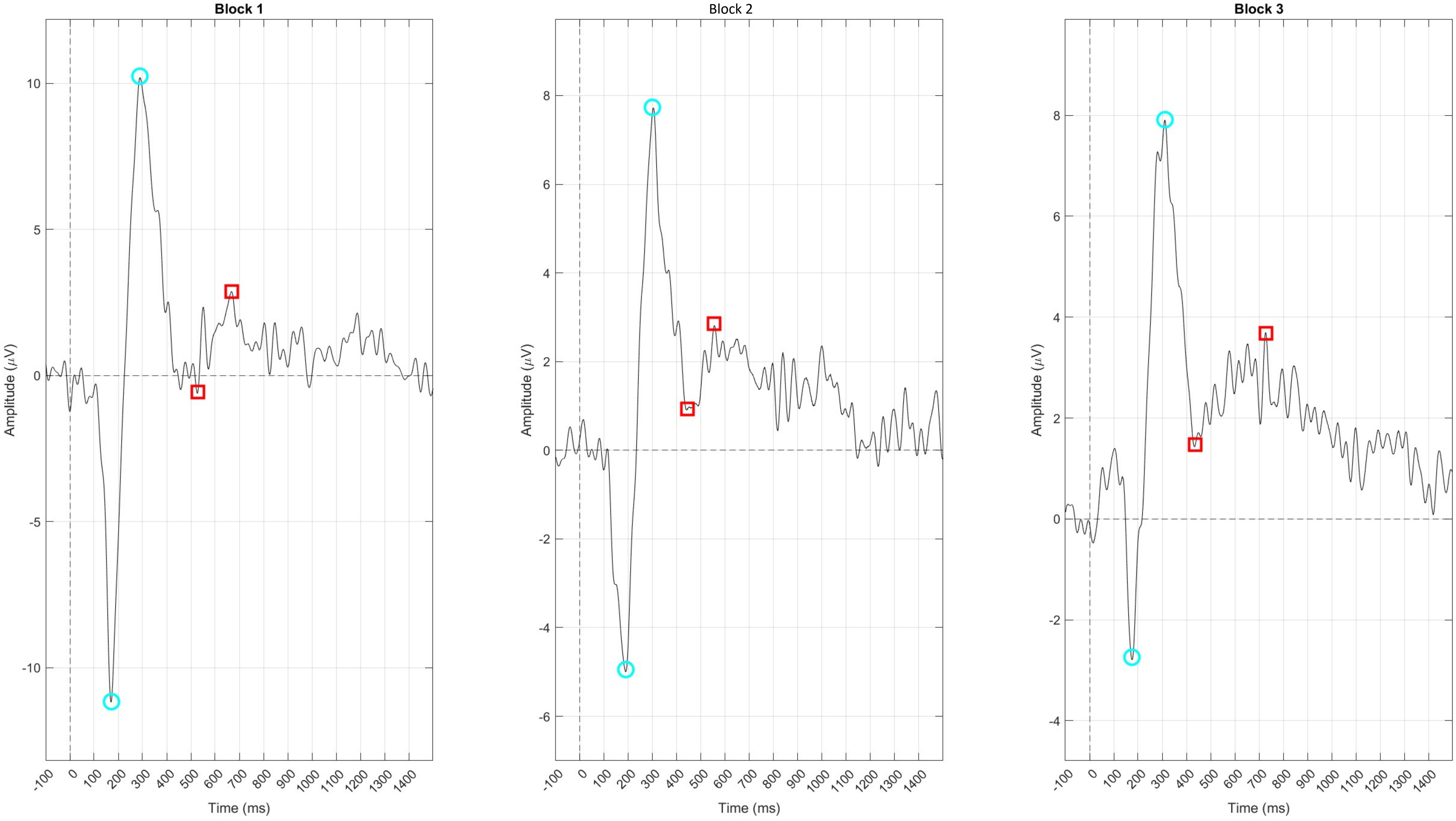
Block 1
Block 2
Block 3
Amplitude (μV)
Time (ms)
-100
0
100
200
300
400
500
600
700
800
900
1000
1100
1200
1300
1400
10
5
0
-5
-10
8
6
4
2
-2
-4
-6

Subject 73

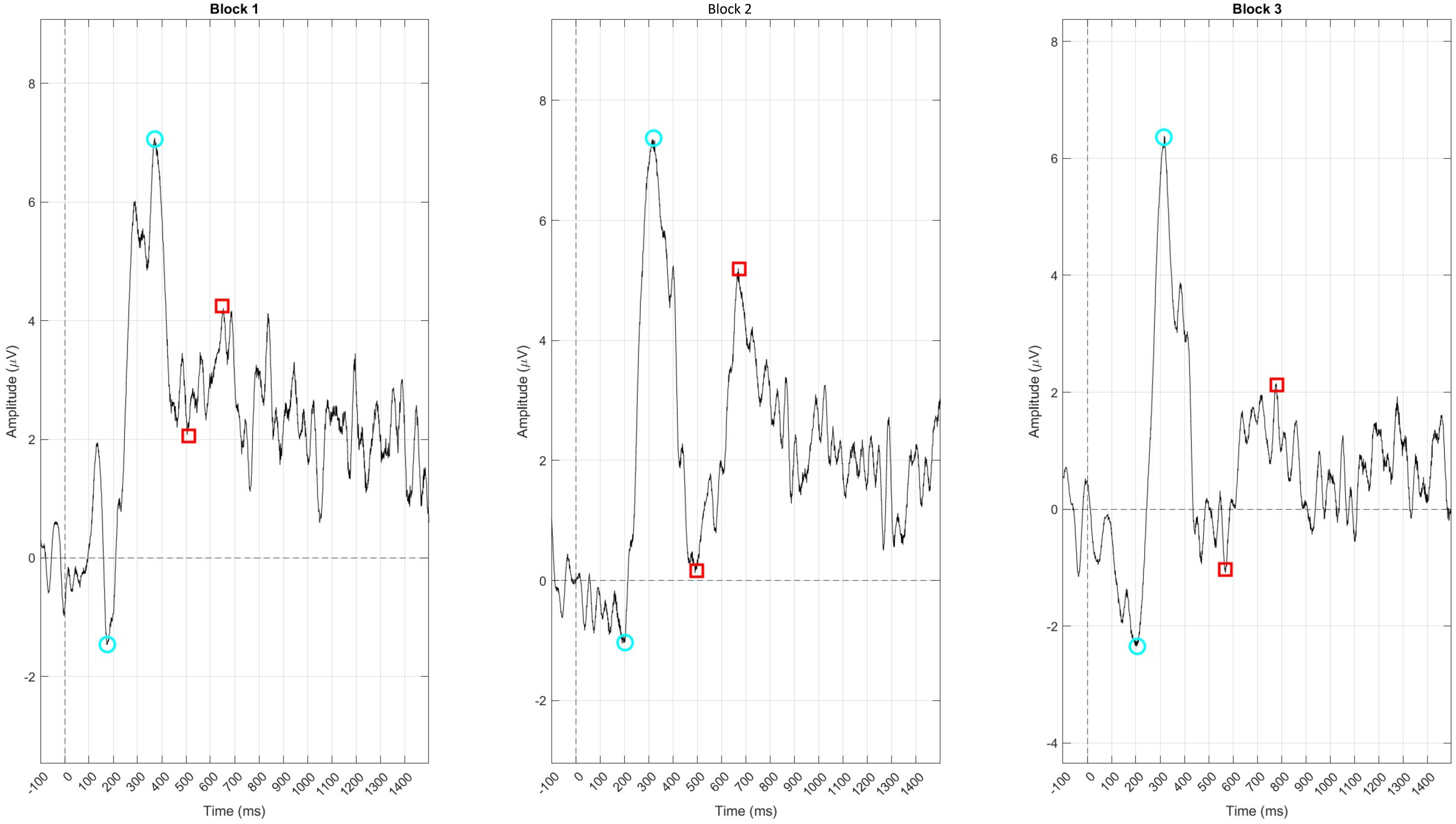

Subject 74 was excluded from analysis

Subject 75

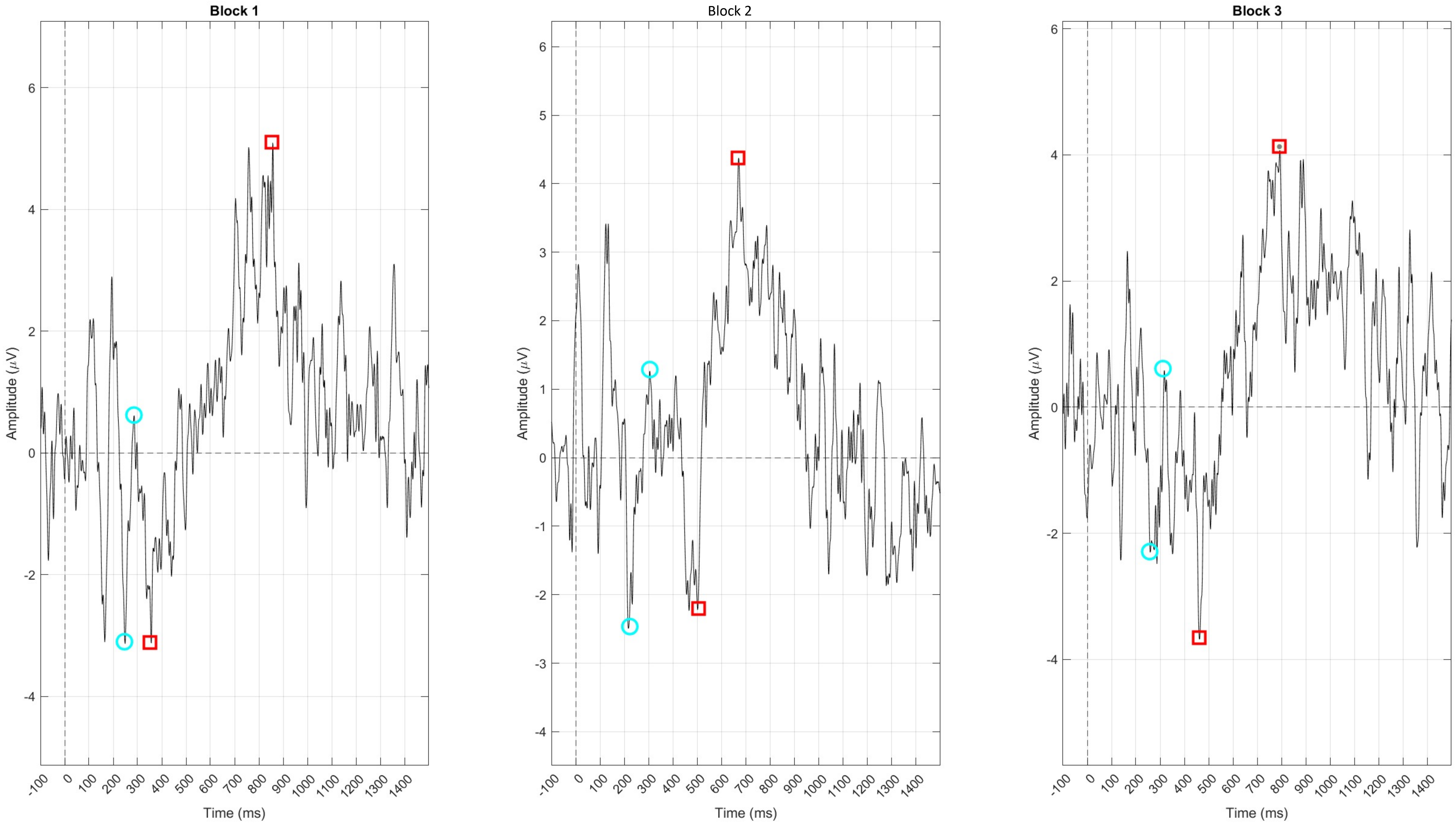

Subject 76

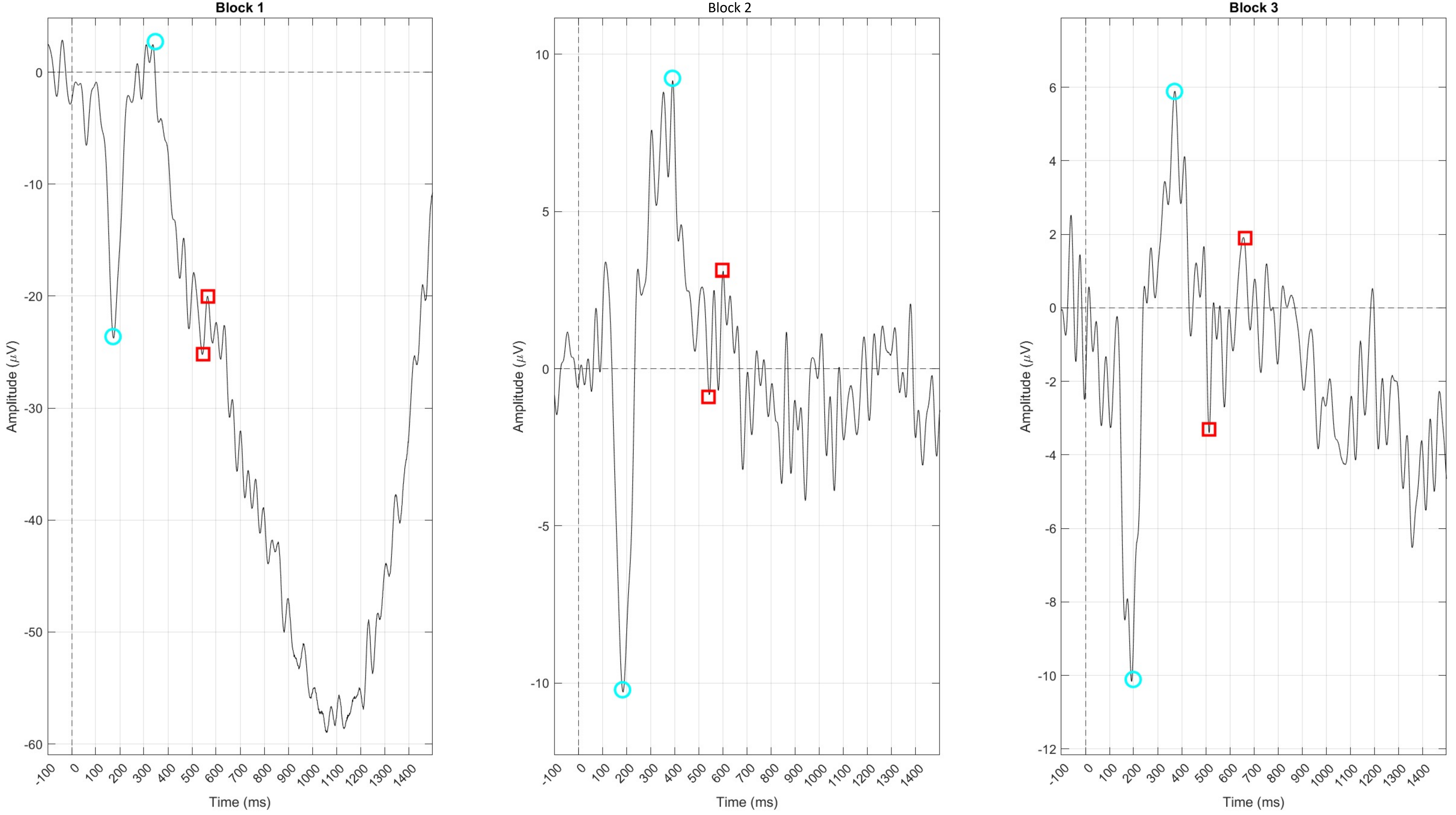

Subject 77

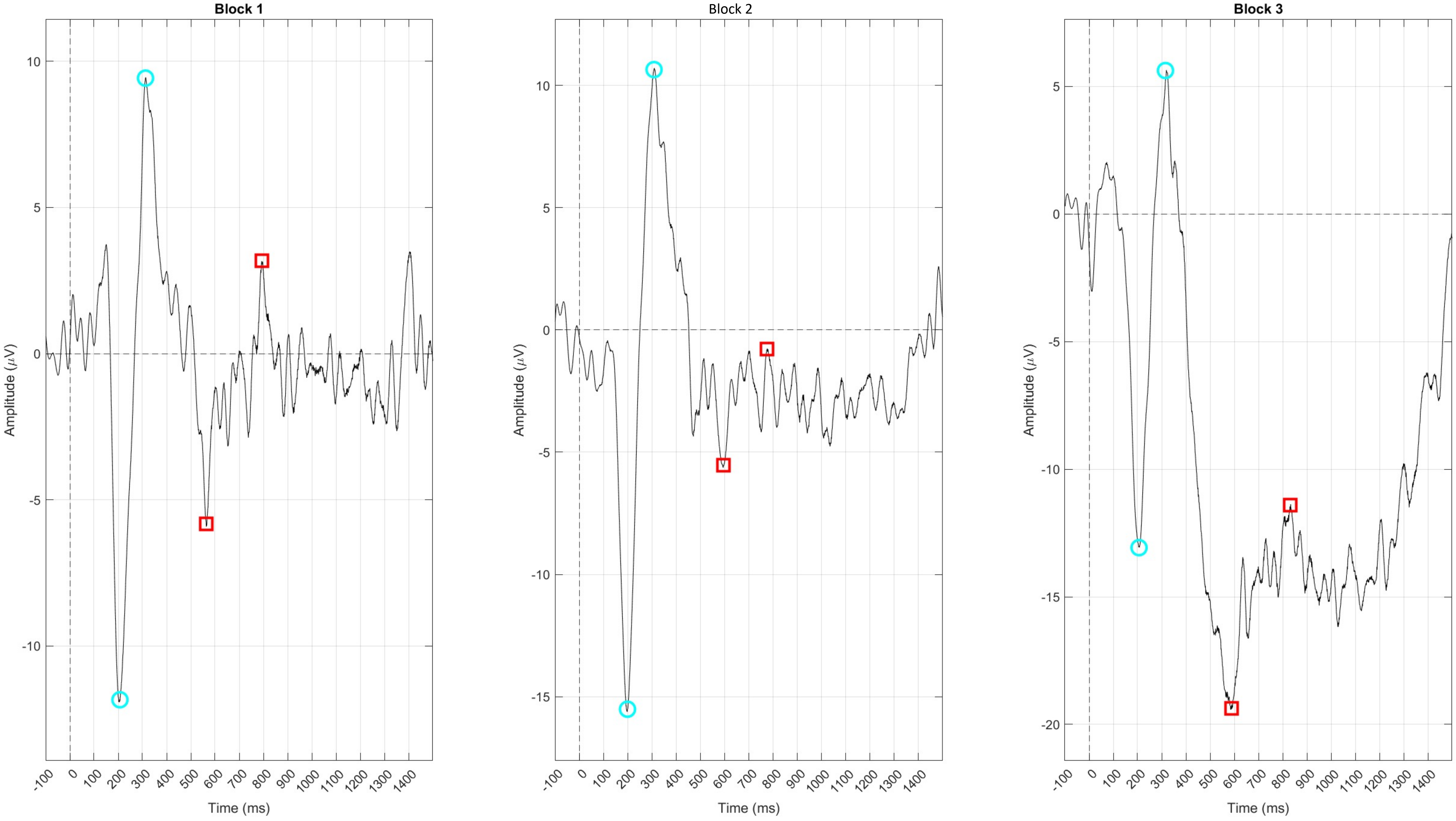

Block 1
Block 2
Block 3
Amplitude (μV)
Time (ms)
10
5
0
-5
-10
-15
-20
-100
0
100
200
300
400
500
600
700
800
900
1000
1100
1200
1300
1400

Subject 78

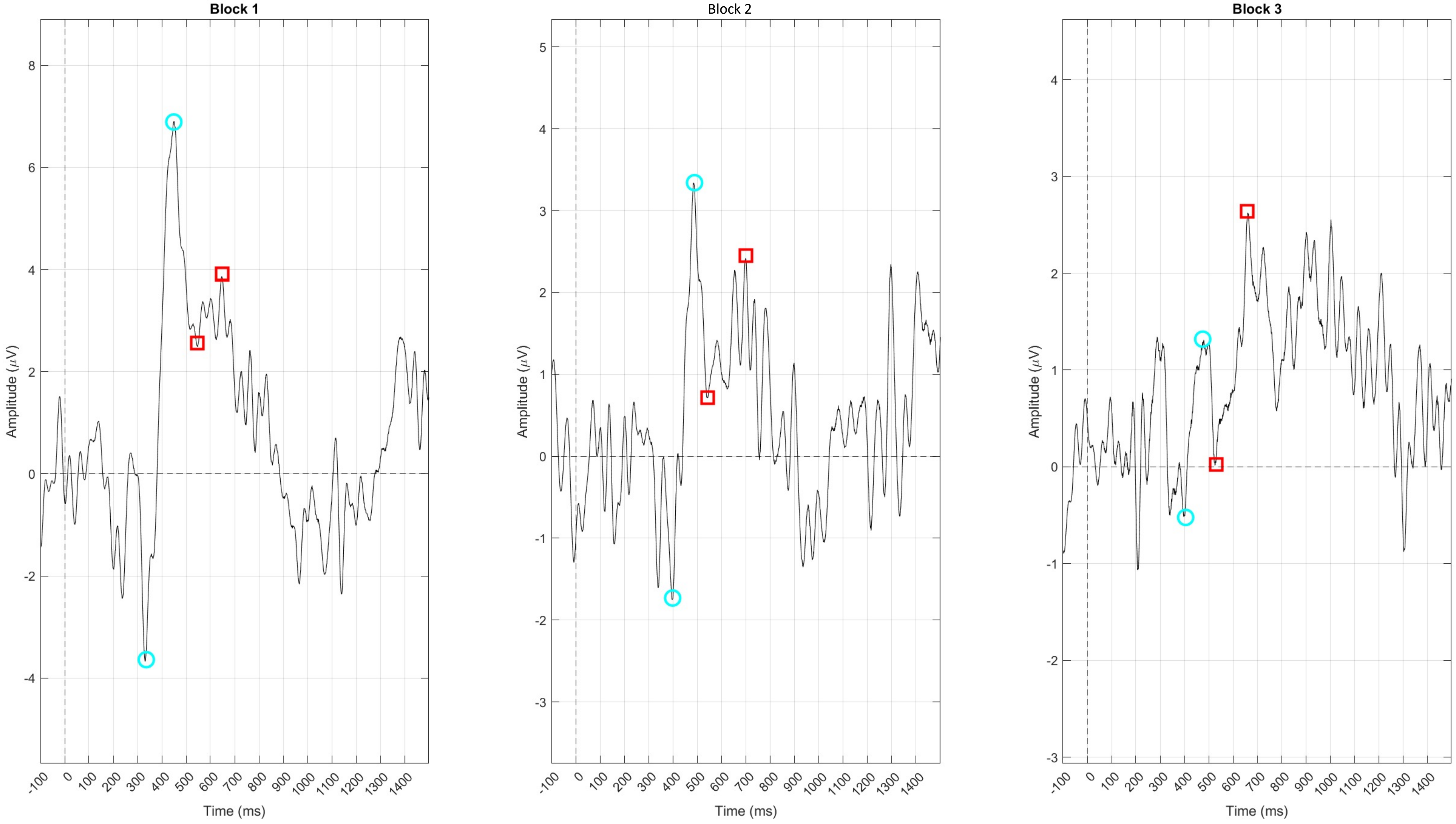
Block 1
Block 2
Block 3
Amplitude (μV)
Time (ms)
8
6
4
2
0
-2
-4
5
3
1
-1
-3
-100
0
100
200
300
400
500
600
700
800
900
1000
1100
1200
1300
1400

Subject 79

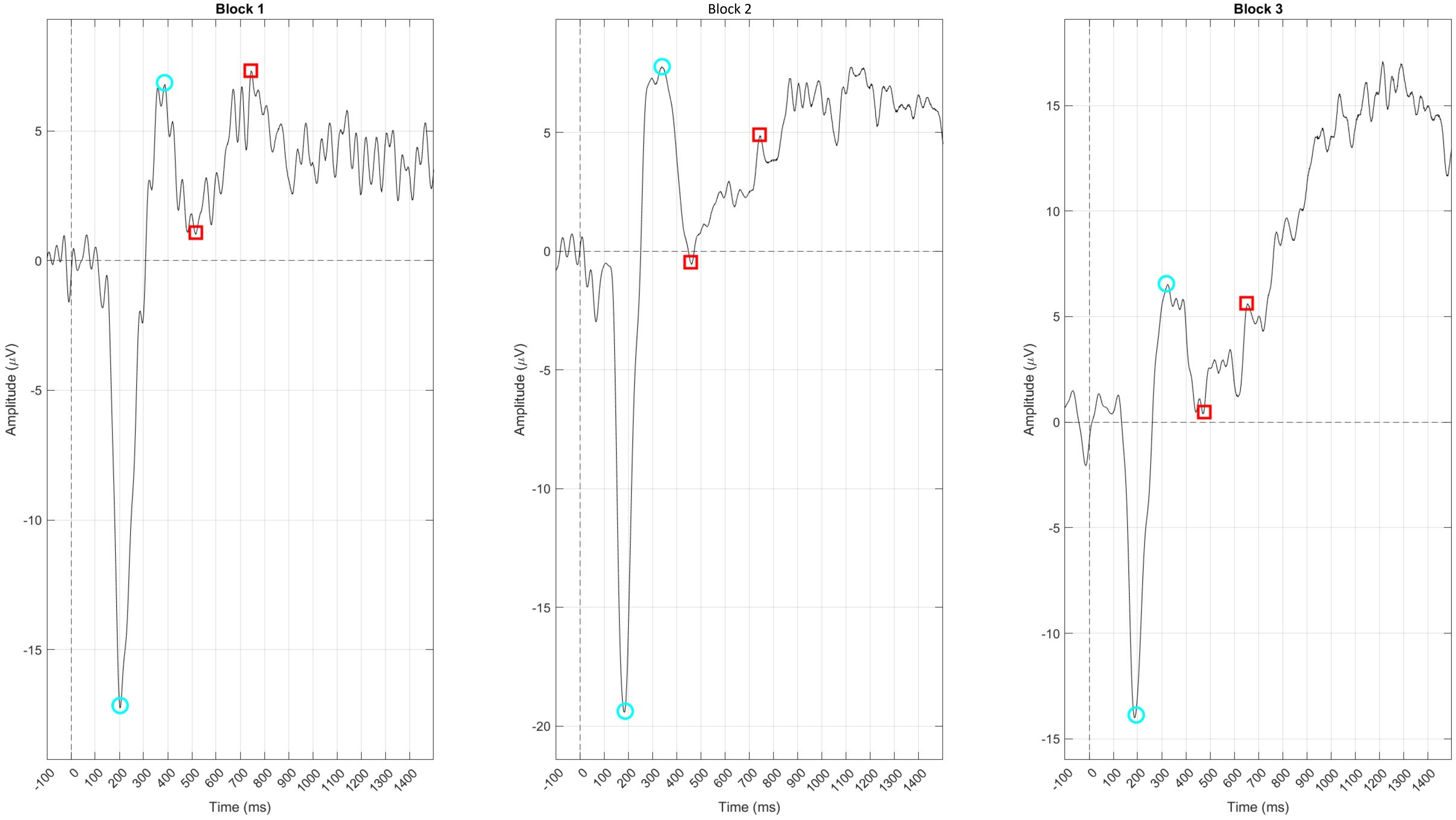
Block 1
Block 2
Block 3
Amplitude (μV)
Time (ms)
-100
0
100
200
300
400
500
600
700
800
900
1000
1100
1200
1300
1400
5
0
-5
-10
-15
-20
10
15

Subject 80

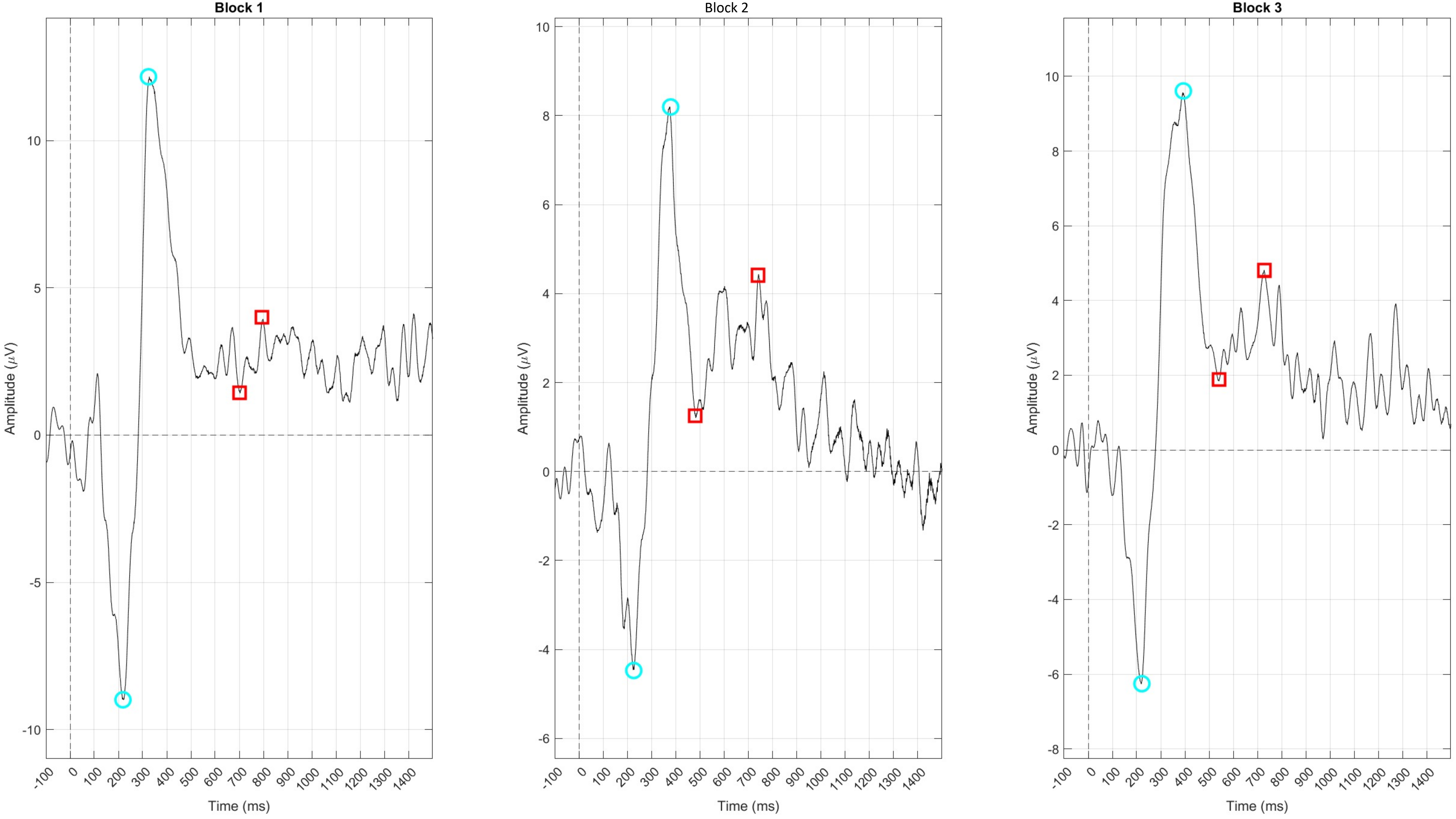
Block 1
Block 2
Block 3
Amplitude (μV)
Time (ms)
-100
0
100
200
300
400
500
600
700
800
900
1000
1100
1200
1300
1400
